# Psychological inoculation improves resilience against misinformation on social media

**DOI:** 10.1126/sciadv.abo6254

**Published:** 2022-08-24

**Authors:** Jon Roozenbeek, Sander van der Linden, Beth Goldberg, Steve Rathje, Stephan Lewandowsky

**Affiliations:** ^1^ Department of Psychology, University of Cambridge, Cambridge, UK.; ^2^ Jigsaw (Google LLC), Mountain View, CA, USA.; ^3^ School of Psychological Science, University of Bristol, Bristol, UK.; ^4^ School of Psychological Sciences, University of Western Australia, Perth, WA, Australia.

## Abstract

Online misinformation continues to have adverse consequences for society. Inoculation theory has been put forward as a way to reduce susceptibility to misinformation by informing people about how they might be misinformed, but its scalability has been elusive both at a theoretical level and a practical level. We developed five short videos that inoculate people against manipulation techniques commonly used in misinformation: emotionally manipulative language, incoherence, false dichotomies, scapegoating, and ad hominem attacks. In seven preregistered studies, i.e., six randomized controlled studies (*n* = 6464) and an ecologically valid field study on YouTube (*n* = 22,632), we find that these videos improve manipulation technique recognition, boost confidence in spotting these techniques, increase people’s ability to discern trustworthy from untrustworthy content, and improve the quality of their sharing decisions. These effects are robust across the political spectrum and a wide variety of covariates. We show that psychological inoculation campaigns on social media are effective at improving misinformation resilience at scale.

## Teaser

Psychological inoculation is effective at reducing misinformation susceptibility on social media.

## INTRODUCTION

Online misinformation is an important societal problem (*1*). For instance, belief in misinformation about coronavirus disease 2019 (COVID-19) has been linked to reduced willingness to get vaccinated against the disease and lower intentions to comply with public health measures (*2*, *3*). In recent years, scientists have looked for ways to effectively counter belief in (*4*, *5*) and sharing of misinformation (*6*). For example, a growing body of research has looked at whether shifting people’s attention toward accuracy can have a positive influence on their news sharing decisions (*6*, *7*). When it comes to tackling misinformation susceptibility and improving people’s resilience against manipulation attempts, however, scalability has remained elusive (*8*).

To further complicate this problem, correcting misinformation after it has spread (for example, through fact-checking) comes with several challenges: Establishing what counts as factual information is epistemologically difficult, particularly in the context of politics (*9*); fact-checks are unlikely to reach everyone who was exposed to the initial misinformation (*10*); getting people to believe fact-checks is challenging (*11*); effective interventions are hard to scale to a population level (*12*); and testing the effectiveness of interventions in the real world (as opposed to a laboratory setting) is complicated (*6*). Debunking misinformation is also problematic because correcting misinformation does not always nullify its effects entirely, a phenomenon known as the “continued influence effect” (*13*).

Accordingly, in contrast to debunking, prebunking has gained prominence as a means to preemptively build resilience against anticipated exposure to misinformation (*4*). This approach is usually grounded in inoculation theory (*14*). Inoculation theory follows a medical immunization analogy and posits that it is possible to build psychological resistance against unwanted persuasion attempts, much like medical inoculations build physiological resistance against pathogens. Psychological inoculation treatments contain two core components (*15*): (i) a forewarning that induces a perceived threat of an impending attack on one’s attitudes and (ii) exposure to a weakened (micro)dose of misinformation that contains a preemptive refutation (or prebunk) of the anticipated misleading arguments or persuasion techniques. However, important open questions remain with regard to inoculation theory and its scalability on social media (*16*), which we address in this study.

First, traditional inoculation research has focused on building resilience against specific persuasive attacks (*15*). However, focusing on the manipulation techniques and rhetorical strategies that underpin misinformation may significantly improve the potential scalability of inoculation interventions (*17*). The advantage of this approach is that, although it can be difficult to establish what is and what is not a fact (*1*), different examples of misinformation often make use of the same underlying tropes (*18*, *19*). These tropes, which include manipulation techniques such as logical fallacies (*20*) and emotionally manipulative language (*18*), can be analyzed and used for inoculation without prior knowledge of specific misleading content, thus potentially providing broad resilience against social media or news content that draws on one or more of the techniques that someone has been inoculated against.

Second, the effectiveness of anti-misinformation interventions in real-world settings remains underexplored (*16*). With respect to interventions that target behavior (such as the sharing of low-quality information), priming people to be more mindful of accuracy (through so-called accuracy nudges or accuracy prompts) was found to prompt Twitter users to share more high-quality sources such as *CNN* and the *New York Times* (*6*). However, how to reduce misinformation susceptibility in real-world environments where misinformation is regularly consumed (such as social media and video sharing platforms) remains an open question (*21*). There is a shortage of studies that test anti-misinformation interventions in an ecologically valid manner, i.e., testing an intervention as consumers would interact with it in online environments (*8*, *12*).

### The present research

We created a series of short inoculation videos covering five manipulation techniques commonly encountered in online misinformation. These five techniques were taken from the broader literature on argumentation and manipulation strategies and are consistently identified as epistemologically dubious: (i) using emotionally manipulative rhetoric to evoke outrage, anger, or other strong emotions (*18*, *22*), (ii) the use of incoherent or mutually exclusive arguments (*23*), (iii) presenting false dichotomies or dilemmas (*24*), (iv) scapegoating individuals or groups (*25*), and (v) engaging in ad hominem attacks (*19*, *26*).

**Fig. 1.**
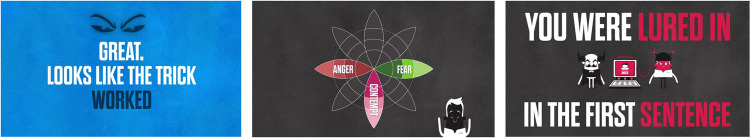
Screenshots from the emotional language video (studies 1 and 6). All videos can be viewed at https://inoculation.science/inoculation-videos/.

Each video instantiates the inoculation procedure by first providing a forewarning of an impending misinformation attack, then issuing a preemptive refutation of the manipulation technique used in this attack, and lastly presenting a “microdose” of misinformation in the form of innocuous and humorous examples (such as an example of incoherence from the animated television series Family Guy). All examples are nonpolitical and fictitious, in addition to being humorous, to avoid any appearance of partisan bias and prevent triggering defensive motivated cognition. The videos can be viewed at https://inoculation.science/inoculation-videos/. Figure 1 shows screenshots from the emotional language video.

We conducted seven preregistered studies to test the effectiveness of these videos as a way to inoculate people against online misinformation: five randomized controlled studies to test the five videos (studies 1 to 5; total *n* = 5416, national quota samples of the United States); a replication of the emotional language study with randomized outcome measure response order (study 6, *n* = 1068); and an ecologically valid field study where we ran two of the videos as an ad campaign on YouTube to test the interventions in a real-world setting (study 7, *n* = 22,632).

In studies 1 to 6, participants were randomly assigned to watch either a 1.5-min inoculation video or a neutral control video of approximately equal length. After watching a video, participants rated 10 synthetic social media posts (mimicking Twitter and Facebook). Each post was randomly either manipulative (i.e., it made use of a manipulation technique) or a neutral counterpart (similar in content and length to the manipulative post but not making use of the manipulation technique). These stimuli were adapted from real-world examples of manipulation techniques and logical fallacies, reworded to fit within a social media post format. The manipulative stimuli were not always necessarily false in the sense that they made verifiably untrue claims; rather, they were designed to clearly make use of a specific manipulation technique. Participants saw an average of five manipulative and five neutral stimuli, although this varied per participant because randomization took place at the stimuli level. See fig. S7 for an overview of some of the stimuli used in studies 1 to 6. For deviations from our preregistrations, see notes i and ii in the Supplementary Materials.

We included four measures for each of the stimuli: participants’ ability to recognize manipulation techniques in social media content (*17*); their confidence in their ability to recognize these techniques (*27*); their ability to distinguish trustworthy from untrustworthy content (*28*); and the quality of their sharing decisions, i.e., willingness to share (*6*). Our main outcome variable of interest for the technique recognition, trustworthiness, and sharing measures [but not confidence (see note iii in the Supplementary Materials)] is discernment, i.e., the difference between averaged scores for the manipulative and neutral stimuli; higher discernment indicates an increased ability to discern manipulative from nonmanipulative content for each outcome measure (*6*). For example, higher trustworthiness discernment means a higher accuracy in telling apart trustworthy and untrustworthy social media content. See Materials and Methods for our preregistered hypotheses and further details about our stimuli and study design.

## RESULTS

### Studies 1 to 5: Testing the interventions in the laboratory

**Fig. 2.**
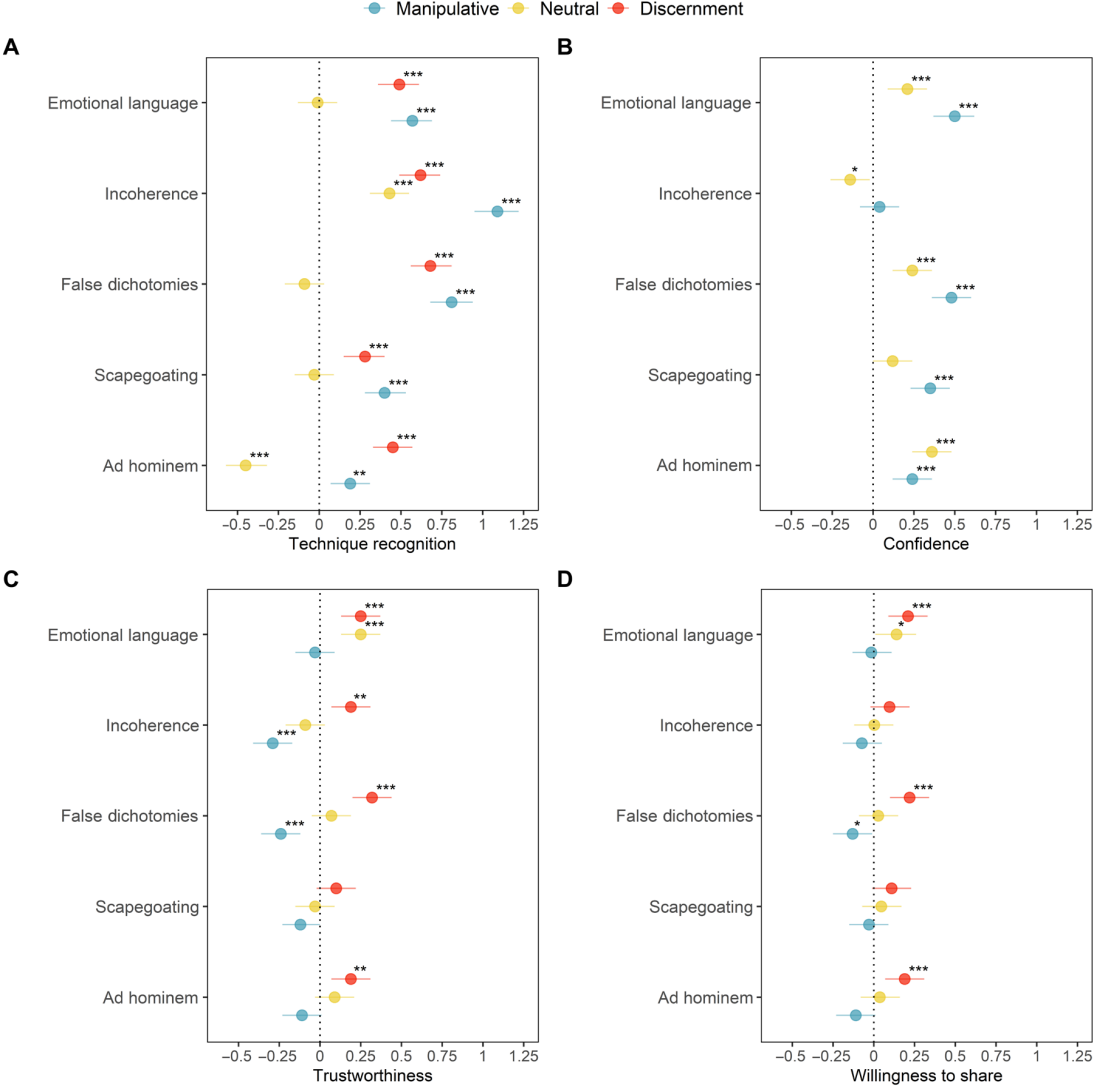
Studies 1 to 5: Dot plots for effect sizes (Cohen’s *d*) from independent-samples *t* tests. Technique recognition (**A**), confidence (**B**), trustworthiness (**C**), and sharing (**D**), by condition and study, for manipulative social media content, neutral content, and discernment (discernment indicates the difference score between manipulative and neutral content; not shown for the confidence measure). Improved discernment indicates a higher ability to distinguish manipulative from neutral stimuli. Error bars show 95% confidence intervals for Cohen’s *d*. ^***^P < 0.001, ^**^P < 0.01, and ^*^P < 0.05.

We present the results for studies 1 to 5 separately by outcome measure (technique recognition, confidence, trustworthiness, and willingness to share) in Fig. 2 and Table 1. To clarify whether improved discernment is driven by participants’ ratings of manipulative or neutral social media posts (or both), Fig. 2 also shows the results for the manipulative and neutral stimuli separately. See tables S3 to S22 for the full statistical analyses (including Bayesian analyses) and stimuli- and item-level results.

**Table 1.** Studies 1 to 5: *P* values and effect sizes (Cohen’s *d*) from independent-samples *t* tests. Dependent variables are technique recognition (discernment), confidence (manipulative social media content only), trustworthiness (discernment), and sharing (discernment). The independent variable is the experimental condition (inoculation vs. control). *P* values are uncorrected for multiple comparisons. Significant *P* values are marked in bold.

**Study**	**Technique recognition**		**Confidence**		**Trustworthiness**		**Sharing**	
	***P* value**	**Cohen’s *d***	***P* value**	**Cohen’s *d***	***P* value**	**Cohen’s *d***	***P* value**	**Cohen’s *d***
1 - Emotional language	**<0.001**	0.49	**<0.001**	0.50	**<0.001**	0.25	**<0.001**	0.21
2 - Incoherence	**<0.001**	0.62	0.471	0.04	**0.002**	0.19	0.109	0.10
3 - False dichotomies	**<0.001**	0.68	**<0.001**	0.48	**<0.001**	0.32	**<0.001**	0.22
4 - Scapegoating	**<0.001**	0.28	**<0.001**	0.35	0.100	0.10	0.067	0.11
5 - Ad hominem	**<0.001**	0.45	**<0.001**	0.24	**0.002**	0.19	**<0.001**	0.19

Figure 2 and Table 1 show that our preregistered hypotheses (see Materials and Methods) are supported for 16 of 20 outcome measures, except for the confidence and sharing measures for the incoherence video (study 2) and the trustworthiness and sharing measures for the scapegoating video (study 4) (see note iv in the Supplementary Materials). We thus find that watching an inoculation video improves people’s ability to recognize manipulation techniques in social media content and increases their confidence in their ability to do so. In addition, the videos improve people’s ability to distinguish trustworthy and untrustworthy content, as well as the quality of their sharing decisions (i.e., they are either less likely to share manipulative content with others or more likely to share neutral/non-manipulative content). Our supplementary analyses show that the findings presented here are robust; see Supplementary Analyses and tables S3 to S22, S23 to S32, and S44 to S48 (see note v in the Supplementary Materials). The relative reduced effectiveness of the scapegoating and incoherence videos compared to the other three videos may be explained by variations in baseline discernment across techniques (i.e., participants in the control group were better at identifying the scapegoating/incoherence technique than other techniques even without an intervention) (see note vi in the Supplementary Materials).

### Study 6: Replication and order effects

Study 6 had two goals: (i) to replicate the findings from study 1 (the emotional language video) 1 year after it was originally conducted and (ii) to check whether manipulating the order in which participants respond to the outcome measures for each of the stimuli (technique recognition, trustworthiness, and sharing) influences the results. This is important because eliciting (for example) the manipulativeness and/or trustworthiness of a survey item before willingness to share might influence the responses participants give for the sharing measure, as participants may be primed to think about the item’s manipulativeness before providing their sharing intentions (*29*, *30*). Therefore, alongside the experimental condition, participants were randomly assigned to one of three response orders [manipulativeness – trustworthiness – sharing (MTS), *n* = 364; trustworthiness – sharing – manipulativeness (TSM), *n* = 361; or sharing – manipulativeness – trustworthiness (SMT); *n* = 343]; see Materials and Methods for more details.

We replicate the results from study 1: Participants in the inoculation group have significantly higher discernment than the control group for technique recognition (*P* < 0.001, *d* = 0.67), trustworthiness (*P* < 0.001, *d* = 0.44), and sharing (*P* < 0.001, *d* = 0.34); see table S49. Second, we find that outcome measure response order did not significantly interact with the experimental condition for any of the three outcome measures (all *P* values > 0.351; see table S50). Furthermore, discernment is significantly higher in the inoculation condition than the control condition for all three outcome measures when looking at each of the three response orders individually (all *P* values < 0.003; see table S51), indicating that varying the response order of the outcome measures beneath the stimuli does not meaningfully influence the results.

### Studies 1 to 6: Moderation analyses

Recent work has emphasized the role of individual-difference variables in misinformation susceptibility, foremost among them partisan bias, actively open-minded thinking, “bullshit receptivity,” and analytical thinking ability (*31*–*33*). Crucially, some of these factors (particularly political partisanship) are known to moderate the effectiveness of some anti-misinformation interventions, such as accuracy nudges (*34*).

We therefore ran an extensive set of preregistered moderation analyses, to see whether the effects reported in studies 1 to 6 are robust to the inclusion of covariates and to check whether participants from a wide variety of backgrounds can be successfully inoculated against manipulation techniques (as noted in note i in the Supplementary Materials, we preregistered that we would conduct three-way interactions to test for moderation effects; this was an error on our part as there are only two interacting variables: experimental condition and the covariate. We therefore test for two-way interactions, alongside a series of other moderation analyses). We included the following variables: gender, age, education, political ideology, how often people check the news, social media use, populism (*35*), “bullshit” receptivity (*36*), conspiracy belief (*37*), analytical thinking (as measured by the cognitive reflection test or CRT) (*38*), numeracy skills (*2*), the 10-item OCEAN personality inventory (*39*), actively open-minded thinking (*33*, *40*), and misinformation susceptibility as measured by the misinformation susceptibility test (*33*, *41*).

We find that there are no consistently significant two-way interactions between the experimental condition and each of the covariates. The inoculation effect conferred by the videos is therefore robust when controlling for a wide variety of potential moderators. See Materials and Methods for more details, as well as tables S23 to S27, S38 to S48, and S52 to S54 for the regression tables and figs. S2 to S6 for visualizations (see note vii in the Supplementary Materials).

### Study 7: Testing the interventions on YouTube

In study 7, we implemented two of the inoculation videos (emotional language and false dichotomies; these videos were chosen because of their robust effects from studies 1 to 6) as advertisement campaigns on the video sharing platform YouTube, the world’s second-most visited website (*42*). There has been widespread concern about the consumption of conspiratorial and false content on YouTube (*43*, *44*). For example, Alfano *et al*. (*43*) find that YouTube’s recommender system tends to recommend extremist content by so-called “gurus,” who regularly make use of manipulation techniques such as those addressed in the videos from studies 1 to 6. YouTube therefore provides an ideal ecology to test the effectiveness of anti-misinformation interventions in an environment in which people are regularly exposed to false or manipulative content. Unlike social media platforms such as Twitter, Reddit, and Facebook, YouTube does not make behavioral data (such as video viewing times) publicly available; our field study therefore focused on reducing misinformation susceptibility (rather than behavioral measures such as sharing behavior). As part of this study, a total of around 967,000 YouTube users watched one of the inoculation videos.

**Fig. 3.**
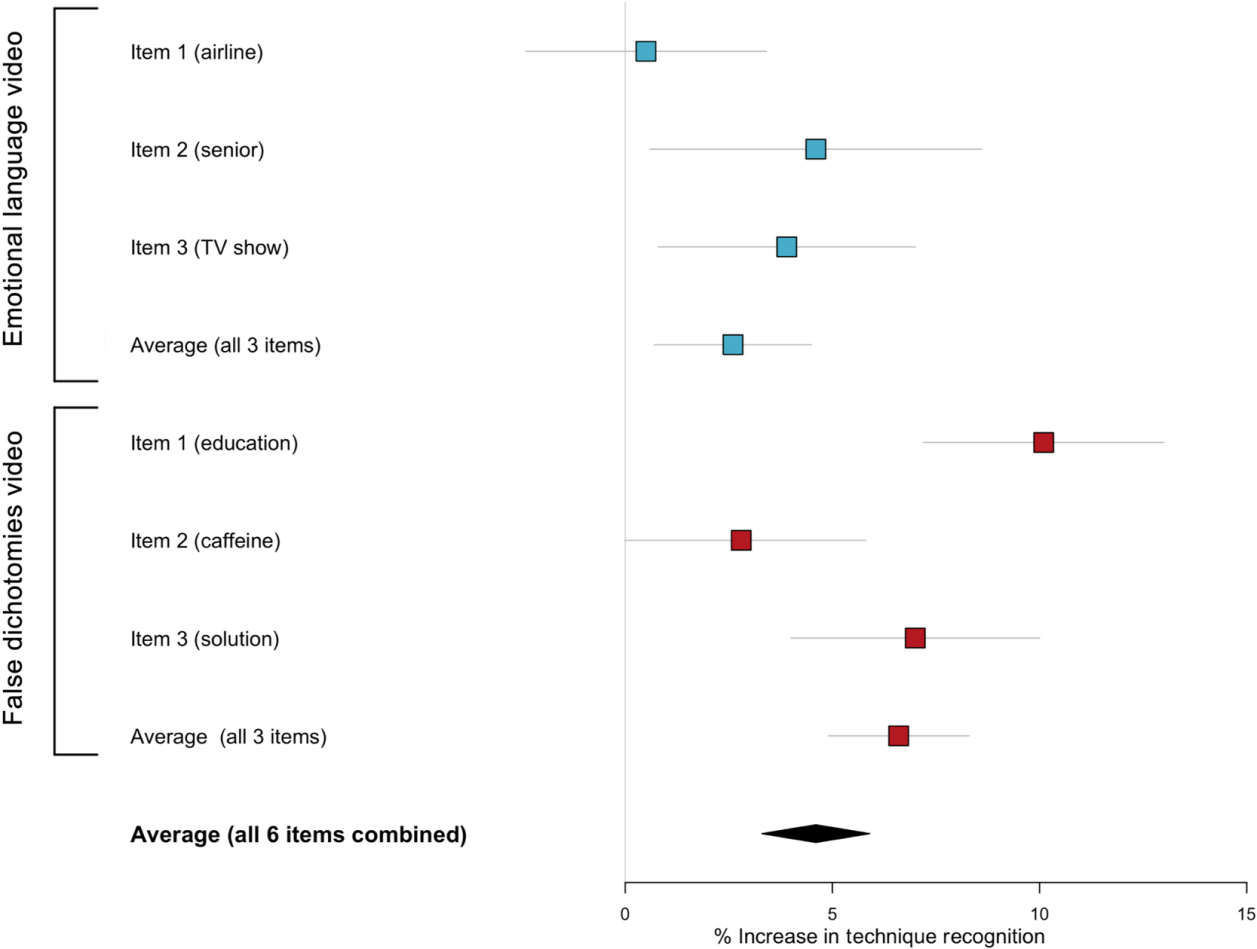
Results for the YouTube field experiment (study 7), showing the average percent increase in manipulation techniques recognized in the experimental (as compared to control) condition. Results are shown separately for items (headlines) 1 to 3 for the emotional language and false dichotomies videos, as well as the average scores for each video and the overall average across all six items. See Materials and Methods for the exact wording of each item (headline). Error bars show 95% confidence intervals.

The treatment group was shown one of the two inoculation videos as a YouTube ad. At some point within 24 hours after watching the ad, a random 30% of this group was shown one single-item test question embedded within the YouTube environment, which consisted of a headline containing a particular manipulation technique (the headlines were adapted from the stimuli used in studies 1 and 3). Participants were asked which (if any) manipulation technique the headline contained (“This headline contains…,” with four response options, only one being correct). For example, item 1 (“education”) for the false dichotomies video read “We either need to improve our education system or deal with crime on the streets.” We administered three such headlines per video, for a total of six, with each participant only seeing a single test question (see note viii in the Supplementary Materials). We then tested whether people who watched an inoculation video were significantly better than a control group (a group of YouTube users matched to the treatment group in terms of demographics, who were not shown an inoculation video but did answer one of the test questions) at correctly identifying the use of a particular technique in a headline. We collected a total of 22,632 responses (*n*_treatment_ *=* 11,432, *n*_control_ *=* 11,200). See Materials and Methods for the test questions (headlines) used and fig. S8 for examples of how these were administered within the YouTube environment. We refer to table S55 for the full statistical analyses and table S56 for details about the cost and implementation of the ad campaigns. The results are plotted in Fig. 3.

Two-tailed two-proportion *z* tests show that the proportion of correct answers to all six headlines combined is significantly higher in the treatment compared to the control condition (*P* < 0.001, Cohen’s *h* = 0.09). This is true for both the emotional language video (*P* = 0.009, *h* = 0.05) and for the false dichotomies video (*P* < 0.001, *h* = 0.13). Looking at the individual items, the intervention is nonsignificant for the first emotional language item (*P* = 0.757, *h* = 0.01) but is significant for item 2 (*P* = 0.023, *h* = 0.09) and item 3 (*P* = 0.016, *h* = 0.08). For false dichotomies, the intervention is significant for item 1 (*P* < 0.001, *h* = 0.21), nearly significant for item 2 (*P* = 0.073, *h* = 0.06), and significant for item 3 (*P* < 0.001, *h* = 0.13). Overall, although we find significant effects for both videos, we narrowly failed to reach our preregistered smallest effect size of interest (SESOI) of *h* = 0.10, with an observed effect size of *h* = 0.09 across all six items. When considering each video on its own, the SESOI was not met for the emotional language video but it was met for the false dichotomies video (see note ix in the Supplementary Materials).

## DISCUSSION

Across seven high-powered preregistered studies including a field experiment on YouTube, with a total of nearly 30,000 participants, we find that watching short inoculation videos improves people’s ability to identify manipulation techniques commonly used in online misinformation, both in a laboratory setting and in a real-world environment where exposure to misinformation is common.

Nineteen of 23 hypothesized effects from studies 1 to 6 were significant, and three of the four effects that were not significant were in the hypothesized direction. The effect sizes for technique recognition range from Cohen’s *d* = 0.28 (for the scapegoating study) to *d* = 0.68 (for the false dichotomies study), which is substantial, particularly considering the relatively short duration of the interventions. These effect sizes are also generally larger than those of existing scalable interventions. For comparison, digital literacy tips (consisting of a short text pointing out various ways to spot false news, such as looking at the validity of the source) were found to improve detection of false headlines with a Cohen’s *d* of 0.20 (*45*).

Our findings advance misinformation research considering that we used an technique-based approach, in which people were trained in a neutral environment to recognize a manipulation technique that they were subsequently asked to identify in unfamiliar social media posts. Crucially, we replicated these results in an ecologically valid setting on YouTube, boosting manipulation technique recognition by about 5% on average, although people might be facing competing or nonaccuracy-based incentives on the platform. In addition, the interventions are highly affordable: The average cost for each ad view was approximately $0.05. We optimized our campaign for survey responses and avoiding repeat views; over a period of 15 days, the videos were shown to about 5.4 million people, or around 350,000 per day (see table S56). When optimizing for views (rather than survey responses) and with a larger budget, it is very doable to reach millions of people. Social media companies could furthermore offer ad credits to run inoculation campaigns on their platforms, thus reducing the cost even further. Our field study thus confirms that inoculation videos could be run as public-service ads ahead of potentially harmful content and thereby easily scaled across millions of users. Inoculation videos can be applied in a wide range of issue domains, including reducing susceptibility to radicalizing content (*5*).

Nonetheless, our study is not without limitations. First, although our field study found that the videos were effective within a 24-hour window (with a median time of 18.4 hours between watching the video and responding to the survey question), we were unable to study how long the inoculation effect remains significant (*46*, *47*). Second, both the videos and the study were tailored to U.S. audiences, and our samples consisted of U.S. residents. We are therefore not able to assess to what extent inoculation treatments transfer to different cultural and linguistic settings. Third, we were unable to investigate to what extent watching one inoculation video confers psychological resistance against manipulation techniques against which people were not specifically inoculated, a phenomenon known as “cross-protection” (*48*). Lewandowsky and Yesilada (*5*), for instance, found that inoculation videos generated resilience against both Islamist and Islamophobic content, suggesting that cross-protection is feasible. Fourth, the manipulative and neutral stimuli used in studies 1 to 6 (see Materials and Methods) were designed as manipulative-neutral counterparts, where the manipulative stimuli make use of a manipulation technique and the neutral stimuli do not. Although we made effort to ensure that these stimuli pairs are as similar as possible and only differ in their use (or nonuse) of a manipulation technique, they are not each other’s exact mirror, and we cannot rule out that the stimuli differ on other dimensions as well. Fifth, participants in our YouTube field study each only saw a single test item, as it was only possible to embed a single question at the end of YouTube videos (see Materials and Methods). We tried to mitigate potential item effects by using multiple test questions per inoculation video across participants. Last, our YouTube field study only assessed misinformation susceptibility and not people’s behavior (such as the sharing of misinformation with others or for how long people watch low-quality content on YouTube). YouTube does not make behavioral data publicly available, unlike Twitter and other social media platforms, and so we were unable to take behavioral measures into account. We encourage future work that addresses these limitations.

In sum, we provide strong evidence that technique-based inoculation videos can confer psychological resistance against manipulation techniques commonly encountered in online misinformation: the use of excessively emotional language, incoherence, false dichotomies, scapegoating, and ad hominem attacks. We show that these interventions are effective for people with different ideological backgrounds and cognitive styles and are generalizable to a wide variety of key issue domains. We provide evidence that these videos are effective not only in a laboratory setting but also “in the wild” on a video sharing platform and can therefore be easily implemented at scale to improve resilience against misinformation at a cost of about $0.05 per video view. Our findings are thus a significant step forward in our understanding of individual susceptibility to online misinformation and how to prevent people from being misled.

## MATERIALS AND METHODS

For this study, we designed five short, animated inoculation videos in partnership with Google Jigsaw, each exposing a manipulation technique commonly encountered on social media and in other online environments. These videos were designed to “inoculate” people against being misled by flawed argumentation used in common online misinformation, such as excessively emotional language with an aim to invoke anger or outrage (*22*). In seven preregistered studies (and one pilot study), we tested the efficacy of each of these videos using a randomized controlled design. The pilot study (*n* = 194; see table S2 for the main results) was conducted with the emotional language video to validate our stimuli sets and outcome measures. The only difference of note between the pilot and the final studies is that in the pilot, we used “credibility” instead of “trustworthiness” as the third outcome measure (the reason for changing it from credibility to trustworthiness is because this term is associated with source or messenger credibility, and since we removed all source information from our stimuli, pilot study participants may have found the use of this outcome measure somewhat confusing). Additional information including the full datasets, analysis and visualization scripts, Qualtrics surveys, and our stimuli can be found on our OSF page: https://osf.io/3769y/. The videos can be viewed on https://inoculation.science.

The methodology for studies 1 to 6 is the same: The treatment (inoculation) group watched one of the “inoculation” videos, and the control group watched a video of similar length and aesthetic, but with content unrelated to online misinformation (specifically: a *SciShow* video about freezer burn, found here: www.youtube.com/watch?v=fPEtOaGTZ0s&t=8s). After watching the video, each group conducted a posttest (in the form of an item test; see the “Outcome measures” section below) and answered a series of demographic and other questions. For study 7 (the YouTube field study), we ran the emotional language video from studies 1 and 6 and the false dichotomies video from study 3 as YouTube advertisements. These “Trueview pre-roll” advertisements were shown at the beginning of a YouTube video that a participant chose to watch (we did not have any control over which specific videos the ads were shown ahead of). For more information about how this process works, see https://support.google.com/displayspecs/answer/6055025?hl=en. We tested the following preregistered hypotheses for studies 1 to 7:

H1: Participants in the treatment (inoculation) group are significantly better than a control group at discriminating social media content containing a manipulation technique and neutral content (technique recognition) (studies 1 to 6).

H2: Participants in the treatment group are significantly more confident in their judgments (i.e., their ability to discern manipulative from nonmanipulative content) than a control group (confidence) (studies 1 to 5).

H3: Participants in the treatment group are significantly better than the control group at discriminating the trustworthiness of manipulative from neutral social media content (trustworthiness) (studies 1 to 6).

H4: Participants in the treatment group are significantly less likely to indicate being willing to share manipulative social media with people in their network than neutral content, compared to a control group (sharing) (studies 1 to 6).

H5: Participants who watch an inoculation video on YouTube as an advertisement (treatment group) are significantly better than those who do not watch an inoculation video (control group) at identifying manipulation techniques in social media content (technique recognition) (study 7).

The preregistrations can be found here: https://aspredicted.org/5mp82.pdf (pilot study); https://aspredicted.org/fb96x.pdf (study 1; emotional language); https://aspredicted.org/jd4v6.pdf (study 2; incoherence); https://aspredicted.org/cm2f6.pdf (study 3; false dichotomies); https://aspredicted.org/js9ci.pdf (study 4; scapegoating); https://aspredicted.org/d67m5.pdf (study 5; ad hominem); https://aspredicted.org/ci5un.pdf (study 6; emotional language replication study); and https://aspredicted.org/9pa96.pdf (study 7, YouTube field study). For studies 1 to 5, we preregistered that we would first collect a pilot sample of *n* = 200 responses (halting data collection at this point and analyzing the pilot data) to ensure that the survey was properly implemented and responses were collected correctly. After ensuring this, we resumed data collection for the remaining 884 target responses. For deviations from our preregistrations, see notes i and ii in the Supplementary Materials. For the ad hominem study (study 5), an error in the approval process meant that the preregistration was approved by all authors after data were already collected; however, the wording of the preregistration itself was not changed.

### Sample and procedure

For studies 1 to 6, we conducted an a priori power calculation to determine the minimum sample size needed to detect expected effects (including interaction effects), using GPower. In line with established standards in inoculation research, we used a power of 0.95 and α = 0.05, with an estimated predicted effect size of *d* = 0.20, with interactions (*49*). Doing so yields a target sample size of 542 participants per condition, for a total target sample of *N* = 1084 per study. In total, we recruited 6464 participants for studies 1 to 6: *n*_1_ = 1072 (study 1; emotional language), *n*_2_ = 1086 (study 2; incoherence), *n*_3_ = 1095 (study 3; false dichotomies), *n*_4_ = 1080 (study 4; scapegoating), *n*_5_ = 1083 (study 5; ad hominem), and *n*_6_ = 1068 (study 6; emotional language replication study). Each sample was nationally balanced on the U.S. population on age, gender, and ethnicity. See tables S1a and S1b for each study’s sample composition.

Each survey was administered through the survey platform Qualtrics. Participants were recruited via the online platform Prolific Academic and were paid GBP 1.50 in line with minimum wage requirements in the United Kingdom. This study was approved by the Cambridge Psychology Research Ethics Committee (PRE.2020.085).

For study 7, we ran a YouTube ad campaign with two of the inoculation videos among a random sample of YouTube users who met the following criteria: (i) 18 years or older, (ii) located in the United States, (iii) English speaking, and (iv) having recently watched at least one political or news video on YouTube. We optimized the campaigns for obtaining a target number of survey responses and not getting repeat views. We ran three separate campaigns over a period of 15 days. Over this period, the videos were shown to about 5.4 million YouTube users. A total of 967,347 people watched either the emotional language video (422,371 views) or the false dichotomies video (544,976 views) as a YouTube ad for 30 s or more; see table S56.

A random 30% of this group of users was also shown a single (voluntary) test question embedded within the YouTube platform within 24 hours of viewing the inoculation video, where they were asked to identify which manipulation technique is being used in a fictional social media post (four choice options, only one being correct; see fig. S8 for examples of how these items were implemented). The control group consisted of YouTube users who met the same selection criteria as the treatment group but were only shown a test question and not any of the inoculation videos. We ensured that there was no overlap between users for the different test questions. In total, 22,632 participants (11,432 treatment and 11,200 control) answered a test question. Participants were given the option to skip the ad if they wanted. The average cost per 30-s video view was $0.05. See tables S55 and S56 for more details.

### Outcome measures

The item test in studies 1 to 6 included four outcome measures for each study (*28*, *47*): technique recognition (the ability to discern whether a social media post makes use of a particular manipulation technique), confidence in spotting these manipulation techniques, trustworthiness discernment (the ability to discern trustworthy from untrustworthy content), and sharing discernment (a measure of the quality of people’s sharing decisions, i.e., the difference in the self-reported willingness to share manipulative and nonmanipulative content with others in one’s network).

“Discernment” (i.e., technique/trustworthiness/sharing discernment) is defined as the difference between the averaged neutral (nonmanipulative) post scores and manipulative post scores for each outcome measure (*6*, *32*, *47*), the exception being confidence, for which we present the results for the manipulative and neutral posts separately. The reason for this is that “confidence discernment” is not a meaningful analytical construct. For example, if participants become significantly more confident both in their assessment that a manipulative post is manipulative and a neutral post is not after an intervention, then confidence discernment would nonetheless be low and would therefore not be a good indicator of improved confidence in one’s ability to discern manipulative and nonmanipulative social media content.

Our stimuli (see the “Stimuli” section below) included the following measures (each with a seven-point response scale: 1 being “strongly disagree” and 7 being “strongly agree”) administered immediately after the intervention to both the treatment and control groups:

1) This post is manipulative/is incoherent/contains a false dichotomy/constitutes scapegoating/contains an ad hominem

2) I am confident in my assessment of this post’s manipulativeness/incoherence/of whether it contains a false dichotomy/whether it constitutes scapegoating/whether it contains an ad hominem

3) This post is trustworthy

4) I would share this post with people in my network

**Fig. 4.**
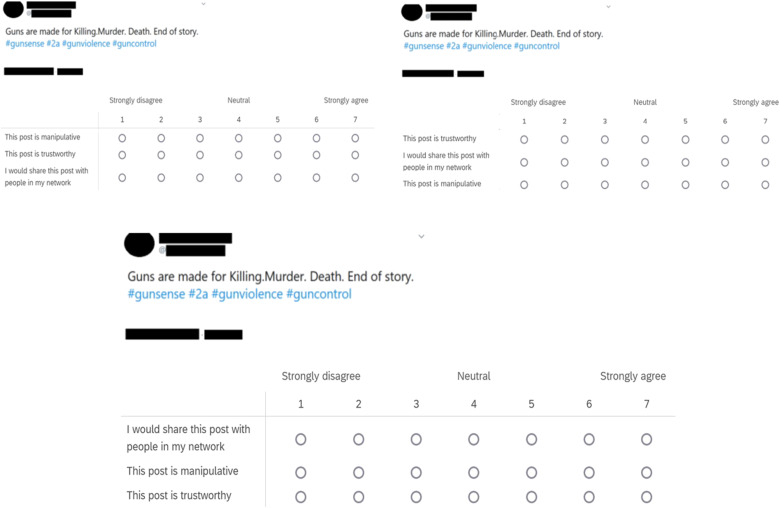
Examples of stimuli used in study 6. The three outcome measure response orders (MTS, TSM, and SMT).

For study 6, we varied the outcome measure response order across participants, as one of the study’s goals was to check whether the order in which participants respond to the measures beneath each of the stimuli has any bearing on the results (*29*, *30*). Participants were randomly assigned to one of three response orders (after being randomly assigned to an experimental condition): MTS (*n* = 364), TSM (*n* = 361), or SMT (*n* = 343). The same assigned response order was used for all stimuli. We excluded the confidence measure in study 6 because this measure is dependent on the “technique recognition” (manipulativeness) measure, and so varying the response order by assessing confidence before technique recognition would be confusing to the participant. See Fig. 4 for examples of the three response orders used in study 6.

In study 7, because of the limitations of the YouTube brand lift survey feature, we were only able to implement one test question per participant. In addition, YouTube brand lift surveys are limited because it is not possible to implement Likert-type questions. We therefore slightly modified the procedure from studies 1 to 6: Participants were shown a single sentence containing a manipulation technique (either emotional language or a false dichotomy; the stimuli themselves were taken from studies 1 and 3, with minor adaptations due to length limitations; see the “Stimuli” section below) and were asked to indicate what technique is used (four response options: one correct and three incorrect). For example, for the emotional language items, we asked participants whether a sentence contained (i) a command, (ii) emotional language, (iii) a false dichotomy, or (iv) none of these. As we were limited in the number of test questions that we could administer because of the cost of running the YouTube campaign (see table S56) and were primarily interested in participants’ ability to identify manipulation techniques, we only included test questions that contained a manipulation technique and no control/neutral items. Unlike studies 1 to 6, we therefore did not use discernment but rather the correct identification of the techniques as our outcome variable of interest. See fig. S8 for examples of how the test questions were implemented within the YouTube environment. See table S55 for descriptive statistics such as the number of responses per item, the number of correct responses, etc.

### Stimuli

**Fig. 5.**
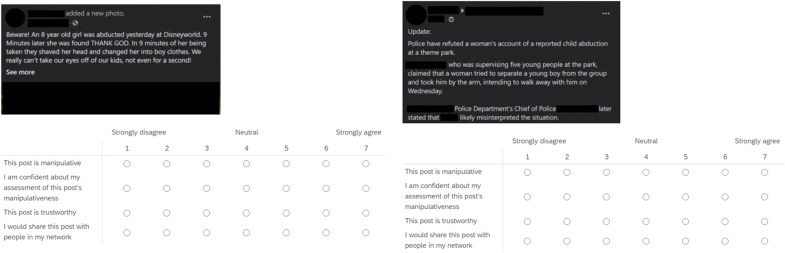
Examples of stimuli used in studies 1 to 6. Taken from studies 1 and 6, emotional language (*53*), one manipulative (left) and its neutral counterpart (right). See also fig. S7 for examples of stimuli used in each study.

For studies 1 to 6, we designed 20 test stimuli for each study in the form of fictitious social media posts (made to look like they were taken from Twitter or Facebook). All stimuli were reviewed by four experts and covered the use of manipulation techniques in contexts in which misinformation is common (such as climate change) but did not cover the same topics as the inoculation videos (see note x in the Supplementary Materials). Participants were shown a series of 10 stimuli, each of which was randomly either manipulative (i.e., containing the manipulation technique relevant to the study) or a neutral post matched to the manipulative post, similar in content and length but not containing the manipulation technique against which people in the treatment group were inoculated. On average, participants therefore saw five manipulative and five neutral posts (although different participants saw different specific stimuli). For both the manipulative and neutral posts, source information (profile picture, name, username, etc.) was blacked out to avoid biasing participants’ responses. All stimuli can be found in the “Stimuli” folder on OSF (https://osf.io/3769y/). Figure 5 shows examples of a manipulative post and its neutral counterpart, along with the four outcome measures. See fig. S7 for a more extensive overview of manipulative and neutral stimuli used in studies 1 to 6.

For studies 2 to 5 (incoherence, false dichotomies, scapegoating, and ad hominem), stimuli design was straightforward, as these techniques can be clearly defined and embedded in the stimuli. For the emotional language studies (studies 1 and 6), this is not the case, as the use of emotional language in social media content is not necessarily manipulative. We therefore conducted a stimulus validation test (using sentiment analysis) to confirm whether the manipulative stimuli capture the intended dimension of (manipulative) emotionality (and the neutral stimuli do not), for which we refer to Supplementary Analyses.

**Fig. 6.**
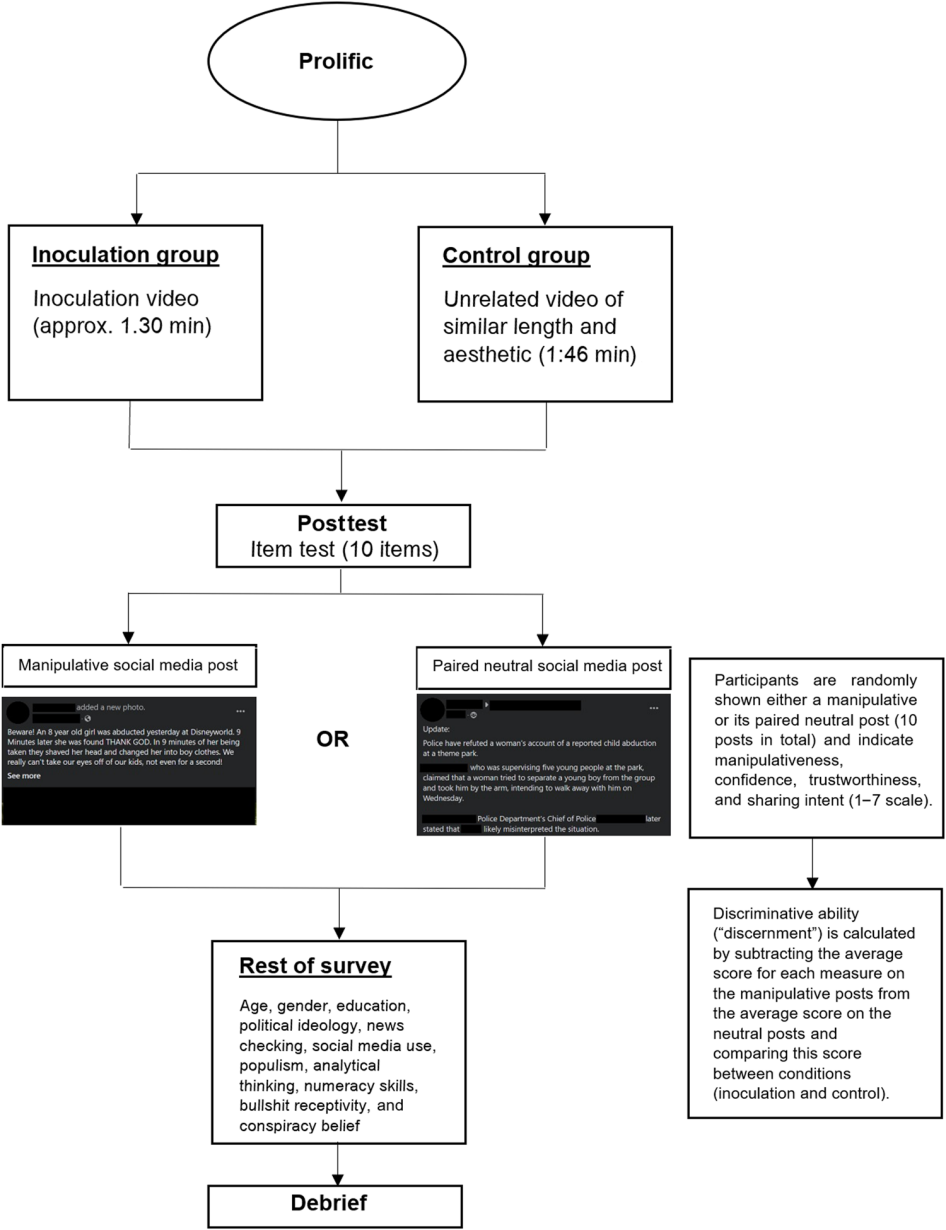
Studies 1 to 6 design flowchart.

Figure 6 shows the study design for studies 1 to 6 in more detail (study 6 differed from studies 1 to 5 in that confidence was not assessed in the item test, and we included different covariates; see below). In study 7, participants were asked to identify which manipulation technique was present in one of the following six stimuli, which were adapted from the stimuli used in studies 1 and 3 (see fig. S8 for examples of how these items were implemented on YouTube):

#### Emotional language

Item 1: “What this airline did for its passengers will make you tear up - SO heartwarming.” (see note ix in the Supplementary Materials)

Item 2: “Sick senior begged for help at hospital was given TERRIBLE medical care only after ENORMOUS wait.”

Item 3: “Horrific TV show inspiring kids to do dangerous stunts across the country!” (see note ix in the Supplementary Materials)

#### False dichotomies

Item 1: “We either need to improve our education system or deal with crime on the streets.”

Item 2: “Caffeine either keeps you awake for 4 hours or it has no effect at all.”

Item 3: “People are either part of the solution or they are part of the problem.”

### Covariates

Aside from the item test, participants in studies 1 to 6 were also asked a series of demographic and other questions. Alongside standard demographic variables (age group, gender, education, and political ideology; 1 being “very left-wing” and 7 being “very right-wing”), we also included the following measures as covariates, which research has shown are associated with susceptibility to misinformation:

1) Populist attitudes (*35*)

2) Analytical (or “intuitive” versus “reflective”) thinking, using the three-item CRT (*38*, *50*)

3) Numerical thinking, using the combined score on the three-item Schwartz test and one item from the risk assessment test by Wright *et al.* (*32*, *51*, *52*)

4) Bullshit receptivity (*36*)

5) Conspiracy mentality questionnaire, five items (*37*)

6) How often people check the news (1 being “never” and 5 being “all the time”) (*2*)

7) Social media use (1 being “never” and 5 being “all the time”) (*2*)

In study 6, instead of populism, analytical thinking, numerical thinking, bullshit receptivity, and conspiracy belief, we assessed the Ten-Item Personality Inventory (*39*), actively open-minded thinking (*33*, *40*), and the 20-item misinformation susceptibility test (*33*, *41*). See figs. S2 to S6 and tables S23 to S32, S38 to S48, and S52 to 54 for the full moderation analyses.

## Supplementary Materials

### This PDF file includes:

Notes i to x Supplementary Analyses Figs. S1 to S8 Tables S1 to S56 References
