# Supplementary material for: Psychological inoculation improves resilience against misinformation on social media
Source: Sci Adv. 2022 Aug 24;8(34):eabo6254. doi: 10.1126/sciadv.abo6254 (PMC9401631; doi:10.1126/sciadv.abo6254)
Supplement: Supplementary file 1 — Notes i to x Supplementary Analyses Figs. S1 to S8 Tables S1 to S56 References [file sciadv.abo6254_sm.pdf]

Science Advances
AAAS

Supplementary Materials for

Psychological inoculation improves resilience against misinformation on social media

Jon Roozenbeek *et al.*

Corresponding author: Jon Roozenbeek, jjr51@cam.ac.uk

Sci. Adv. **8**, eabo6254 (2022)
DOI: 10.1126/sciadv.abo6254

This PDF file includes:

Notes i to x
Supplementary Analyses
Figs. S1 to S8
Tables S1 to S56
References

Notes

[i] We deviate from our preregistrations for Studies 1-5 in several ways. First, we preregistered that we would conduct linear regressions to check if there are "three-way interactions" with each of the covariates. This was an error on the part of the authors, because there are only two interacting variables (condition and covariate), plus the outcome variable. Therefore, we instead check for *two-way* interactions between condition (treatment - control) and each of the covariates, predicting technique discernment (see Tables S44-S48 in the supplement); for additional clarity, we also provide linear regressions with all these covariates in the same model, for each of our four outcome variables, see Tables S23-S27. Second, we did not collect our preregistered covariates of minority status and region of origin, and so do not include these analyses in our manuscript.

[ii] We deviate from our preregistration for Study 7 in several ways. First, we preregistered that we would administer 5 survey items per study, but due to cost limitations could only administer 3. Second, we aimed to collect 4,000 survey responses per question for a total of 80,000 responses. As it proved to be more difficult than anticipated to collect these responses (most likely because the survey questions were shown at the very end of a YouTube video), we did not achieve our target sample; see Table S55. Third, we do not provide Bayesian analyses here due to space limitations. Fourth, we anticipated a Smallest Effect Size of Interest of Cohen's $d = 0.10$; we instead provide effect sizes in Cohen's h (see again Table S55), as this is the most applicable effect size for the two-proportion z-tests that we present here. Finally, we preregistered that we would conduct a series of moderation analyses, but we were not able to obtain any demographic data from YouTube, and so are unable to provide these analyses.

[iii] For the confidence measure, discernment is not a meaningful analytical construct, because a hypothetical participant who is both highly confident that a manipulative post contains a particular manipulation technique and that a non-manipulative post does *not* contain such a technique would nonetheless have low "confidence discernment".

[iv] In a further analysis (not preregistered), we checked if participants update their confidence in the right direction, i.e., if they only become more confident if they are also more accurate in their assessment. To do so, we conducted a series of linear regressions with technique recognition of manipulative and neutral stimuli as the dependent variables and the confidence measures and the condition (inoculation/control) as independent variables. For all five studies, we find that, for the manipulative stimuli, the perceived use of a technique is significantly and positively correlated with participants' confidence in recognizing these techniques, when controlling for experimental condition (all p-values < 0.001). For the neutral stimuli, we find that perceived technique use is significantly and *negatively* correlated with confidence (all p-values < 0.008), in the sense that participants who indicate that neutral stimuli are *not* manipulative ((i.e., they are more correct in their assessment) also tend to be highly confident in this assessment. We thus find that greater accuracy in manipulation technique recognition is consistently correlated with greater confidence, indicating that participants update their confidence for the right reason, and not due to overconfidence. See Tables S36 and S37.

[v] We also conducted a series of exploratory analyses of the sharing measure that excluded "never sharers", i.e., participants who answered "strongly disagree" on the sharing measure for all stimuli. The exclusion of participants who would never share either manipulative or neutral posts with people in their network follows guidelines established in previous work by Pennycook et al. (*54*); these results are highly similar to those reported below, and can be found in Table S35.

[vi] Technique recognition for control group participants is higher in some studies than others: an ANOVA shows that technique recognition among control group participants differs significantly across studies ($F(4,2787) = 84.1$, $p < 0.001$, $\eta^2 = 0.109$, $d = 0.70$), and is highest in the *scapegoating* study, for which we also report a descriptively lower effect size than for the other four studies (see Table S33). We find the same pattern for trustworthiness (see Table S34). Thus, it is possible that compared to the other manipulation techniques, participants were better at spotting scapegoating in social media content even without an intervention, which may explain the lower effect size and non-significant effects for the trustworthiness and sharing intentions measures.

[vii] In addition to the preregistered moderation analyses, we conducted a series of (non-preregistered) ANOVAs with technique recognition as the dependent variable and condition (inoculation – control) and political ideology (converted from a 7-point scale to "left", "moderate" and "right"), "bullshit receptivity" (converted to "high" and "low"), and analytical thinking (also converted to "high" and "low"), respectively, as independent variables, separately for each study. We find that technique recognition is significantly higher in the inoculation condition than in the control condition for those on the left and right, as well as for moderates (all p-values < 0.003), for participants with high and low "bullshit receptivity" (all p-values < 0.01), and for participants with high and low analytical thinking scores (all p-values < 0.015), with two exceptions: there is no difference between conditions among moderates for the "emotional language" study ($p = 0.685$), and for moderates and conservatives for the "scapegoating" study ($p = 0.148$ and $p = 0.652$, respectively). See Tables S23-S27, S38-S43, and S52-S54 for the regression tables, as well as Figures S2-S6.

[viii] This type of survey is called a YouTube "brand lift" survey. For more information about how this works, see: https://support.google.com/youtube/answer/4574026?hl=en.

[ix] We note that Item 1 for the *emotional language* survey ("What this airline did for its passengers will make you tear up - SO heart-warming.") is different from the other two headlines in that it does not make use of *negative* emotions; as the *emotional language* video specifically inoculates people against the use of negative emotions such as fear, anger, or outrage, it is possible that this discrepancy between the headline and the lessons learned in the video explains the lack of a significant effect for Item 1.

[x] As an example, one of the manipulative (incoherent) posts from the *incoherence* study reads: "*The 'scientific consensus' on global warming is a myth. Only a few scientists dare go against the grain. They are our heroes, and they should be celebrated*". This post is incoherent because it simultaneously asserts that climate change consensus does not exist and that there are only a few scientists who disagree with the consensus.[34] Its non-manipulative counterpart reads "*While there is an ongoing discussion about the exact level of agreement, approximately 97% of scientists*

agree that anthropogenic climate change is happening". For the *emotional language* study, we used real-world examples of emotionally manipulative social media content as stimuli, following Brady et al. (*22*), and conducted a stimuli validation test using a sentiment analysis library to ensure that the manipulative stimuli capture the intended dimension of emotionality and that the neutral stimuli do not; please see the Supplementary Analyses section for further details.

Supplementary Analyses

Robustness checks

As preregistered, we conducted a series of linear regressions with robust standard errors at the rating level for the technique recognition, trustworthiness and sharing measures, clustered on study participants and stimuli (manipulative vs neutral), following the approach laid out by Pennycook et al. (*54*) The regression tables can be found in Tables S28-S32. The results show that the findings reported in the main body are robust, except for one: trustworthiness discernment for the *incoherence* video is significant when conducting a Student's (p = 0.002) and Bayesian t-test (BF_{10} = 7.876, indicating strong support in favor of hypothesis **H3** (*55*)), but not when doing a linear regression at the rating level (p = 0.168).

In addition (although not preregistered), we provide Bayesian t-test results alongside the standard Student's t-tests for the averaged manipulative, neutral and discernment scores, as well as for all individual stimuli. In line with standard practices for reporting Bayesian statistics, we used a Cauchy prior, centered around 0, with a width parameter of 0.707, representing an 80% chance that the observed effect sizes are between -2 and 2, as recommended by van Doorn et al (*55*). Similarly, a Bayes $Factor_{10}$ (BF_{10}) lower than 3 is considered weak support for the directional hypothesis; between 3 and 5 is considered medium support, and > 5 strong support (*55*). These Bayesian analyses support the findings reported in the main body; see Supplementary Tables S3-S22.

Finally, because it is known that many people on social media never share news or other information, we conducted a series of exploratory analyses of the sharing measure that excluded "never sharers", i.e., participants who answered "strongly disagree" (1 out of 7) on the sharing measure for all stimuli. The exclusion of participants who would never share either manipulative

or neutral posts with people in their network follows guidelines established in previous work by Pennycook et al. (*54*). When these “never-sharers” are excluded, we find a significant difference in sharing discernment for the *incoherence* study ($M_{control}$ = 0.70 vs $M_{inoculation}$ = 0.87, M_{diff} = 0.17, $t(2,872) = 2.332$, $p = 0.02$, $d = 0.16$, 95%CI [0.025, 0.29]), but not for *scapegoating* ($M_{control}$ = 0.67 vs $M_{inoculation}$ = 0.82, M_{diff} = 0.15, $t(2,850) = 1.835$, $p = 0.067$, $d = 0.13$, 95%CI [-0.009, 0.26]). See Table S35.

Emotional language study – stimuli validation test

While the manipulation techniques tested in Studies 2-5 (*incoherence, false dichotomies, scapegoating* and *ad hominem attacks*) are straightforward and require no further elaboration, this is not the case for Studies 1 and 6 (*emotional language*) (*22*, *56*). For these studies, the manipulative social media posts were selected from real-world examples of social media content that seeks to evoke strong negative emotions such as fear or outrage in a manipulative manner (*27*, *46*, *50*). To establish whether social media posts that contain manipulative content indeed make more use of negative emotional language and fearmongering than their matched controls (which were formulated to be emotionally neutral), we conducted a sentiment analysis using Empath, a Python-based linguistic sentiment analyzer (*57*). Empath iterates over a corpus of text (in this case the social media posts), and assigns numerical values to a particular category of language use (e.g., “fear”, “anger”, or “emotional language”) based on whether words in the text appear in its dictionary under that category. This method allows us to compare the extent to which emotional language is used in the emotional-manipulative and neutral social media posts. Figure S1 shows the results for the categories “emotional language”, “negative emotion”, “fear’, “hate” and “suffering’.

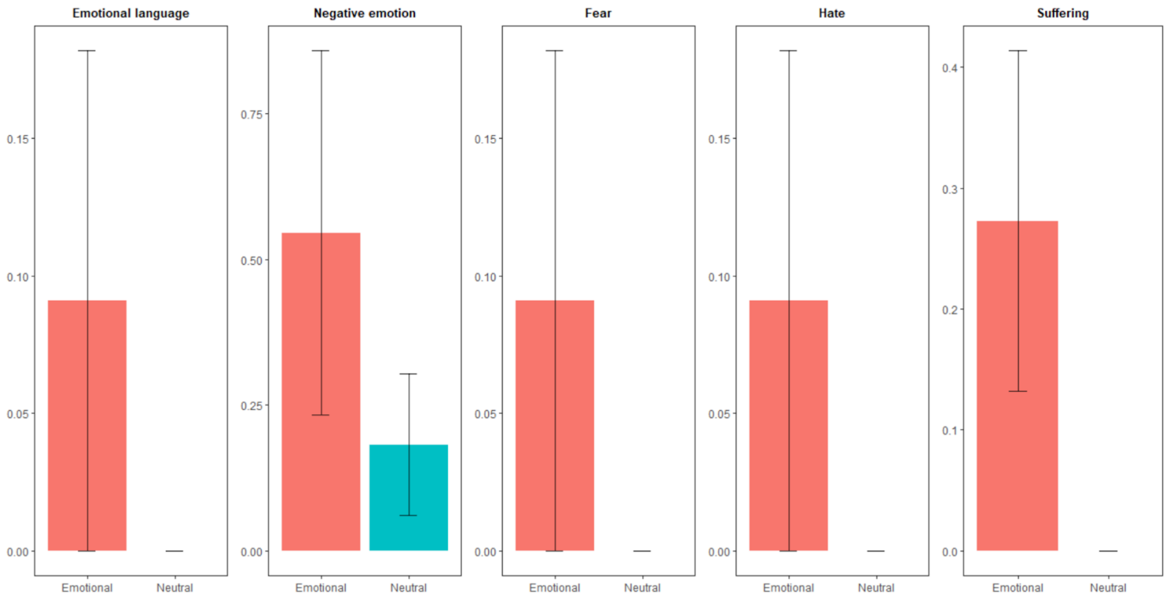

Fig. S1. Bar graphs for unnormalized Empath scores for the *emotional language*, *negative emotion*, *fear*, *hate* and *suffering* Empath categories, by stimulus type (manipulative vs neutral). The (unnormalized) Empath scores are indicated on the Y-axis of each bar graph. The red bars show the Empath scores for the emotional social media posts, the blue bars (where visible) show the Empath scores for the neutral posts. Error bars show the standard error.

Figure S1 shows that the emotional-manipulative social media posts use emotional language, negative emotion, fear-based language, hate-based language, and language related to suffering, whereas the non-emotional (neutral) posts contain no language related to these categories (except negative emotion). These results confirm that our test stimuli capture the intended dimension of (negative) emotionality.

Supplementary Figures

Figure S2.

Studies 1-5: technique recognition (Diff-Technique, or technique recognition), broken down by political ideology (where 1-3 on a 7-point scale is converted to “left”, 4 is “moderate” and 5-7 is “right”). For the full moderation analysis, see Tables S38 and S39. Figure created using Jamovi (www.jamovi.org).

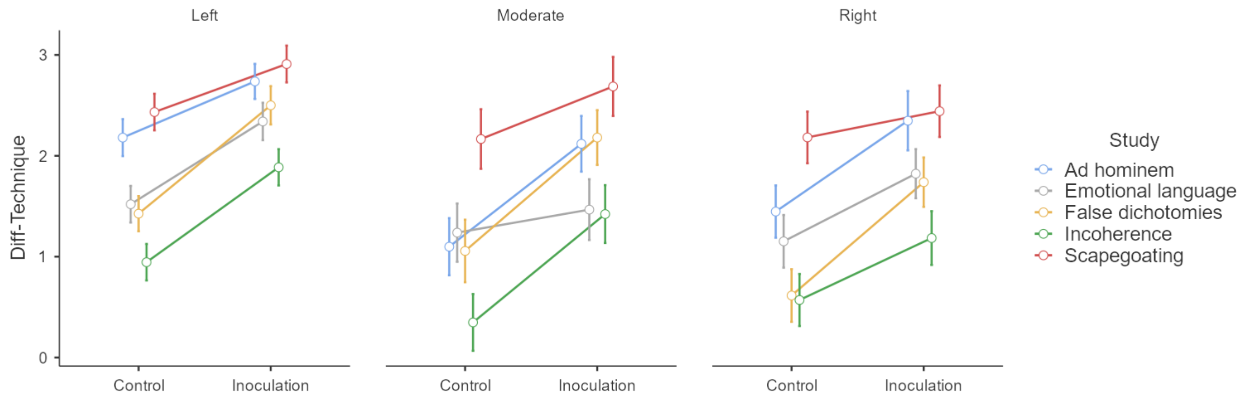

Figure S3.

Studies 1-5: technique recognition (Diff-Technique, or technique recognition), broken down by “bullshit receptivity” (where below-average “bullshit receptivity” scores are converted to “low” and above-average scores to “high”). For the full moderation analysis, see Tables S40 and S41. Figure created using Jamovi (www.jamovi.org).

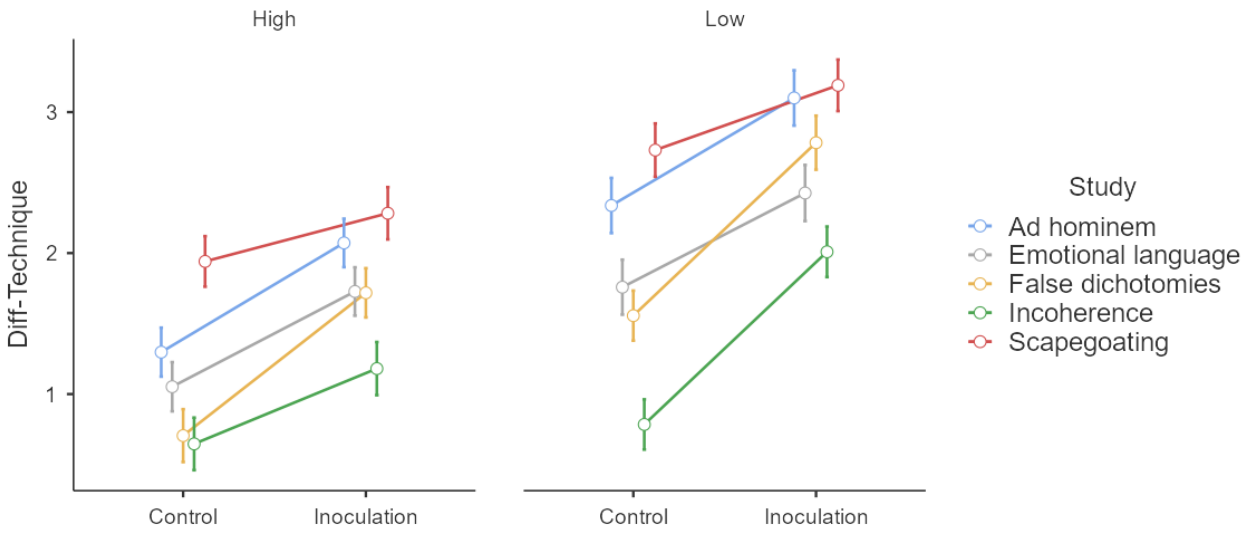

Figure S4.
Studies 1-5: technique recognition (Diff-Technique, or technique recognition), broken down by analytical thinking (where a score of 0 or 1 out of 3 is converted to "low" and a score of 2 or 3 out of 3 to "high"). For the full moderation analysis, see Tables S42 and S43. Figure created using Jamovi (www.jamovi.org).

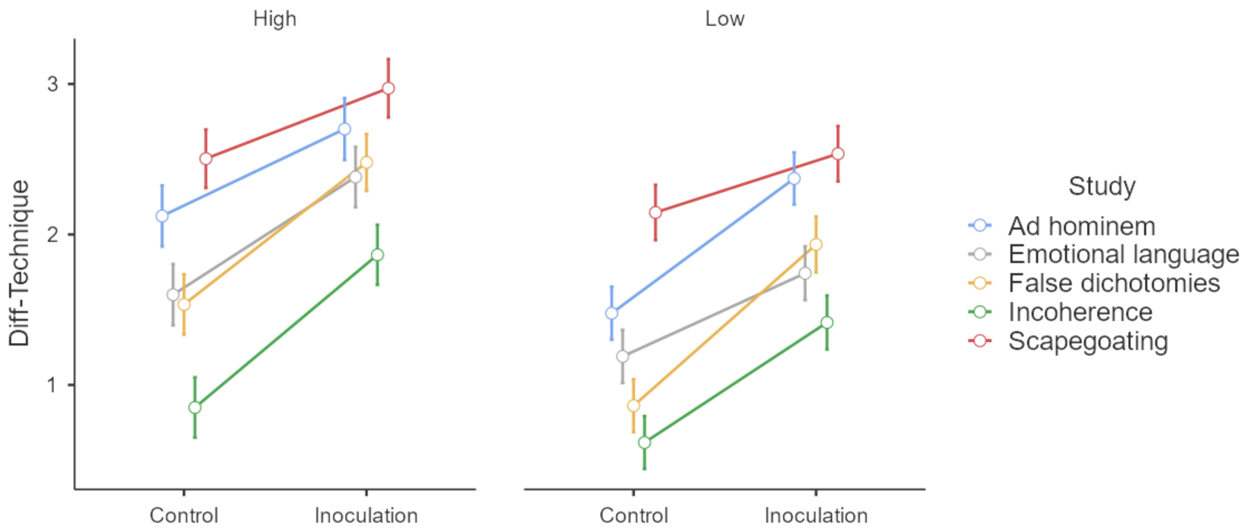

Figure S5.
Study 6: technique discernment, trustworthiness discernment, and sharing discernment, as moderated by actively open-minded thinking (AOT) (*40*). See also Table S53. Figure created using Jamovi (www.jamovi.org).

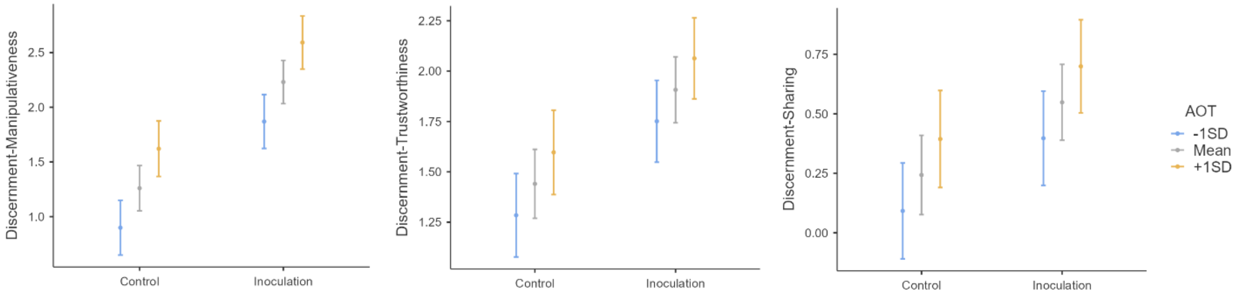

Figure S6.

Study 6: technique discernment, trustworthiness discernment, and sharing discernment, as moderated by Veracity Discernment Ability (the ability to discern true from false news headlines), according to the 20-item Misinformation Susceptibility Test (*41*). See also Table S54. Figure created using Jamovi (www.jamovi.org).

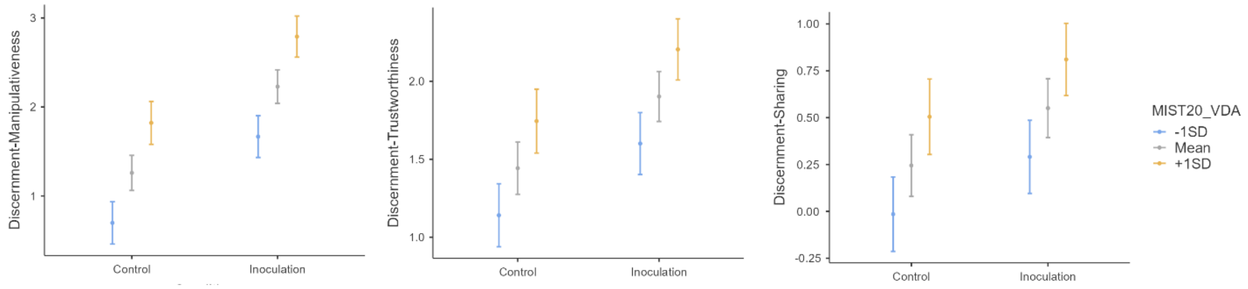

Figure S7.

Studies 1-6: Examples of manipulative and neutral stimuli.

Study	Manipulative	Neutral
1 & 6 Emotional language	[redacted] @[redacted] · [redacted]<br>NEWS ALERT: Baby formula linked to horrific outbreak of new, terrifying disease among helpless infants. Parents despair.<br>http://www.[redacted]<br>[redacted] @[redacted] · [redacted]<br>[redacted]	[redacted]<br>@[redacted]<br>Infants who receive formula feedings may be at slightly higher risk of acute otisis media (ear infections).<br>http://bit.ly/[redacted]<br>[redacted]
	[redacted] @[redacted] · [redacted]<br>"If we are silent, we lose our children": The moms fighting gun violence<br>[redacted]	[redacted]<br>@[redacted]<br>Why mothers are leading the fight for gun safety.<br>[redacted]
2 Incoherence	[redacted]<br>@[redacted]<br>@[redacted] You don't know what you're talking about. Even though science is yet to show a correlation, it's very clear that violent video games like [redacted] make people more likely to commit crimes.<br>[redacted]	[redacted]<br>@[redacted]<br>@[redacted] You don't know what you're talking about. Science is yet to show a correlation between violent video games like [redacted] and criminal behavior.<br>[redacted]
	[redacted]<br>@[redacted]<br>.@[redacted] Your beliefs leave me completely unfazed, but by God your vapid ideas make me angry.<br>[redacted]	[redacted]<br>@[redacted]<br>@[redacted] Your beliefs leave me completely unfazed. It's your face that makes me angry.<br>[redacted]
3 False dichotomies	[redacted] @[redacted] · [redacted]<br>Improving salaries for workers means businesses will go bankrupt. The choice is between small businesses and workers. It's simple mathematics.<br>[redacted]	[redacted] @[redacted] · [redacted]<br>Improving salaries for workers may affect businesses' bottom line, study says. What's less clear is how much small vs large businesses are impacted.<br>[redacted]
	[redacted]<br>Having trouble staying awake at work? Coffee only keeps you awake for 45 minutes, [redacted] energy lasts up to 4 hours. You know what to choose.<br>[redacted]	[redacted]<br>Having trouble staying awake at work? Drinking coffee keeps you awake for about 45 minutes, but certain healthy snacks can provide an even longer energy boost.<br>[redacted]
4 Scapegoating	[redacted] @[redacted] · [redacted]<br>@[redacted] Well, it's pretty obvious people of your ideology are incapable of being fiscally responsible. So yeah, the deficit and the fact that we can't pay for support for our vets IS your fault.	[redacted] @[redacted] · [redacted]<br>@[redacted] Your party takes pride in being the party of "fiscal responsibility" but doesn't have a lot to show for it. What about the deficit, and what do we do about veterans' support?

	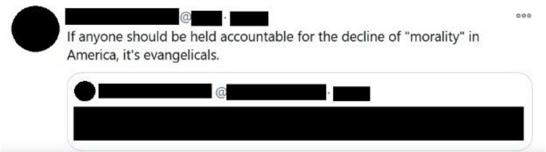	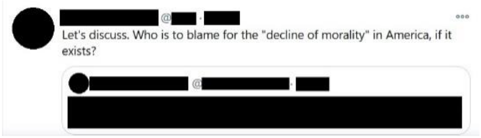
5 Ad hominem	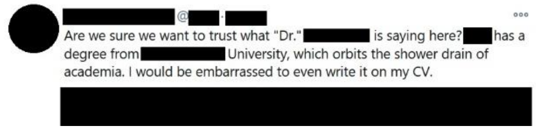	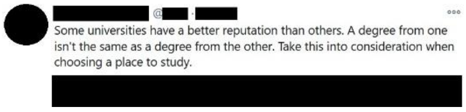
	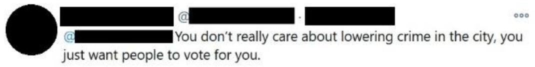	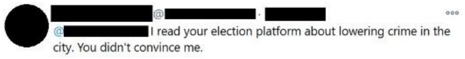

Figure S8.
Study 7: Examples of manipulative stimuli as they were implemented on YouTube (left: emotional language, right: false dichotomy).

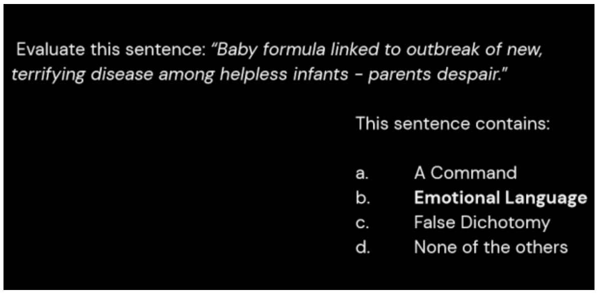

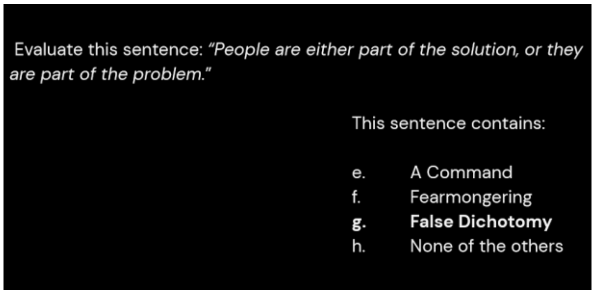

Supplementary Tables

Table S1a.

Sample composition for Studies 1-6 (demographics).

Variable	1 Emo. language		2 Incoherence		3 False dichotomies		4 Scapegoating		5 Ad hominem		6 Emo. language	
**Gender**	*N*	*%*	*N*	*%*	*N*	*%*	*N*	*%*	*N*	*%*	*N*	*%*
Female	547	51.0 %	542	49.9 %	556	50.8 %	539	49.9 %	538	49.7 %	540	50.7%
Male	509	47.5 %	531	48.9 %	531	48.5 %	525	48.6 %	528	48.8 %	510	47.8%
Non-binary	12	1.1 %	11	1.0 %	7	0.6 %	12	1.1 %	11	1.0 %	13	1.2%
Other	2	0.2 %	1	0.1 %	0	0	3	0.3 %	4	0.4 %	2	0.2%
Prefer not to say	2	0.2 %	1	0.1 %	1	0.1 %	1	0.1 %	2	0.2 %	1	0.1%
**Age**	*N*	*%*	*N*	*%*	*N*	*%*	*N*	*%*	*N*	*%*	*N*	*%*
18-24	163	15.2 %	128	11.8 %	106	9.7 %	132	12.2 %	172	15.9 %	114	10.8%
25-34	231	21.5 %	209	19.2 %	250	22.8 %	255	23.6 %	323	29.8 %	225	21.1%
35-44	195	18.2 %	192	17.7 %	178	16.3 %	212	19.6 %	244	22.5 %	190	17.8%
45-54	158	14.7 %	178	16.4 %	186	17.0 %	181	16.8 %	204	18.8 %	162	15.2%
55 or older	325	30.3 %	379	34.9 %	375	34.2 %	300	27.8 %	140	12.9 %	374	35.1%
**Education**	*N*	*%*	*N*	*%*	*N*	*%*	*N*	*%*	*N*	*%*	*N*	*%*
No formal education above age 16	14	1.3 %	6	0.6 %	6	0.5 %	7	0.6 %	4	0.4 %	8	0.8%
Professional or technical qualifications above age 16	28	2.6 %	33	3.0 %	31	2.8 %	30	2.8 %	26	2.4 %	25	2.3%
School education up to age 18	355	33.1 %	332	30.6 %	340	31.1 %	334	30.9 %	335	30.9 %	289	27.1%
Degree (Bachelor's) or equivalent	462	43.1 %	472	43.5 %	463	42.3 %	433	40.1 %	457	42.2 %	503	47.2%
Degree (Master's) or other postgraduate qualification	181	16.9 %	206	19.0 %	224	20.5 %	238	22.0 %	215	19.9 %	200	18.8%
Doctorate	32	3.0 %	37	3.4 %	31	2.8 %	38	3.5 %	46	4.2 %	41	3.8%
**How often do you check the news**	*N*	*%*	*N*	*%*	*N*	*%*	*N*	*%*	*N*	*%*	*N*	*%*
Never	9	0.8 %	14	1.3 %	12	1.1 %	4	0.4 %	13	1.2 %	11	1.0%
Rarely	85	7.9 %	67	6.2 %	80	7.3 %	58	5.4 %	91	8.4 %	94	8.8%
Sometimes	261	24.3 %	240	22.1 %	269	24.6 %	262	24.3 %	281	25.9 %	264	24.8%
Frequently	508	47.4 %	527	48.5 %	492	44.9 %	511	47.3 %	477	44.0 %	474	44.5%
All the time	209	19.5 %	238	21.9 %	242	22.1 %	245	22.7 %	221	20.4 %	222	20.8%
**How often do you use social media**	*N*	*%*	*N*	*%*	*N*	*%*	*N*	*%*	*N*	*%*	*N*	*%*
Never	33	3.1 %	43	4.0 %	39	3.6 %	42	3.9 %	21	1.9 %	41	3.8%
Rarely	66	6.2 %	71	6.5 %	88	8.0 %	85	7.9 %	87	8.0 %	101	9.5%
Sometimes	190	17.7 %	238	21.9 %	221	20.2 %	219	20.3 %	182	16.8 %	191	17.9%
Frequently	428	40.0 %	435	40.1 %	454	41.5 %	416	38.5 %	429	39.6 %	420	39.4%
All the time	354	33.1 %	299	27.5 %	293	26.8 %	318	29.4 %	364	33.6 %	312	29.3%
**Political party support**											*N*	*%*
Democratic Party											525	49.3%
Republican Party											195	18.3%
Independent											317	29.8%
Other											28	2.6%

Table S1b.
Sample composition for Studies 1-6 (covariates/continuous variables).

**Variable**	**1 Emo. lang**		**2 Incoherence**		**3 False dichot.**		**4 Scapegoating**		**5 Ad hominem**		**6 Emo. lang.**	
	Mean	*SD*	*Mean*	*SD*	*Mean*	*SD*	*Mean*	*SD*	*Mean*	*SD*	*Mean*	*SD*
Political ideology (1-7)	3.37	1.79	3.35	1.74	3.38	1.77	3.32	1.77	3.25	1.71	3.26	1.73
Populism (1-5)	3.78	0.61	3.68	0.61	3.72	0.61	3.71	0.60	3.79	0.59		
Bullshit receptivity (1-5)	2.73	0.95	2.58	0.92	2.67	0.95	2.70	0.94	2.77	0.92		
Conspiracy belief (0-1)	0.63	0.21	0.59	0.23	0.59	0.23	0.60	0.22	0.62	0.21		
Analytical thinking (0-3)	1.32	1.23	1.14	0.97	1.19	0.97	1.19	0.98	1.12	0.96		
Numerical thinking (0-4)	2.96	1.04	2.99	0.99	3.03	0.97	2.99	0.99	3.07	1.01		
Act. open-minded thinking (0-10)											4.17	0.54
MIST veracity discernment (0-1)											0.83	0.15
Personality (TIPI) (1-7)												
Openness											5.21	1.23
Conscientiousness											5.64	1.22
Extraversion											3.49	1.69
Agreeableness											5.40	1.23
Neuroticism (emo. stability)											4.88	1.61

Table S2.

Pilot study (emotional language): independent samples and Bayesian *t*-tests for averaged scores for manipulativeness, confidence (manipulative/fake posts and control/neutral posts separately), credibility, and sharing. *Note:* Bayesian prior used is 0.707 (representing an 80% chance that the effect size is between -2 and 2).

**Variable**		**Statistic**	**±%**	**df**	***p***	**M_{diff}**	**95% CI**	**Cohen's *d***	**95% CI**
Discernment-Manipulativeness	Student's t	-2.341		192	0.02	-0.48	-0.88, -0.075	-0.34	-0.62, -0.05
	Bayes $factor_{10}$	1.974	1.59E-08						
Fake-Confidence	Student's t	-2.075		192	0.039	-0.33	-0.65, -0.02	-0.30	-0.58, -0.01
	Bayes $factor_{10}$	1.151	2.30E-08						
Control-Confidence	Student's t	-0.161		192	0.872	-0.028	-0.37, 0.31	-0.02	-0.31, 0.26
	Bayes $factor_{10}$	0.158	5.84E-08						
Discernment-Credibility	Student's t	-0.0169		192	0.987	-0.0029	-0.34, 0.33	0.00	-0.28, 0.28
	Bayes $factor_{10}$	0.156	5.86E-08						
Discernment-Sharing	Student's t	-1.358		192	0.176	-0.19	-0.46, 0.08	-0.20	-0.48, 0.09
	Bayes $factor_{10}$	0.369	4.33E-08						

Table S3.

Study 1 (emotional language): technique recognition measure item-level results (independent samples and Bayesian *t*-tests). *Note:* Bayesian prior used is 0.707 (representing an 80% chance that the effect size is between -2 and 2). "-C-" denotes a manipulative post's matched neutral control.

**Manipulativeness**		**Statistic**	**±%**	**df**	***p***	**M_{diff}**	**95%CI**	**Cohen's *d***	**95%CI**
Manipulative-Manipulativeness	Student's t	9.2897		1069	< .001	0.68	0.53, 0.82	0.57	0.44, 0.69
	BF_{10}	4.59E+16	3.30E-22						
Neutral-Manipulativeness	Student's t	-0.2205		1069	0.825	-0.02	-0.16, 0.13	-0.01	-0.13, 0.11
	BF_{10}	0.0701	4.39e0-4						
Discernment-Manipulativeness	Student's t	7.9421		1068	< .001	0.69	0.52, 0.86	0.49	0.36, 0.61
	BF_{10}	9.82E+11	1.81E-17						
Abduction-Manipulativeness	Student's t	1.8014		527	0.072	0.30	-0.03, 0.63	0.16	-0.01, 0.33
	BF_{10}	0.4676	7.99e0-7						
Abduction-C-Manipulativeness	Student's t	-1.8126		541	0.07	-0.25	-0.53, 0.02	-0.16	-0.32, 0.01
	BF_{10}	0.4713	1.03e0-6						
Airline-Manipulativeness	Student's t	8.0943		531	< .001	1.23	0.93, 1.52	0.70	0.52, 0.88
	BF_{10}	1.37E+12	7.00E-20						
Airline-C-Manipulativeness	Student's t	0.8805		537	0.379	0.14	-0.17, 0.44	0.08	-0.09, 0.24
	BF_{10}	0.1398	3.43e0-6						
Gun2a-Manipulativeness	Student's t	4.033		533	< .001	0.60	0.31, 0.89	0.35	0.18, 0.52
	BF_{10}	239.5645	1.25e0-9						
Gun2a-C-Manipulativeness	Student's t	-1.8492		535	0.065	-0.28	-0.57, 0.02	-0.16	-0.33, 0.01
	BF_{10}	0.5066	8.18e0-7						
Immigration-Manipulativeness	Student's t	-1.4509		534	0.147	-0.23	-0.53, 0.08	-0.13	-0.30, 0.04
	BF_{10}	0.2678	1.61e0-6						
Immigr-C-Manipulativeness	Student's t	0.5145		534	0.607	0.08	-0.22, 0.37	0.04	-0.13, 0.21
	BF_{10}	0.1097	3.99e0-6						
Suicide-Manipulativeness	Student's t	7.7556		531	< .001	1.02	0.76, 1.28	0.67	0.49, 0.85
	BF_{10}	1.29E+11	8.53E-19						
Suicide-C-Manipulativeness	Student's t	0.0305		537	0.976	0.00	-0.30, 0.30	0.00	-0.17, 0.17
	BF_{10}	0.0959	5.12e0-6						
Baby-Manipulativeness	Student's t	5.1962		533	< .001	0.70	0.43, 0.96	0.45	0.28, 0.62
	BF_{10}	37385.6246	6.49E-12						
Baby-C-Manipulativeness	Student's t	-3.1298		535	0.002	-0.46	-0.76, -0.17	-0.27	-0.44, -0.10
	BF_{10}	10.9215	3.42e0-8						
Elephant-Manipulativeness	Student's t	9.2423		537	< .001	1.42	1.12, 1.72	0.80	0.61, 0.98
	BF_{10}	7.60E+15	1.02E-23						
Elephant-C-Manipulativeness	Student's t	2.3871		531	0.017	0.35	0.06, 0.64	0.21	0.04, 0.38
	BF_{10}	1.5275	2.42e0-7						
Health-Manipulativeness	Student's t	5.8033		531	< .001	0.84	0.55, 1.12	0.50	0.33, 0.68
	BF_{10}	827141.6117	2.40E-13						
Health-C-Manipulativeness	Student's t	-0.5035		537	0.615	-0.08	-0.38, 0.22	-0.04	-0.21, 0.13
	BF_{10}	0.1086	4.42e0-6						
Phone-Manipulativeness	Student's t	2.4336		532	0.015	0.40	0.08, 0.73	0.21	0.04, 0.38
	BF_{10}	1.6991	2.26e0-7						
Phone-C-Manipulativeness	Student's t	1.4221		536	0.156	0.22	-0.09, 0.53	0.12	-0.05, 0.29
	BF_{10}	0.2565	1.80e0-6						
Suppl-Manipulativeness	Student's t	3.8452		532	< .001	0.48	0.23, 0.73	0.33	0.16, 0.51
	BF_{10}	118.7088	2.50e0-9						
Suppl-C-Manipulativeness	Student's t	1.0095		536	0.313	0.16	-0.15, 0.46	0.09	-0.08, 0.26
	BF_{10}	0.1576	2.96e0-6						

[a] Levene's test is significant (p < .05), suggesting a violation of the assumption of equal variances

Table S4.

Study 1 (emotional language): confidence measure item-level results (independent samples and Bayesian *t*-tests). *Note:* Bayesian prior used is 0.707 (representing an 80% chance that the effect size is between -2 and 2). “-C-“ denotes a manipulative post’s matched neutral control.

Confidence		Statistic	±%	df	*p*	M_{diff}	95%CI	Cohen's *d*	95%CI
Manipulative-Confidence	Student's t	8.1402		1069	< .001	0.53	0.40, 0.66	0.50	0.37, 0.62
	BF_{10}	4.35E+12	4.04E-18						
Neutral-Confidence	Student's t	3.3833		1069	< .001	0.22	0.09, 0.35	0.21	0.09, 0.33
	BF_{10}	18.8684	1.46e0-6						
Discernment-Confidence	Student's t	5.583		1068	< .001	0.30	0.20, 0.41	0.34	0.22, 0.46
	BF_{10}	266058.84	8.59E-11						
Abduction-Confidence	Student's t	2.0473		527	0.041	0.28	0.01, 0.56	0.18	0.01, 0.35
	BF_{10}	0.7393	4.92e0-7						
Abduction-C-Confidence	Student's t	2.4558		541	0.014	0.30	0.06, 0.54	0.21	0.04, 0.38
	BF_{10}	1.7779	2.57e0-7						
Airline-Confidence	Student's t	5.6973		531	< .001	0.67	0.44, 0.91	0.49	0.32, 0.67
	BF_{10}	471090.10	4.36E-13						
Airline-C-Confidence	Student's t	1.5429		537	0.123	0.20	-0.05, 0.45	0.13	-0.04, 0.30
	BF_{10}	0.305	1.51e0-6						
Gun2a-Confidence	Student's t	3.9849		533	< .001	0.48	0.24, 0.71	0.34	0.17, 0.52
	BF_{10}	199.4475	1.52e0-9						
Gun2a-C-Confidence	Student's t	0.9657		535	0.335	0.12	-0.12, 0.36	0.08	-0.09, 0.25
	BF_{10}	0.1515	2.92e0-6						
Immigration-Confidence	Student's t	0.7445		534	0.457	0.09	-0.15, 0.33	0.06	-0.11, 0.23
	BF_{10}	0.126	3.56e0-6						
Immigr-C-Confidence	Student's t	0.0555		534	0.956	0.01	-0.24, 0.26	0.00	-0.17, 0.18
	BF_{10}	0.0966	4.56e0-6						
Suicide-Confidence	Student's t	7.7141		531	< .001	0.92	0.69, 1.16	0.67	0.49, 0.85
	BF_{10}	9.70E+10	1.15E-18						
Suicide-C-Confidence	Student's t	1.7828		537	0.075	0.22	-0.02, 0.46	0.15	-0.02, 0.32
	BF_{10}	0.4491	1.01e0-6						
Baby-Confidence	Student's t	5.7898		533	< .001	0.69	0.46, 0.93	0.50	0.33, 0.68
	BF_{10}	770621.43	2.74E-13						
Baby-C-Confidence	Student's t	2.473		535	0.014	0.32	0.07, 0.57	0.21	0.04, 0.38
	BF_{10}	1.8607	2.19e0-7						
Elephant-Confidence	Student's t	6.3991		537	< .001	0.78	0.54, 1.03	0.55	0.38, 0.73
	BF_{10}	2.34e0+7	8.77E-15						
Elephant-C-Confidence	Student's t	1.7567		531	0.08	0.24	-0.03, 0.50	0.15	-0.02, 0.32
	BF_{10}	0.432	9.18e0-7						
Health-Confidence	Student's t	4.6828		531	< .001	0.59	0.34, 0.84	0.41	0.23, 0.58
	BF_{10}	3477.1565	7.39E-11						
Health-C-Confidence	Student's t	3.4974		537	< .001	0.42	0.18, 0.65	0.30	0.13, 0.47
	BF_{10}	35.0431	1.02e0-8						
Phone-Confidence	Student's t	2.2503		532	0.025	0.30	0.04, 0.56	0.19	0.02, 0.37
	BF_{10}	1.1226	3.50e0-7						
Phone-C-Confidence	Student's t	1.6813		536	0.093	0.21	-0.04, 0.45	0.14	-0.02, 0.31
	BF_{10}	0.3789	1.19e0-6						
Suppl-Confidence	Student's t	3.9763		532	< .001	0.47	0.24, 0.70	0.34	0.17, 0.52
	BF_{10}	193.289	1.50e0-9						
Suppl-C-Confidence	Student's t	2.3224		536	0.021	0.29	0.04, 0.54	0.20	0.03, 0.37
	BF_{10}	1.3128	3.18e0-7						

[a] Levene's test is significant (p < .05), suggesting a violation of the assumption of equal variances

Table S5.

Study 1 (emotional language): trustworthiness measure item-level results (independent samples and Bayesian *t*-tests). *Note:* Bayesian prior used is 0.707 (representing an 80% chance that the effect size is between -2 and 2). “-C-“ denotes a manipulative post’s matched neutral control.

**Trustworthiness**		**Statistic**	**±%**	**df**	***p***	**M_{diff}**	**95%CI**	**Cohen's *d***	**95%CI**
Manipulative-Trustworthiness	Student's t	-0.474		1069	0.636	-0.03	-0.17, 0.10	-0.03	-0.15, 0.09
	BF_{10}	0.0765	4.01E-04				0.00, 0.00		
Neutral-Trustworthiness	Student's t	4.156		1069	< .001	0.26	0.14, 0.38	0.25	0.13, 0.37
	BF_{10}	321.9585	8.10E-08				0.00, 0.00		
Discernment-Trustworthiness	Student's t	4.051		1068	< .001	0.29	0.15, 0.43	0.25	0.13, 0.37
	BF_{10}	212.0718	1.23E-07				0.00, 0.00		
Abduction-Trustworthiness	Student's t	2		527	0.046	0.31	0.01, 0.61	0.17	0.00, 0.34
	BF_{10}	0.674	5.43E-07				0.00, 0.00		
Abduction-C-Trustworthiness	Student's t	2.705		541	0.007	0.33	0.09, 0.57	0.23	0.06, 0.40
	BF_{10}	3.3061	1.34E-07				0.00, 0.00		
Airline-Trustworthiness	Student's t	-2.014		531	0.045	-0.26	-0.52, -0.01	-0.17	-0.34, 0.00
	BF_{10}	0.6896	5.77E-07				0.00, 0.00		
Airline-C-Trustworthiness	Student's t	1.649		537	0.1	0.21	-0.04, 0.45	0.14	-0.03, 0.31
	BF_{10}	0.3593	1.27E-06				0.00, 0.00		
Gun2a-Trustworthiness	Student's t	-1.765		533	0.078	-0.27	-0.57, 0.03	-0.15	-0.32, 0.02
	BF_{10}	0.4369	9.50E-07				0.00, 0.00		
Gun2a-C-Trustworthiness	Student's t	0.853		535	0.394	0.11	-0.14, 0.35	0.07	-0.10, 0.24
	BF_{10}	0.1371	3.25E-06				0.00, 0.00		
Immigration-Trustworthiness	Student's t	2.119		534	0.035	0.33	0.02, 0.64	0.18	0.01, 0.35
	BF_{10}	0.8505	4.75E-07				0.00, 0.00		
Immigr-C-Trustworthiness	Student's t	1.559		534	0.12	0.20	-0.05, 0.45	0.14	-0.04, 0.31
	BF_{10}	0.314	1.31E-06				0.00, 0.00		
Suicide-Trustworthiness	Student's t	-1.268		531	0.205	-0.17	-0.44 0.10	-0.11	-0.28, 0.06
	BF_{10}	0.2107	2.02E-06				0.00, 0.00		
Suicide-C-Trustworthiness	Student's t	1.429		537	0.154	0.19	-0.07, 0.44	0.12	-0.05, 0.29
	BF_{10}	0.2589	1.80E-06				0.00, 0.00		
Baby-Trustworthiness	Student's t	-0.868		533	0.386	-0.13	-0.41, 0.16	-0.08	-0.24, 0.09
	BF_{10}	0.1389	3.24E-06				0.00, 0.00		
Baby-C-Trustworthiness	Student's t	4.068		535	< .001	0.53	0.27, 0.78	0.35	0.18, 0.52
	BF_{10}	273.339	1.17E-09				0.00, 0.00		
Elephant-Trustworthiness	Student's t	-2.431		537	0.015	-0.33	-0.60, -0.06	-0.21	-0.38, -0.04
	BF_{10}	1.6821	2.51E-07				0.00, 0.00		
Elephant-C-Trustworthiness	Student's t	1.938		531	0.053	0.25	0.00, 0.51	0.17	0.00, 0.34
	BF_{10}	0.598	6.51E-07				0.00, 0.00		
Health-Trustworthiness	Student's t	-1.699		531	0.09	-0.23	-0.49, 0.04	-0.15	-0.32, 0.02
	BF_{10}	0.3919	1.04E-06				0.00, 0.00		
Health-C-Trustworthiness	Student's t	4.273		537	< .001	0.53	0.28, 0.77	0.37	0.20, 0.54
	BF_{10}	614.1642	5.12E-10				0.00, 0.00		
Phone-Trustworthiness	Student's t	0.742		532	0.459	0.11	-0.17, 0.39	0.06	-0.11, 0.23
	BF_{10}	0.1259	3.52E-06				0.00, 0.00		
Phone-C-Trustworthiness	Student's t	1.545		536	0.123	0.20	-0.05, 0.45	0.13	-0.04, 0.30
	BF_{10}	0.3062	1.49E-06				0.00, 0.00		
Suppl-Trustworthiness	Student's t	0.158		532	0.875	0.02	-0.25, 0.29	0.01	-0.16, 0.18
	BF_{10}	0.0976	4.46E-06				0.00, 0.00		
Suppl-C-Trustworthiness	Student's t	1.321		536	0.187	0.18	-0.09, 0.45	0.11	-0.06, 0.28
	BF_{10}	0.2244	2.04E-06						

[a] Levene's test is significant ($p < .05$), suggesting a violation of the assumption of equal variances

Table S6.

Study 1 (emotional language): sharing intentions measure item-level results (independent samples and Bayesian *t*-tests). *Note:* Bayesian prior used is 0.707 (representing an 80% chance that the effect size is between -2 and 2). "-C-" denotes a manipulative post's matched neutral control.

Sharing		Statistic	±%	df	*p*	M_{diff}	95% CI	Cohen's *d*	95%CI
Manipulative-Sharing	Student's t	-0.24		1069	0.81	-0.02	-0.20, 0.15	-0.01	-0.13, 0.11
	BF_{10}	0.0704	4.37E-04						
Neutral-Sharing	Student's t	2.209		1069	0.027	0.21	0.02, 0.39	0.14	0.01, 0.26
	BF_{10}	0.7568	3.87E-05						
Discernment-Sharing	Student's t	3.496		1068	< .001	0.23	0.10, 0.36	0.21	0.09, 0.33
	BF_{10}	27.5131	9.83E-07						
Abduction-Sharing	Student's t	2.742		527	0.006	0.47	0.13, 0.81	0.24	0.07, 0.41
	BF_{10}	3.683	9.01E-08						
Abduction-C-Sharing	Student's t	-0.4		541	0.689	-0.07	-0.39, 0.26	-0.03	-0.20, 0.13
	BF_{10}	0.1032	5.09E-06						
Airline-Sharing	Student's t	-1.748		531	0.081	-0.28	-0.59, 0.03	-0.15	-0.32, 0.02
	BF_{10}	0.4249	9.62E-07						
Airline-C-Sharing	Student's t	-0.259		537	0.796	-0.04	-0.37, 0.28	-0.02	-0.19, 0.15
	BF_{10}	0.0991	4.94E-06						
Gun2a-Sharing	Student's t	-0.151		533	0.88	-0.02	-0.33, 0.28	-0.01	-0.18, 0.16
	BF_{10}	0.0974	4.64E-06						
Gun2a-C-Sharing	Student's t	0.6		535	0.549	0.10	-0.23, 0.43	0.05	-0.12, 0.22
	BF_{10}	0.1147	3.93E-06						
Immigration-Sharing	Student's t	0.19		534	0.85	0.03	-0.30, 0.37	0.02	-0.15, 0.19
	BF_{10}	0.0979	4.65E-06						
Immigr-C-Sharing	Student's t	1.586		534	0.113	0.25	-0.06, 0.57	0.14	-0.03, 0.31
	BF_{10}	0.3271	1.26E-06						
Suicide-Sharing	Student's t	-0.279		531	0.78	-0.04	-0.34, 0.26	-0.02	-0.19, 0.15
	BF_{10}	0.1001	4.44E-06						
Suicide-C-Sharing	Student's t	-0.267		537	0.79	-0.04	-0.33, 0.25	-0.02	-0.19, 0.15
	BF_{10}	0.0993	4.94E-06						
Baby-Sharing	Student's t	-0.588		533	0.557	-0.09	-0.41, 0.22	-0.05	-0.22, 0.12
	BF_{10}	0.1138	3.99E-06						
Baby-C-Sharing	Student's t	3.26		535	0.001	0.53	0.21, 0.85	0.28	0.11, 0.45
	BF_{10}	16.2521	2.25E-08						
Elephant-Sharing	Student's t	-1.297		537	0.195	-0.21	-0.52, 0.11	-0.11	-0.28, 0.06
	BF_{10}	0.2172	2.16E-06						
Elephant-C-Sharing	Student's t	1.42		531	0.156	0.24	-0.09, 0.58	0.12	-0.05, 0.29
	BF_{10}	0.2571	1.59E-06						
Health-Sharing	Student's t	-1.088		531	0.277	-0.17	-0.47, 0.13	-0.09	-0.26, 0.08
	BF_{10}	0.1714	2.50E-06						
Health-C-Sharing	Student's t	2.41		537	0.016	0.39	0.07, 0.70	0.21	0.04, 0.38
	BF_{10}	1.6024	2.60E-07						
Phone-Sharing	Student's t	0.8		532	0.424	0.14	-0.20, 0.49	0.07	-0.10, 0.24
	BF_{10}	0.1315	3.36E-06						
Phone-C-Sharing	Student's t	1.447		536	0.149	0.25	-0.09, 0.58	0.12	-0.04, 0.29
	BF_{10}	0.2654	1.73E-06						
Suppl-Sharing	Student's t	0.729		532	0.466	0.11	-0.19, 0.41	0.06	-0.11, 0.23
	BF_{10}	0.125	3.44E-06						
Suppl-C-Sharing	Student's t	1.145		536	0.253	0.17	-0.12, 0.47	0.10	-0.07, 0.27
	BF_{10}	0.1816	2.55E-06						

[a] Levene's test is significant ($p < .05$), suggesting a violation of the assumption of equal variances

Table S7.

Study 2 (incoherence): technique recognition measure item-level results (independent samples and Bayesian *t*-tests). *Note:* Bayesian prior used is 0.707 (representing an 80% chance that the effect size is between -2 and 2). "-C-" denotes a manipulative post's matched neutral control.

Incoherence		Statistic	±%	df	*p*	M_{diff}	95%CI	Cohen's *d*	95%CI
Manipulative-Incoherence	Student's t	17.89		1084	< .001	1.44	1.28, 1.60	1.09	0.95, 1.22
	BF_{10}	1.82E+59	1.63E-65						
Neutral-Incoherence	Student's t	7.08		1082	< .001	0.52	0.38, 0.67	0.43	0.31, 0.55
	BF_{10}	2.21e0+9	1.04E-14						
Discernment-Incoherence	Student's t	10.12		1082	< .001	0.92	0.74, 1.10	0.62	0.49, 0.74
	BF_{10}	7.57E+19	2.13E-25						
StockMarket-Incoherence	Student's t	11.13		543	< .001	1.81	1.49, 2.13	0.95	0.77, 1.14
	BF_{10}	6.33E+22	5.29E-31						
StockMarket-C-Incoherence	Student's t	7.84		539	< .001	1.34	1.00, 1.67	0.68	0.50, 0.85
	BF_{10}	2.28E+11	5.90E-19						
Morality-Incoherence	Student's t	8.72		543	< .001	1.45	1.12, 1.77	0.75	0.57, 0.93
	BF_{10}	1.45E+14	8.99E-22						
Morality-C-Incoherence	Student's t	7.29		539	< .001	1.23	0.90, 1.56	0.63	0.45, 0.80
	BF_{10}	5.78e0+9	3.03E-17						
TanningBeds-Incoherence	Student's t	3.93		543	< .001	0.70	0.35, 1.04	0.34	0.17, 0.51
	BF_{10}	162.627	2.34e0-9						
TanningBeds-C-Incoherence	Student's t	1.75		539	0.08	0.25	-0.03, 0.53	0.15	-0.02, 0.32
	BF_{10}	0.427	1.10e0-6						
VideoGames-Incoherence	Student's t	9.2		542	< .001	1.54	1.21, 1.87	0.79	0.61, 0.97
	BF_{10}	5.67E+15	1.84E-23						
VideoGames-C-Incoherence	Student's t	3.2		540	0.001	0.51	0.20, 0.82	0.28	0.10, 0.45
	BF_{10}	13.5	3.02e0-8						
TrueOrFalse-Incoherence	Student's t	7.24		540	< .001	1.19	0.86, 1.51	0.62	0.45, 0.80
	BF_{10}	4.24e0+9	4.31E-17						
TrueOrFalse-C-Incoherence	Student's t	2.9		542	0.004	0.51	0.16, 0.85	0.25	0.08, 0.42
	BF_{10}	5.641	7.81e0-8						
Consensus-Incoherence	Student's t	6.9		542	< .001	1.25	0.90, 1.61	0.59	0.42, 0.77
	BF_{10}	4.79e0+8	4.41E-16						
Consensus-C-Incoherence	Student's t	5.52		540	< .001	0.95	0.61, 1.29	0.47	0.30, 0.65
	BF_{10}	186016.535	1.44E-12						
Predictions-Incoherence	Student's t	9.55		543	< .001	1.64	1.31, 1.98	0.82	0.64, 1.00
	BF_{10}	9.45E+16	1.00E-24						
Predictions-C-Incoherence	Student's t	1.01		539	0.313	0.15	-0.15, 0.45	0.09	-0.08, 0.26
	BF_{10}	0.157	3.15e0-6						
Temperatures-Incoherence	Student's t	10.6		543	< .001	1.81	1.48, 2.15	0.91	0.72, 1.09
	BF_{10}	6.29E+20	8.70E-29						
Temperatures-C-Incoherence	Student's t	3.86		539	< .001	0.60	0.29, 0.91	0.33	0.16, 0.50
	BF_{10}	125.02	2.87e0-9						
DataPrivacy-Incoherence	Student's t	10.56		541	< .001	1.90	1.54, 2.25	0.91	0.72, 1.09
	BF_{10}	4.11E+20	1.19E-28						
DataPrivacy-C-Incoherence	Student's t	1.37		541	0.172	0.23	-0.10, 0.57	0.12	-0.05, 0.29
	BF_{10}	0.237	2.12e0-6						
Beliefs-Incoherence	Student's t	6.58		541	< .001	1.07	0.75, 1.38	0.57	0.39, 0.74
	BF_{10}	6.98e0+7	3.19E-15						
Beliefs-C-Incoherence	Student's t	1.01		541	0.314	0.18	-0.17, 0.54	0.09	-0.08, 0.26
	BF_{10}	0.157	3.29e0-6						

[a] Levene's test is significant ($p < .05$), suggesting a violation of the assumption of equal variances

Table S8.

Study 2 (incoherence): confidence measure item-level results (independent samples and Bayesian *t*-tests). *Note:* Bayesian prior used is 0.707 (representing an 80% chance that the effect size is between -2 and 2). "-C-" denotes a manipulative post's matched neutral control.

Confidence		Statistic	±%	df	*p*	M_{diff}	95%CI	Cohen's *d*	95%CI
Manipulative-Confidence	Student's t	0.721		1084	0.471	0.05	-0.08, 0.18	0.04	-0.08, 0.16
	BF_{10}	0.0879	3.99E-04						
Neutral-Confidence	Student's t	-2.266		1082	0.024	-0.15	-0.27, -0.02	-0.14	-0.26, -0.02
	BF_{10}	0.8515	3.88E-05						
Discernment-Confidence	Student's t	3.9		1082	< .001	0.19	0.10, 0.29	0.24	0.12, 0.36
	BF_{10}	117.5321	2.57E-07						
StockMarket-Confidence	Student's t	0.249		543	0.804	0.03	-0.22, 0.28	0.02	-0.15, 0.19
	BF_{10}	0.0984	5.40E-06						
StockMarket-C-Confidence	Student's t	-1.05		539	0.294	-0.13	-0.38, 0.12	-0.09	-0.26, 0.08
	BF_{10}	0.164	2.88E-06						
Morality-Confidence	Student's t	1.972		543	0.049	0.24	0.00, 0.48	0.17	0.00, 0.34
	BF_{10}	0.6305	7.86E-07						
Morality-C-Confidence	Student's t	-0.423		539	0.672	-0.05	-0.28, 0.18	-0.04	-0.20, 0.13
	BF_{10}	0.1044	4.87E-06						
TanningBeds-Confidence	Student's t	-2.287		543	0.023	-0.29	-0.54, -0.04	-0.20	-0.36, -0.03
	BF_{10}	1.2079	3.91E-07						
TanningBeds-C-Confidence	Student's t	-2.201		539	0.028	-0.29	-0.54, -0.03	-0.19	-0.36, -0.02
	BF_{10}	1.0039	4.48E-07						
VideoGames-Confidence	Student's t	-0.312		542	0.755	-0.04	-0.28, 0.20	-0.03	-0.19, 0.14
	BF_{10}	0.1001	5.34E-06						
VideoGames-C-Confidence	Student's t	-1.643		540	0.101	-0.21	-0.47, 0.04	-0.14	-0.31, 0.03
	BF_{10}	0.355	1.37E-06						
TrueOrFalse-Confidence	Student's t	3.9		540	< .001	0.45	0.22, 0.68	0.34	0.16, 0.51
	BF_{10}	144.3469	2.54E-09						
TrueOrFalse-C-Confidence	Student's t	-2.977		542	0.003	-0.37	-0.61, -0.13	-0.26	-0.42, -0.09
	BF_{10}	6.9478	6.28E-08						
Consensus-Confidence	Student's t	-1.989		542	0.047	-0.25	-0.50, 0.00	-0.17	-0.34, 0.00
	BF_{10}	0.6523	7.43E-07						
Consensus-C-Confidence	Student's t	-0.243		540	0.808	-0.03	-0.29, 0.23	-0.02	-0.19, 0.15
	BF_{10}	0.0985	5.18E-06						
Predictions-Confidence	Student's t	0.94		543	0.347	0.12	-0.13, 0.37	0.08	-0.09, 0.25
	BF_{10}	0.1466	3.63E-06						
Predictions-C-Confidence	Student's t	-0.719		539	0.472	-0.09	-0.33, 0.15	-0.06	-0.23, 0.11
	BF_{10}	0.1231	4.07E-06						
Temperatures-Confidence	Student's t	0.736		543	0.462	0.09	-0.15, 0.34	0.06	-0.11, 0.23
	BF_{10}	0.1241	4.33E-06						
Temperatures-C-Confidence	Student's t	-0.44		539	0.66	-0.05	-0.30, 0.19	-0.04	-0.21, 0.13
	BF_{10}	0.1052	4.79E-06						
DataPrivacy-Confidence	Student's t	1.713		541	0.087	0.23	-0.03, 0.48	0.15	-0.02, 0.32
	BF_{10}	0.3976	1.24E-06						
DataPrivacy-C-Confidence	Student's t	-3.074		541	0.002	-0.38	-0.63, -0.14	-0.26	-0.43, -0.09
	BF_{10}	9.2291	4.59E-08						
Beliefs-Confidence	Student's t	2.036		541	0.042	0.24	0.01, 0.48	0.17	0.01, 0.34
	BF_{10}	0.7143	6.68E-07						
Beliefs-C-Confidence	Student's t	-1.145		541	0.253	-0.15	-0.40, 0.11	-0.10	-0.27, 0.07
	BF_{10}	0.1808	2.82E-06						

[a] Levene's test is significant ($p < .05$), suggesting a violation of the assumption of equal variances

Table S9.

Study 2 (incoherence): trustworthiness measure item-level results (independent samples and Bayesian *t*-tests). *Note:* Bayesian prior used is 0.707 (representing an 80% chance that the effect size is between -2 and 2). "-C-" denotes a manipulative post's matched neutral control.

Trustworthiness		Statistic	±%	df	*p*	M_{diff}	95%CI	Cohen's *d*	95%CI
Manipulative-Trustworthiness	Student's t	-4.724		1084	< .001	-0.33	-0.47, -0.19	-0.29	-0.41, -0.17
	BF_{10}	3712.974	7.83e0-9						
Neutral-Trustworthiness	Student's t	-1.499		1082	0.134	-0.10	-0.22, 0.03	-0.09	-0.21, 0.03
	BF_{10}	0.206	1.65e0-4						
Discernment-Trustworthiness	Student's t	3.11		1082	0.002	0.24	0.09, 0.38	0.19	0.07, 0.31
	BF_{10}	7.876	4.03e0-6						
StockMarket-Trustworthiness	Student's t	-3.54		543	< .001	-0.49	-0.77, -0.22	-0.30	-0.47, -0.13
	BF_{10}	40.336	9.92e0-9						
StockMarket-C-Trustworthiness	Student's t	-2.834		539	0.005	-0.38	-0.65, -0.12	-0.24	-0.41, -0.07
	BF_{10}	4.673	8.54e0-8						
Morality-Trustworthiness	Student's t	-4.793		543	< .001	-0.70	-0.99, -0.41	-0.41	-0.58, -0.24
	BF_{10}	5661.492	5.95E-11						
Morality-C-Trustworthiness	Student's t	-3.799		539	< .001	-0.55	-0.83, -0.26	-0.33	-0.50, -0.16
	BF_{10}	99.881	3.67e0-9						
TanningBeds-Trustworthiness	Student's t	1.089		543	0.277	0.15	-0.12, 0.43	0.09	-0.08, 0.26
	BF_{10}	0.17	3.06e0-6						
TanningBeds-C-Trustworthiness	Student's t	-0.744		539	0.457	-0.10	-0.36, 0.16	-0.06	-0.23, 0.10
	BF_{10}	0.125	3.99e0-6						
VideoGames-Trustworthiness	Student's t	-3.128		542	0.002	-0.43	-0.70, -0.16	-0.27	-0.44, -0.10
	BF_{10}	10.827	3.95e0-8						
VideoGames-C-Trustworthiness	Student's t	1.425		540	0.155	0.20	-0.08, 0.49	0.12	-0.05, 0.29
	BF_{10}	0.257	1.92e0-6						
TrueOrFalse-Trustworthiness	Student's t	-1.237		540	0.217	-0.18	-0.47, 0.11	-0.11	-0.27, 0.06
	BF_{10}	0.201	2.48e0-6						
TrueOrFalse-C-Trustworthiness	Student's t	-1.433		542	0.152	-0.21	-0.51, 0.08	-0.12	-0.29, 0.05
	BF_{10}	0.259	1.97e0-6						
Consensus-Trustworthiness	Student's t	0.519		542	0.604	0.08	-0.22, 0.38	0.04	-0.12, 0.21
	BF_{10}	0.109	4.87e0-6						
Consensus-C-Trustworthiness	Student's t	-3.714		540	< .001	-0.56	-0.86, -0.26	-0.32	-0.49, -0.15
	BF_{10}	73.605	5.04e0-9						
Predictions-Trustworthiness	Student's t	-2.39		543	0.017	-0.32	-0.58, -0.06	-0.20	-0.37, -0.04
	BF_{10}	1.522	3.13e0-7						
Predictions-C-Trustworthiness	Student's t	0.805		539	0.421	0.11	-0.15, 0.37	0.07	-0.10, 0.24
	BF_{10}	0.131	3.80e0-6						
Temperatures-Trustworthiness	Student's t	-0.585		543	0.559	-0.08	-0.33, 0.18	-0.05	-0.22, 0.12
	BF_{10}	0.113	4.79e0-6						
Temperatures-C-Trustworthiness	Student's t	-0.325		539	0.745	-0.05	-0.34, 0.24	-0.03	-0.20, 0.14
	BF_{10}	0.101	5.01e0-6						
DataPrivacy-Trustworthiness	Student's t	-7.856		541	< .001	-1.21	-1.51, -0.91	-0.67	-0.85, -0.50
	BF_{10}	2.64E+11	6.03E-19						
DataPrivacy-C-Trustworthiness	Student's t	-0.426		541	0.67	-0.06	-0.35, 0.22	-0.04	-0.20, 0.13
	BF_{10}	0.104	5.03e0-6						
Beliefs-Trustworthiness	Student's t	-2.093		541	0.037	-0.27	-0.52, -0.02	-0.18	-0.35, -0.01
	BF_{10}	0.801	5.92e0-7						
Beliefs-C-Trustworthiness	Student's t	2.935		541	0.003	0.41	0.14, 0.69	0.25	0.08, 0.42
	BF_{10}	6.168	6.99e0-8						

[a] Levene's test is significant ($p < .05$), suggesting a violation of the assumption of equal variances

Table S10.

Study 2 (incoherence): sharing intentions measure item-level results (independent samples and Bayesian *t*-tests). *Note:* Bayesian prior used is 0.707 (representing an 80% chance that the effect size is between -2 and 2). "-C-" denotes a manipulative post's matched neutral control.

**Sharing**		**Statistic**	**±%**	**df**	***p***	**M_{diff}**	**95%CI**	**Cohen's *d***	**95%CI**
Manipulative-Sharing	Student's t	-1.1953		1084	0.232	-0.09	-0.23, 0.06	-0.07	-0.19, 0.05
	BF_{10}	0.1375	2.53E-04						
Neutral-Sharing	Student's t	0.0464		1082	0.963	0.00	-0.16, 0.17	0.00	-0.12, 0.12
	BF_{10}	0.0681	5.08E-04						
Discernment-Sharing	Student's t	1.6036		1082	0.109	0.10	-0.02, 0.22	0.10	-0.02, 0.22
	BF_{10}	0.2417	1.40E-04						
StockMarket-Sharing	Student's t	0.7448		543	0.457	0.10	-0.17, 0.38	0.06	-0.10, 0.23
	BF_{10}	0.1251	4.19E-06						
StockMarket-C-Sharing	Student's t	-1.4887		539	0.137	-0.20	-0.47, 0.06	-0.13	-0.30, 0.04
	BF_{10}	0.2817	1.63E-06						
Morality-Sharing	Student's t	-1.7183		543	0.086	-0.25	-0.53, 0.04	-0.15	-0.32, 0.02
	BF_{10}	0.4004	1.27E-06						
Morality-C-Sharing	Student's t	-2.1472		539	0.032	-0.32	-0.62, -0.03	-0.18	-0.35, -0.02
	BF_{10}	0.8966	5.08E-07						
TanningBeds-Sharing	Student's t	0.2995		543	0.765	0.04	-0.22, 0.31	0.03	-0.14, 0.19
	BF_{10}	0.0997	5.36E-06						
TanningBeds-C-Sharing	Student's t	0.6516		539	0.515	0.11	-0.22, 0.44	0.06	-0.11, 0.22
	BF_{10}	0.1177	4.26E-06						
VideoGames-Sharing	Student's t	-1.3248		542	0.186	-0.17	-0.43, 0.08	-0.11	-0.28, 0.05
	BF_{10}	0.2241	2.29E-06						
VideoGames-C-Sharing	Student's t	0.9795		540	0.328	0.14	-0.14, 0.42	0.08	-0.08, 0.25
	BF_{10}	0.1525	3.32E-06						
TrueOrFalse-Sharing	Student's t	-1.3688		540	0.172	-0.19	-0.46, 0.08	-0.12	-0.29, 0.05
	BF_{10}	0.2378	2.08E-06						
TrueOrFalse-C-Sharing	Student's t	-0.0649		542	0.948	-0.01	-0.32, 0.30	-0.01	-0.17, 0.16
	BF_{10}	0.0956	5.61E-06						
Consensus-Sharing	Student's t	-0.6266		542	0.531	-0.09	-0.35, 0.18	-0.05	-0.22, 0.11
	BF_{10}	0.1155	4.57E-06						
Consensus-C-Sharing	Student's t	-1.4069		540	0.16	-0.24	-0.57, 0.09	-0.12	-0.29, 0.05
	BF_{10}	0.2505	1.94E-06						
Predictions-Sharing	Student's t	-0.6986		543	0.485	-0.09	-0.34, 0.16	-0.06	-0.23, 0.11
	BF_{10}	0.1209	4.45E-06						
Predictions-C-Sharing	Student's t	2.2643		539	0.024	0.38	0.05, 0.71	0.19	0.03, 0.36
	BF_{10}	1.1512	3.88E-07						
Temperatures-Sharing	Student's t	-1.6652		543	0.096	-0.19	-0.42, 0.03	-0.14	-0.31, 0.03
	BF_{10}	0.367	1.39E-06						
Temperatures-C-Sharing	Student's t	-0.2643		539	0.792	-0.04	-0.38, 0.29	-0.02	-0.19, 0.15
	BF_{10}	0.099	5.11E-06						
DataPrivacy-Sharing	Student's t	-2.2909		541	0.022	-0.33	-0.62, -0.05	-0.20	-0.37, -0.03
	BF_{10}	1.2186	3.82E-07						
DataPrivacy-C-Sharing	Student's t	1.0127		541	0.312	0.16	-0.15, 0.46	0.09	-0.08, 0.26
	BF_{10}	0.1573	3.27E-06						
Beliefs-Sharing	Student's t	-1.1102		541	0.267	-0.12	-0.33, 0.09	-0.10	-0.26, 0.07
	BF_{10}	0.174	2.94E-06						
Beliefs-C-Sharing	Student's t	1.7352		541	0.083	0.22	-0.03, 0.46	0.15	-0.02, 0.32
	BF_{10}	0.4126	1.19E-06						

[a] Levene's test is significant ($p < .05$), suggesting a violation of the assumption of equal variances

Table S11.

Study 3 (false dichotomies): technique recognition measure item-level results (independent samples and Bayesian *t*-tests). *Note:* Bayesian prior used is 0.707 (representing an 80% chance that the effect size is between -2 and 2). "-C-" denotes a manipulative post's matched neutral control.

**False dichotomy**		**Statistic**	**±%**	**df**	***p***	M_{diff}	**95%CI**	**Cohen's *d***	**95%CI**
Manipulative-Dichotomy	Student's t	13.369		1092	< .001	1.01	0.86, 1.16	0.81	0.68, 0.94
	BF_{10}	2.81E+34	3.95E-40						
Neutral-Dichotomy	Student's t	-1.502		1091	0.133	-0.10	-0.24, 0.03	-0.09	-0.21, 0.03
	BF_{10}	0.2061	1.78e0-4						
Discernment-Dichotomy	Student's t	11.287		1091	< .001	1.10	0.91, 1.29	0.68	0.56, 0.81
	BF_{10}	5.46E+24	2.81E-30						
Business-Dichotomy	Student's t	6.592		546	< .001	1.00	0.70, 1.30	0.56	0.39, 0.74
	BF_{10}	7.41e0+7	3.38E-15						
Business-C-Dichotomy	Student's t	-0.859		545	0.391	-0.13	-0.42, 0.17	-0.07	-0.24, 0.09
	BF_{10}	0.1363	4.05e0-6						
Coffee-Dichotomy	Student's t	6.988		544	< .001	1.06	0.76, 1.36	0.60	0.42, 0.77
	BF_{10}	8.46e0+8	2.46E-16						
Coffee-C-Dichotomy	Student's t	-1.503		547	0.134	-0.21	-0.48, 0.06	-0.13	-0.30, 0.04
	BF_{10}	0.285	1.91e0-6						
Combat-Dichotomy	Student's t	6.683		545	< .001	1.04	0.73, 1.34	0.57	0.40, 0.75
	BF_{10}	1.28e0+8	1.90E-15						
Combat-C-Dichotomy	Student's t	1.439		546	0.151	0.22	-0.08, 0.53	0.12	-0.05, 0.29
	BF_{10}	0.2603	2.09e0-6						
Education-Dichotomy	Student's t	5.087		540	< .001	0.84	0.51, 1.16	0.44	0.26, 0.61
	BF_{10}	22133.0957	1.31E-11						
Education-C-Dichotomy	Student's t	-1.948		551	0.052	-0.29	-0.59, 0.00	-0.17	-0.33, 0.00
	BF_{10}	0.5989	9.42e0-7						
JohnLewis-Dichotomy	Student's t	4.516		544	< .001	0.72	0.41, 1.03	0.39	0.22, 0.56
	BF_{10}	1686.2847	2.10E-10						
JohnLewis-C-Dichotomy	Student's t	-3.341		547	< .001	-0.48	-0.77, -0.20	-0.29	-0.45, -0.12
	BF_{10}	20.8557	2.18e0-8						
Party-Dichotomy	Student's t	6.945		545	< .001	1.13	0.81, 1.45	0.59	0.42, 0.77
	BF_{10}	6.43e0+8	3.57E-16						
Party-C-Dichotomy	Student's t	0.598		546	0.55	0.09	-0.21, 0.39	0.05	-0.12, 0.22
	BF_{10}	0.1132	4.93e0-6						
Potatoes-Dichotomy	Student's t	6.654		546	< .001	1.03	0.73, 1.33	0.57	0.39, 0.74
	BF_{10}	1.07e0+8	2.25E-15						
Potatoes-C-Dichotomy	Student's t	-0.186		545	0.852	-0.03	-0.34, 0.28	-0.02	-0.18, 0.15
	BF_{10}	0.0971	5.43e0-6						
Problem-Dichotomy	Student's t	10.273		545	< .001	1.62	1.31, 1.93	0.88	0.69, 1.06
	BF_{10}	3.72E+19	1.77E-24						
Problem-C-Dichotomy	Student's t	3.528		546	< .001	0.55	0.24, 0.86	0.30	0.13, 0.47
	BF_{10}	38.614	1.13e0-8						
SocServ-Dichotomy	Student's t	3.625		540	< .001	0.62	0.28, 0.96	0.31	0.14, 0.48
	BF_{10}	53.9144	6.99e0-9						
SocServ-C-Dichotomy	Student's t	-1.457		551	0.146	-0.23	-0.55, 0.08	-0.12	-0.29, 0.04
	BF_{10}	0.266	2.20e0-6						
Surveillance-Dichotomy	Student's t	6.123		545	< .001	0.97	0.66, 1.28	0.52	0.35, 0.70
	BF_{10}	4.84e0+6	5.68E-14						
Surveillance-C-Dichotomy	Student's t	1.652		546	0.099	0.26	-0.05, 0.57	0.14	-0.03, 0.31
	BF_{10}	0.3583	1.48e0-6						

[a] Levene's test is significant ($p < .05$), suggesting a violation of the assumption of equal variances

Table S12.

Study 3 (false dichotomies): confidence measure item-level results (independent samples and Bayesian *t*-tests). *Note:* Bayesian prior used is 0.707 (representing an 80% chance that the effect size is between -2 and 2). “-C-“ denotes a manipulative post’s matched neutral control.

Confidence		Statistic	±%	df	*p*	M_{diff}	95%CI	Cohen's *d*	95%CI
Manipulative-Confidence	Student's t	7.919		1092	< .001	0.52	0.39, 0.65	0.48	0.36, 0.60
	BF_{10}	8.41E+11	2.80E-17						
Neutral-Confidence	Student's t	4.024		1091	< .001	0.28	0.15, 0.42	0.24	0.12, 0.36
	BF_{10}	189.802	1.72e0-7						
Discernment-Confidence	Student's t	4.059		1091	< .001	0.23	0.12, 0.34	0.25	0.13, 0.37
	BF_{10}	217.04	1.50e0-7						
Business-Confidence	Student's t	5.438		546	< .001	0.67	0.43, 0.92	0.46	0.29, 0.64
	BF_{10}	124228.696	2.54E-12						
Business-C-Confidence	Student's t	0.779		545	0.436	0.10	-0.15, 0.35	0.07	-0.10, 0.23
	BF_{10}	0.128	4.33e0-6						
Coffee-Confidence	Student's t	4.754		544	< .001	0.62	0.36, 0.87	0.41	0.24, 0.58
	BF_{10}	4768.04	6.96E-11						
Coffee-C-Confidence	Student's t	2.131		547	0.034	0.28	0.02, 0.53	0.18	0.01, 0.35
	BF_{10}	0.861	6.02e0-7						
Combat-Confidence	Student's t	5.128		545	< .001	0.68	0.42, 0.94	0.44	0.27, 0.61
	BF_{10}	26937.439	1.23E-11						
Combat-C-Confidence	Student's t	1.719		546	0.086	0.22	-0.03, 0.48	0.15	-0.02, 0.32
	BF_{10}	0.4	1.33e0-6						
Education-Confidence	Student's t	2.333		540	0.02	0.30	0.05, 0.55	0.20	0.03, 0.37
	BF_{10}	1.339	3.32e0-7						
Education-C-Confidence	Student's t	2.406		551	0.016	0.31	0.06, 0.56	0.20	0.04, 0.37
	BF_{10}	1.571	3.45e0-7						
JohnLewis-Confidence	Student's t	3.008		544	0.003	0.38	0.13, 0.63	0.26	0.09, 0.43
	BF_{10}	7.588	5.85e0-8						
JohnLewis-C-Confidence	Student's t	4.22		547	< .001	0.57	0.30, 0.83	0.36	0.19, 0.53
	BF_{10}	494.601	8.13E-10						
Party-Confidence	Student's t	3.313		545	< .001	0.43	0.17, 0.68	0.28	0.11, 0.45
	BF_{10}	19.125	2.31e0-8						
Party-C-Confidence	Student's t	2.433		546	0.015	0.32	0.06, 0.58	0.21	0.04, 0.38
	BF_{10}	1.681	2.92e0-7						
Potatoes-Confidence	Student's t	4.028		546	< .001	0.48	0.25, 0.71	0.34	0.17, 0.51
	BF_{10}	234.221	1.68e0-9						
Potatoes-C-Confidence	Student's t	2.652		545	0.008	0.35	0.09, 0.60	0.23	0.06, 0.40
	BF_{10}	2.882	1.55e0-7						
Problem-Confidence	Student's t	5.919		545	< .001	0.70	0.47, 0.94	0.51	0.33, 0.68
	BF_{10}	1.56e0+6	1.83E-13						
Problem-C-Confidence	Student's t	2.456		546	0.014	0.32	0.06, 0.58	0.21	0.04, 0.38
	BF_{10}	1.774	2.80e0-7						
SocServ-Confidence	Student's t	3.039		540	0.002	0.38	0.13, 0.63	0.26	0.09, 0.43
	BF_{10}	8.334	4.92e0-8						
SocServ-C-Confidence	Student's t	2.82		551	0.005	0.38	0.11, 0.64	0.24	0.07, 0.41
	BF_{10}	4.452	1.17e0-7						
Surveillance-Confidence	Student's t	3.088		545	0.002	0.40	0.14, 0.65	0.26	0.09, 0.43
	BF_{10}	9.57	4.75e0-8						
Surveillance-C-Confidence	Student's t	2.967		546	0.003	0.38	0.13, 0.63	0.25	0.08, 0.42
	BF_{10}	6.73	6.91e0-8						

[a] Levene's test is significant (p < .05), suggesting a violation of the assumption of equal variances

Table S13.

Study 3 (false dichotomies): trustworthiness measure item-level results (independent samples and Bayesian *t*-tests). *Note:* Bayesian prior used is 0.707 (representing an 80% chance that the effect size is between -2 and 2). "-C-" denotes a manipulative post's matched neutral control.

Trustworthiness		Statistic	±%	df	*p*	M_{diff}	95%CI	Cohen's *d*	95%CI
Manipulative-Trustworthiness	Student's t	-3.931		1092	< .001	-0.29	-0.44, -0.15	-0.24	-0.36, -0.12
	BF_{10}	131.965	2.50e0-7						
Neutral-Trustworthiness	Student's t	1.192		1091	0.233	0.08	-0.05, 0.20	0.07	-0.05, 0.19
	BF_{10}	0.137	2.70e0-4						
Discernment-Trustworthiness	Student's t	5.293		1091	< .001	0.37	0.24, 0.51	0.32	0.20, 0.44
	BF_{10}	58111.555	5.11E-10						
Business-Trustworthiness	Student's t	-1.98		546	0.048	-0.28	-0.56, 0.00	-0.17	-0.34, -0.0009
	BF_{10}	0.638	8.07e0-7						
Business-C-Trustworthiness	Student's t	0.608		545	0.543	0.08	-0.17, 0.32	0.05	-0.12, 0.22
	BF_{10}	0.114	4.89e0-6						
Coffee-Trustworthiness	Student's t	-1.227		544	0.22	-0.17	-0.44, 0.10	-0.11	-0.27, 0.06
	BF_{10}	0.199	2.60e0-6						
Coffee-C-Trustworthiness	Student's t	-1.377		547	0.169	-0.16	-0.39, 0.07	-0.12	-0.29, 0.05
	BF_{10}	0.239	2.30e0-6						
Combat-Trustworthiness	Student's t	-2.927		545	0.004	-0.42	-0.70, -0.14	-0.25	-0.42, -0.08
	BF_{10}	6.014	7.69e0-8						
Combat-C-Trustworthiness	Student's t	-1.889		546	0.059	-0.24	-0.50, 0.01	-0.16	-0.33, 0.01
	BF_{10}	0.538	9.75e0-7						
Education-Trustworthiness	Student's t	-2.11		540	0.035	-0.31	-0.59, -0.02	-0.18	-0.35, -0.01
	BF_{10}	0.831	5.48e0-7						
Education-C-Trustworthiness	Student's t	0.986		551	0.325	0.12	-0.12, 0.36	0.08	-0.08, 0.25
	BF_{10}	0.152	3.95e0-6						
JohnLewis-Trustworthiness	Student's t	-1.947		544	0.052	-0.26	-0.53, 0.00	-0.17	-0.34, 0.00
	BF_{10}	0.601	8.30e0-7						
JohnLewis-C-Trustworthiness	Student's t	1.357		547	0.175	0.18	-0.08, 0.44	0.12	-0.05, 0.28
	BF_{10}	0.233	2.38e0-6						
Party-Trustworthiness	Student's t	-0.906		545	0.365	-0.13	-0.41, 0.15	-0.08	-0.25, 0.09
	BF_{10}	0.142	3.88e0-6						
Party-C-Trustworthiness	Student's t	1.121		546	0.263	0.15	-0.12, 0.42	0.10	-0.07, 0.26
	BF_{10}	0.175	3.12e0-6						
Potatoes-Trustworthiness	Student's t	-2.359		546	0.019	-0.33	-0.60, -0.06	-0.20	-0.37, -0.03
	BF_{10}	1.418	3.45e0-7						
Potatoes-C-Trustworthiness	Student's t	3.113		545	0.002	0.41	0.15, 0.66	0.27	0.10, 0.44
	BF_{10}	10.345	4.07e0-8						
Problem-Trustworthiness	Student's t	-5.175		545	< .001	-0.79	-1.09, -0.49	-0.44	-0.61, -0.27
	BF_{10}	33816.269	9.72E-12						
Problem-C-Trustworthiness	Student's t	-0.578		546	0.563	-0.08	-0.34, 0.19	-0.05	-0.22, 0.12
	BF_{10}	0.112	5.05e0-6						
SocServ-Trustworthiness	Student's t	-1.492		540	0.136	-0.24	-0.55, 0.07	-0.13	-0.30, 0.04
	BF_{10}	0.282	1.71e0-6						
SocServ-C-Trustworthiness	Student's t	3.25		551	0.001	0.41	0.16, 0.65	0.28	0.11, 0.44
	BF_{10}	15.613	3.16e0-8						
Surveillance-Trustworthiness	Student's t	-0.777		545	0.438	-0.11	-0.39, 0.17	-0.07	-0.23, 0.10
	BF_{10}	0.128	4.34e0-6						
Surveillance-C-Trustworthiness	Student's t	-0.364		546	0.716	-0.04	-0.28, 0.19	-0.03	-0.20, 0.14
	BF_{10}	0.101	5.56e0-6						

[a] Levene's test is significant (p < .05), suggesting a violation of the assumption of equal variances

Table S14.

Study 3 (false dichotomies): sharing intentions measure item-level results (independent samples and Bayesian *t*-tests). *Note:* Bayesian prior used is 0.707 (representing an 80% chance that the effect size is between -2 and 2). “-C-“ denotes a manipulative post’s matched neutral control.

Sharing		Statistic	±%	df	*p*	M_{diff}	95%CI	Cohen's *d*	95%CI
Manipulative-Sharing	Student's t	-2.178		1092	0.03	-0.19	-0.35, -0.02	-0.13	-0.25, -0.01
	BF_{10}	0.7001	5.16E-05						
Neutral-Sharing	Student's t	0.444		1091	0.657	0.04	-0.14, 0.22	0.03	-0.09, 0.15
	BF_{10}	0.0747	4.99E-04						
Discernment-Sharing	Student's t	3.602		1091	< .001	0.22	0.10, 0.35	0.22	0.10, 0.34
	BF_{10}	39.4482	8.47E-07						
Business-Sharing	Student's t	-1.817		546	0.07	-0.26	-0.53, 0.02	-0.16	-0.32, 0.01
	BF_{10}	0.4732	1.11E-06						
Business-C-Sharing	Student's t	0.225		545	0.822	0.04	-0.27, 0.34	0.02	-0.15, 0.19
	BF_{10}	0.0975	5.75E-06						
Coffee-Sharing	Student's t	-0.741		544	0.459	-0.11	-0.39, 0.18	-0.06	-0.23, 0.10
	BF_{10}	0.1246	4.24E-06						
Coffee-C-Sharing	Student's t	-0.823		547	0.411	-0.14	-0.49, 0.20	-0.07	-0.24, 0.10
	BF_{10}	0.1322	4.28E-06						
Combat-Sharing	Student's t	-0.81		545	0.418	-0.11	-0.38, 0.16	-0.07	-0.24, 0.10
	BF_{10}	0.131	4.21E-06						
Combat-C-Sharing	Student's t	-1.608		546	0.108	-0.22	-0.49, 0.05	-0.14	-0.31, 0.03
	BF_{10}	0.3344	1.60E-06						
Education-Sharing	Student's t	-0.173		540	0.863	-0.03	-0.35, 0.29	-0.01	-0.18, 0.15
	BF_{10}	0.0971	5.24E-06						
Education-C-Sharing	Student's t	-0.624		551	0.533	-0.11	-0.46, 0.24	-0.05	-0.22, 0.11
	BF_{10}	0.1145	5.31E-06						
JohnLewis-Sharing	Student's t	-0.789		544	0.43	-0.11	-0.40, 0.17	-0.07	-0.24, 0.10
	BF_{10}	0.1291	4.16E-06						
JohnLewis-C-Sharing	Student's t	0.477		547	0.633	0.06	-0.20, 0.33	0.04	-0.13, 0.21
	BF_{10}	0.1061	5.42E-06						
Party-Sharing	Student's t	-0.797		545	0.426	-0.11	-0.39, 0.16	-0.07	-0.24, 0.10
	BF_{10}	0.1297	4.27E-06						
Party-C-Sharing	Student's t	1.005		546	0.315	0.16	-0.15, 0.46	0.09	-0.08, 0.25
	BF_{10}	0.1556	3.53E-06						
Potatoes-Sharing	Student's t	-2.576		546	0.01	-0.38	-0.67, -0.09	-0.22	-0.39, -0.05
	BF_{10}	2.3774	2.01E-07						
Potatoes-C-Sharing	Student's t	0.695		545	0.487	0.11	-0.20, 0.41	0.06	-0.11, 0.23
	BF_{10}	0.1208	4.31E-06						
Problem-Sharing	Student's t	-2.643		545	0.008	-0.44	-0.77, -0.11	-0.23	-0.39, -0.06
	BF_{10}	2.8099	1.70E-07						
Problem-C-Sharing	Student's t	-0.963		546	0.336	-0.15	-0.45, 0.15	-0.08	-0.25, 0.09
	BF_{10}	0.1494	3.73E-06						
SocServ-Sharing	Student's t	-1.262		540	0.207	-0.20	-0.51, 0.11	-0.11	-0.28, 0.06
	BF_{10}	0.2077	2.37E-06						
SocServ-C-Sharing	Student's t	1.953		551	0.051	0.34	0.00, 0.68	0.17	0.00, 0.33
	BF_{10}	0.6042	9.35E-07						
Surveillance-Sharing	Student's t	-0.697		545	0.486	-0.11	-0.41, 0.20	-0.06	-0.23, 0.11
	BF_{10}	0.1206	4.61E-06						
Surveillance-C-Sharing	Student's t	0.505		546	0.614	0.08	-0.24, 0.40	0.04	-0.12, 0.21
	BF_{10}	0.1077	5.22E-06						

[a] Levene's test is significant ($p < .05$), suggesting a violation of the assumption of equal variances

Table S15.

Study 4 (scapegoating): technique recognition measure item-level results (independent samples and Bayesian *t*-tests). *Note:* Bayesian prior used is 0.707 (representing an 80% chance that the effect size is between -2 and 2). "-C-" denotes a manipulative post's matched neutral control.

**Scapegoating**		**Statistic**	**±%**	**df**	***p***	**M_{diff}**	**95%CI**	**Cohen's *d***	**95%CI**
Manipulative-Scapegoating	Student's t	6.634		1076	< .001	0.46	0.33, 0.60	0.40	0.28, 0.53
	BF_{10}	1.23E+08	1.84E-13						
Neutral-Scapegoating	Student's t	-0.442		1077	0.658	-0.03	-0.17, 0.11	-0.03	-0.15, 0.09
	BF_{10}	0.0751	4.40e0-4						
Discernment-Scapegoating	Student's t	4.525		1077	< .001	0.47	0.27, 0.68	0.28	0.15, 0.40
	BF_{10}	1521.5281	1.81e0-8						
Fiscal-Scapegoating	Student's t	2.874		537	0.004	0.35	0.11, 0.59	0.25	0.08, 0.42
	BF_{10}	5.2325	7.24e0-8						
Fiscal-C-Scapegoating	Student's t	0.515		539	0.607	0.08	-0.23, 0.39	0.04	-0.12, 0.21
	BF_{10}	0.1091	4.48e0-6						
Freewill-Scapegoating	Student's t	4.612		531	< .001	0.62	0.36, 0.89	0.40	0.23, 0.57
	BF_{10}	2554.7549	1.02E-10						
Freewill-C-Scapegoating	Student's t	-1.541		545	0.124	-0.20	-0.45, 0.05	-0.13	-0.30, 0.04
	BF_{10}	0.3021	1.74e0-6						
Gamers-Scapegoating	Student's t	4.008		535	< .001	0.52	0.27, 0.78	0.35	0.17, 0.52
	BF_{10}	217.4438	1.49e0-9						
Gamers-C-Scapegoating	Student's t	-0.647		541	0.518	-0.09	-0.38, 0.19	-0.06	-0.22, 0.11
	BF_{10}	0.1171	4.46e0-6						
Morality-Scapegoating	Student's t	6.679		538	< .001	0.83	0.59, 1.07	0.58	0.40, 0.75
	BF_{10}	1.24E+08	1.60E-15						
Morality-C-Scapegoating	Student's t	2.461		538	0.014	0.42	0.08, 0.75	0.21	0.04, 0.38
	BF_{10}	1.8053	2.39e0-7						
Nuclear-Scapegoating	Student's t	3.416		543	< .001	0.50	0.21, 0.79	0.29	0.12, 0.46
	BF_{10}	26.6768	1.57e0-8						
Nuclear-C-Scapegoating	Student's t	-0.332		533	0.74	-0.05	-0.32, 0.22	-0.03	-0.20, 0.14
	BF_{10}	0.1015	4.52e0-6						
Politicians-Scapegoating	Student's t	4.948		535	< .001	0.65	0.39, 0.90	0.43	0.25, 0.60
	BF_{10}	11537.1816	2.32E-11						
Politicians-C-Scapegoating	Student's t	0.3		541	0.764	0.04	-0.23, 0.32	0.03	-0.14, 0.19
	BF_{10}	0.0999	5.19e0-6						
Professors-Scapegoating	Student's t	5.187		540	< .001	0.71	0.44, 0.98	0.45	0.27, 0.62
	BF_{10}	35761.0014	7.69E-12						
Professors-C-Scapegoating	Student's t	-1.043		536	0.297	-0.15	-0.44, 0.13	-0.09	-0.26, 0.08
	BF_{10}	0.1634	2.71e0-6						
Referees-Scapegoating	Student's t	4.129		542	< .001	0.56	0.30, 0.83	0.35	0.18, 0.52
	BF_{10}	346.5554	1.06e0-9						
Referees-C-Scapegoating	Student's t	-1.635		534	0.103	-0.24	-0.53, 0.05	-0.14	-0.31, 0.03
	BF_{10}	0.3526	1.23e0-6						
Struggle-Scapegoating	Student's t	-0.375		541	0.708	-0.06	-0.38, 0.26	-0.03	-0.20, 0.14
	BF_{10}	0.1023	5.13e0-6						
Struggle-C-Scapegoating	Student's t	-1.621		535	0.106	-0.24	-0.53, 0.05	-0.14	-0.31, 0.03
	BF_{10}	0.3447	1.28e0-6						
Turnout-Scapegoating	Student's t	2.293		539	0.022	0.31	0.04, 0.57	0.20	0.03, 0.37
	BF_{10}	1.2246	3.66e0-7						
Turnout-C-Scapegoating	Student's t	-1.394		537	0.164	-0.20	-0.47, 0.08	-0.12	-0.29, 0.05
	BF_{10}	0.2465	1.91e0-6						

[a] Levene's test is significant ($p < .05$), suggesting a violation of the assumption of equal variances

Table S16.

Study 4 (scapegoating): confidence measure item-level results (independent samples and Bayesian *t*-tests). *Note:* Bayesian prior used is 0.707 (representing an 80% chance that the effect size is between -2 and 2). "-C-" denotes a manipulative post's matched neutral control.

Confidence		Statistic	±%	df	*p*	M_{diff}	95%CI	Cohen's *d*	95%CI
Manipulative-Confidence	Student's t	5.801		1076	< .001	0.39	0.26, 0.52	0.35	0.23, 0.47
	BF_{10}	874068.0666	2.80E-11						
Neutral-Confidence	Student's t	1.891		1077	0.059	0.13	-0.01, 0.27	0.12	0.00, 0.24
	BF_{10}	0.3968	8.07e0-5						
Discernment-Confidence	Student's t	3.504		1077	< .001	0.23	0.10, 0.36	0.21	0.09, 0.33
	BF_{10}	28.2131	1.05e0-6						
Fiscal-Confidence	Student's t	1.008		537	0.314	0.12	-0.11, 0.35	0.09	-0.08, 0.26
	BF_{10}	0.1575	2.89e0-6						
Fiscal-C-Confidence	Student's t	2.58		539	0.01	0.31	0.07, 0.55	0.22	0.05, 0.39
	BF_{10}	2.4092	1.73e0-7						
Freewill-Confidence	Student's t	4.175		531	< .001	0.54	0.29, 0.80	0.36	0.19, 0.53
	BF_{10}	416.9552	6.84E-10						
Freewill-C-Confidence	Student's t	0.755		545	0.451	0.10	-0.17, 0.38	0.06	-0.10, 0.23
	BF_{10}	0.1256	4.38e0-6						
Gamers-Confidence	Student's t	3.919		535	< .001	0.50	0.25, 0.76	0.34	0.17, 0.51
	BF_{10}	155.5002	2.12e0-9						
Gamers-C-Confidence	Student's t	0.147		541	0.884	0.02	-0.23, 0.27	0.01	-0.16, 0.18
	BF_{10}	0.0965	5.47e0-6						
Morality-Confidence	Student's t	5.792		538	< .001	0.64	0.42, 0.86	0.50	0.32, 0.67
	BF_{10}	782005.8304	3.13E-13						
Morality-C-Confidence	Student's t	1.115		538	0.265	0.15	-0.12, 0.42	0.10	-0.07, 0.27
	BF_{10}	0.1753	2.77e0-6						
Nuclear-Confidence	Student's t	3.514		543	< .001	0.43	0.19, 0.66	0.30	0.13, 0.47
	BF_{10}	36.9024	1.12e0-8						
Nuclear-C-Confidence	Student's t	1.212		533	0.226	0.16	-0.10, 0.43	0.10	-0.07, 0.27
	BF_{10}	0.1965	2.25e0-6						
Politicians-Confidence	Student's t	3.513		535	< .001	0.42	0.19, 0.66	0.30	0.13, 0.47
	BF_{10}	36.9955	9.41e0-9						
Politicians-C-Confidence	Student's t	1.179		541	0.239	0.15	-0.10, 0.40	0.10	-0.07, 0.27
	BF_{10}	0.1879	2.67e0-6						
Professors-Confidence	Student's t	4.136		540	< .001	0.50	0.26, 0.74	0.36	0.18, 0.53
	BF_{10}	356.2412	9.39E-10						
Professors-C-Confidence	Student's t	0.646		536	0.518	0.09	-0.18, 0.35	0.06	-0.11, 0.23
	BF_{10}	0.1179	3.83e0-6						
Referees-Confidence	Student's t	4.376		542	< .001	0.54	0.30, 0.78	0.38	0.20, 0.55
	BF_{10}	934.7601	3.78E-10						
Referees-C-Confidence	Student's t	0.795		534	0.427	0.11	-0.16, 0.38	0.07	-0.10, 0.24
	BF_{10}	0.1308	3.50e0-6						
Struggle-Confidence	Student's t	1.243		541	0.215	0.17	-0.10, 0.44	0.11	-0.06, 0.28
	BF_{10}	0.2024	2.50e0-6						
Struggle-C-Confidence	Student's t	0.353		535	0.724	0.05	-0.21, 0.30	0.03	-0.14, 0.20
	BF_{10}	0.102	4.63e0-6						
Turnout-Confidence	Student's t	2.587		539	0.01	0.31	0.07, 0.54	0.22	0.05, 0.39
	BF_{10}	2.4496	1.77e0-7						
Turnout-C-Confidence	Student's t	2.033		537	0.043	0.26	0.01, 0.52	0.18	0.01, 0.34
	BF_{10}	0.7129	6.24e0-7						

[a] Levene's test is significant (p < .05), suggesting a violation of the assumption of equal variances

Table S17.

Study 4 (scapegoating): trustworthiness measure item-level results (independent samples and Bayesian *t*-tests). *Note:* Bayesian prior used is 0.707 (representing an 80% chance that the effect size is between -2 and 2). "-C-" denotes a manipulative post's matched neutral control.

Trustworthiness		Statistic	±%	df	*p*	M_{diff}	95%CI	Cohen's *d*	95%CI
Manipulative-Trustworthiness	Student's t	-1.888		1076	0.059	-0.15	-0.31, 0.01	-0.12	-0.23, 0.00
	BF_{10}	0.395	8.03E-05						
Neutral-Trustworthiness	Student's t	-0.463		1077	0.643	-0.03	-0.17, 0.10	-0.03	-0.15, 0.09
	BF_{10}	0.0758	4.36E-04						
Discernment-Trustworthiness	Student's t	1.6479		1077	0.1	0.13	-0.02, 0.28	0.10	-0.02, 0.22
	BF_{10}	0.26	1.24E-04						
Fiscal-Trustworthiness	Student's t	-0.1252		537	0.9	-0.02	-0.30, 0.26	-0.01	-0.18, 0.16
	BF_{10}	0.0968	4.84E-06						
Fiscal-C-Trustworthiness	Student's t	-1.1721		539	0.242	-0.16	-0.42, 0.11	-0.10	-0.27, 0.07
	BF_{10}	0.187	2.54E-06						
Freewill-Trustworthiness	Student's t	-1.804		531	0.072	-0.26	-0.54, 0.02	-0.16	-0.33, 0.01
	BF_{10}	0.4682	8.65E-07						
Freewill-C-Trustworthiness	Student's t	1.203		545	0.229	0.16	-0.10, 0.42	0.10	-0.07, 0.27
	BF_{10}	0.1925	2.80E-06						
Gamers-Trustworthiness	Student's t	-0.4895		535	0.625	-0.07	-0.35, 0.21	-0.04	-0.21, 0.13
	BF_{10}	0.1079	4.40E-06						
Gamers-C-Trustworthiness	Student's t	-0.4869		541	0.627	-0.06	-0.32, 0.19	-0.04	-0.21, 0.13
	BF_{10}	0.1072	4.89E-06						
Morality-Trustworthiness	Student's t	-1.3496		538	0.178	-0.20	-0.50, 0.09	-0.12	-0.29, 0.05
	BF_{10}	0.2322	2.06E-06						
Morality-C-Trustworthiness	Student's t	-0.6229		538	0.534	-0.09	-0.36, 0.19	-0.05	-0.22, 0.12
	BF_{10}	0.1157	4.30E-06						
Nuclear-Trustworthiness	Student's t	-1.5917		543	0.112	-0.24	-0.53, 0.06	-0.14	-0.30, 0.03
	BF_{10}	0.3267	1.57E-06						
Nuclear-C-Trustworthiness	Student's t	-1.1136		533	0.266	-0.14	-0.40, 0.11	-0.10	-0.27, 0.07
	BF_{10}	0.1759	2.53E-06						
Politicians-Trustworthiness	Student's t	-1.3501		535	0.178	-0.20	-0.50, 0.09	-0.12	-0.29, 0.05
	BF_{10}	0.2331	1.93E-06						
Politicians-C-Trustworthiness	Student's t	-1.1959		541	0.232	-0.15	-0.40, 0.10	-0.10	-0.27, 0.07
	BF_{10}	0.1917	2.62E-06						
Professors-Trustworthiness	Student's t	-1.9394		540	0.053	-0.27	-0.54, 0.00	-0.17	-0.34, 0.00
	BF_{10}	0.5953	7.59E-07						
Professors-C-Trustworthiness	Student's t	-0.0308		536	0.975	0.00	-0.26, 0.25	0.00	-0.17, 0.17
	BF_{10}	0.0963	4.74E-06						
Referees-Trustworthiness	Student's t	-1.8168		542	0.07	-0.25	-0.51, 0.02	-0.16	-0.32, 0.01
	BF_{10}	0.4745	1.04E-06						
Referees-C-Trustworthiness	Student's t	0.2698		534	0.787	0.03	-0.21, 0.28	0.02	-0.15, 0.19
	BF_{10}	0.0996	4.67E-06						
Struggle-Trustworthiness	Student's t	-2.5026		541	0.013	-0.36	-0.64, -0.08	-0.21	-0.38, -0.05
	BF_{10}	1.9891	2.28E-07						
Struggle-C-Trustworthiness	Student's t	1.605		535	0.109	0.21	-0.05, 0.48	0.14	-0.03, 0.31
	BF_{10}	0.3359	1.32E-06						
Turnout-Trustworthiness	Student's t	1.333		539	0.183	0.20	-0.09, 0.50	0.11	-0.05, 0.28
	BF_{10}	0.227	2.15E-06						
Turnout-C-Trustworthiness	Student's t	0.2388		537	0.811	0.03	-0.22, 0.28	0.02	-0.15, 0.19
	BF_{10}	0.0985	5.01E-06						

[a] Levene's test is significant ($p < .05$), suggesting a violation of the assumption of equal variances

Table S18.

Study 4 (scapegoating): sharing intentions measure item-level results (independent samples and Bayesian *t*-tests). *Note:* Bayesian prior used is 0.707 (representing an 80% chance that the effect size is between -2 and 2). "-C-" denotes a manipulative post's matched neutral control.

**Sharing**		**Statistic**	**±%**	**df**	***p***	**M_{diff}**	**95%CI**	**Cohen's *d***	**95%CI**
Manipulative-Sharing	Student's t	-0.479		1076	0.632	-0.04	-0.21, 0.13	-0.03	-0.15, 0.09
	BF_{10}	0.0764	4.29E-04						
Neutral-Sharing	Student's t	0.758		1077	0.449	0.07	-0.11, 0.25	0.05	-0.07, 0.17
	BF_{10}	0.0906	3.64E-04						
Discernment-Sharing	Student's t	1.834		1077	0.067	0.12	-0.01, 0.25	0.11	-0.01, 0.23
	BF_{10}	0.3579	8.97E-05						
Fiscal-Sharing	Student's t	-0.397		537	0.691	-0.05	-0.32, 0.21	-0.03	-0.20, 0.14
	BF_{10}	0.1038	4.50E-06						
Fiscal-C-Sharing	Student's t	-0.813		539	0.416	-0.12	-0.42, 0.17	-0.07	-0.24, 0.10
	BF_{10}	0.1323	3.66E-06						
Freewill-Sharing	Student's t	-1.153		531	0.25	-0.16	-0.43, 0.11	-0.10	-0.27, 0.07
	BF_{10}	0.1839	2.32E-06						
Freewill-C-Sharing	Student's t	1.997		545	0.046	0.32	0.01, 0.64	0.17	0.00, 0.34
	BF_{10}	0.6607	7.68E-07						
Gamers-Sharing	Student's t	0.653		535	0.514	0.09	-0.19, 0.37	0.06	-0.11, 0.23
	BF_{10}	0.1182	4.00E-06						
Gamers-C-Sharing	Student's t	0.122		541	0.903	0.02	-0.29, 0.33	0.01	-0.16, 0.18
	BF_{10}	0.0962	5.48E-06						
Morality-Sharing	Student's t	-0.632		538	0.528	-0.09	-0.37, 0.19	-0.05	-0.22, 0.11
	BF_{10}	0.1163	4.26E-06						
Morality-C-Sharing	Student's t	-0.106		538	0.916	-0.02	-0.32, 0.29	-0.01	-0.18, 0.16
	BF_{10}	0.0963	5.22E-06						
Nuclear-Sharing	Student's t	-0.755		543	0.451	-0.11	-0.40, 0.18	-0.06	-0.23, 0.10
	BF_{10}	0.1258	4.27E-06						
Nuclear-C-Sharing	Student's t	-0.267		533	0.79	-0.04	-0.35, 0.26	-0.02	-0.19, 0.15
	BF_{10}	0.0996	4.61E-06						
Politicians-Sharing	Student's t	-1.45		535	0.148	-0.23	-0.55, 0.08	-0.13	-0.29, 0.04
	BF_{10}	0.2669	1.68E-06						
Politicians-C-Sharing	Student's t	0.447		541	0.655	0.07	-0.25, 0.40	0.04	-0.13, 0.21
	BF_{10}	0.1053	4.91E-06						
Professors-Sharing	Student's t	-0.216		540	0.829	-0.03	-0.31, 0.24	-0.02	-0.19, 0.15
	BF_{10}	0.098	5.07E-06						
Professors-C-Sharing	Student's t	0.125		536	0.901	0.02	-0.30, 0.34	0.01	-0.16, 0.18
	BF_{10}	0.097	4.71E-06						
Referees-Sharing	Student's t	-0.645		542	0.519	-0.09	-0.38, 0.19	-0.06	-0.22, 0.11
	BF_{10}	0.1169	4.53E-06						
Referees-C-Sharing	Student's t	1.383		534	0.167	0.21	-0.09, 0.50	0.12	-0.05, 0.29
	BF_{10}	0.2435	1.82E-06						
Struggle-Sharing	Student's t	-1.262		541	0.208	-0.17	-0.44, 0.09	-0.11	-0.28, 0.06
	BF_{10}	0.2073	2.44E-06						
Struggle-C-Sharing	Student's t	1.978		535	0.048	0.33	0.00, 0.65	0.17	0.00, 0.34
	BF_{10}	0.6424	6.66E-07						
Turnout-Sharing	Student's t	1.635		539	0.103	0.25	-0.05, 0.54	0.14	-0.03, 0.31
	BF_{10}	0.3509	1.36E-06						
Turnout-C-Sharing	Student's t	-0.674		537	0.5	-0.11	-0.42, 0.21	-0.06	-0.23, 0.11
	BF_{10}	0.1196	4.08E-06						

[a] Levene's test is significant ($p < .05$), suggesting a violation of the assumption of equal variances

Table S19.

Study 5 (ad hominem): technique recognition measure item-level results (independent samples and Bayesian *t*-tests). *Note:* Bayesian prior used is 0.707 (representing an 80% chance that the effect size is between -2 and 2). "-C-" denotes a manipulative post's matched neutral control.

Ad hominem		Statistic	±%	df	*p*	M_{diff}	95%CI	Cohen's *d*	95%CI
Manipulative-AdHominem	Student's t	3.1577		1079	0.002	0.23	0.09, 0.37	0.19	0.07, 0.31
	BF_{10}	9.1358	3.37e0-6						
Neutral-AdHominem	Student's t	-7.3523		1079	< .001	-0.54	-0.68, -0.39	-0.45	-0.57, -0.32
	BF_{10}	1.43E+10	1.52E-15						
Discernment-AdHominem	Student's t	7.3603		1079	< .001	0.76	0.56, 0.96	0.45	0.33, 0.57
	BF_{10}	1.51E+10	1.43E-15						
Academia-AdHominem	Student's t	4.4393		536	< .001	0.58	0.32, 0.84	0.38	0.21, 0.55
	BF_{10}	1220.0418	2.50E-10						
Academia-C-AdHominem	Student's t	-4.739		543	< .001	-0.75	-1.06, -0.44	-0.41	-0.58, -0.23
	BF_{10}	4456.0114	7.49E-11						
Crime-AdHominem	Student's t	-2.5525		542	0.011	-0.39	-0.70, -0.09	-0.22	-0.39, -0.05
	BF_{10}	2.2459	2.01e0-7						
Crime-C-AdHominem	Student's t	-5.4475		537	< .001	-0.87	-1.18, -0.55	-0.47	-0.64, -0.30
	BF_{10}	129952.599	1.97E-12						
Dumped-AdHominem	Student's t	1.2617		542	0.208	0.17	-0.10, 0.44	0.11	-0.06, 0.28
	BF_{10}	0.2071	2.47e0-6						
Dumped-C-AdHominem	Student's t	-2.4763		537	0.014	-0.36	-0.65, -0.08	-0.21	-0.38, -0.04
	BF_{10}	1.8745	2.20e0-7						
Education-AdHominem	Student's t	2.489		537	0.013	0.34	0.07, 0.61	0.21	0.04, 0.38
	BF_{10}	1.9305	2.18e0-7						
Education-C-AdHominem	Student's t	-3.3545		542	< .001	-0.48	-0.76, -0.20	-0.29	-0.46, -0.12
	BF_{10}	21.8608	1.90e0-8						
Fired-AdHominem	Student's t	1.8518		540	0.065	0.25	-0.02, 0.51	0.16	-0.01, 0.33
	BF_{10}	0.507	8.89e0-7						
Fired-C-AdHominem	Student's t	-3.6584		539	< .001	-0.57	-0.88, -0.26	-0.32	-0.49, -0.14
	BF_{10}	60.6879	5.95e0-9						
Freud-AdHominem	Student's t	1.1091		540	0.268	0.16	-0.12, 0.44	0.10	-0.07, 0.26
	BF_{10}	0.1741	2.83e0-6						
Freud-C-AdHominem	Student's t	-4.1693		539	< .001	-0.67	-0.99, -0.36	-0.36	-0.53, -0.19
	BF_{10}	406.0377	8.41E-10						
Heartland-AdHominem	Student's t	3.4483		540	< .001	0.45	0.19, 0.71	0.30	0.13, 0.47
	BF_{10}	29.7023	1.32e0-8						
Heartland-C-AdHominem	Student's t	-0.6682		539	0.504	-0.10	-0.40, 0.20	-0.06	-0.23, 0.11
	BF_{10}	0.1189	4.25e0-6						
Marriage-AdHominem	Student's t	2.8661		543	0.004	0.40	0.13, 0.68	0.25	0.08, 0.41
	BF_{10}	5.0845	8.84e0-8						
Marriage-C-AdHominem	Student's t	-2.835		536	0.005	-0.44	-0.75, -0.14	-0.24	-0.41, -0.07
	BF_{10}	4.6905	8.46e0-8						
Presidential-AdHominem	Student's t	3.0642		535	0.002	0.46	0.17, 0.76	0.26	0.09, 0.44
	BF_{10}	8.9888	4.18e0-8						
Presidential-C-AdHominem	Student's t	-1.4597		544	0.145	-0.22	-0.51, 0.08	-0.13	-0.29, 0.04
	BF_{10}	0.2687	1.93e0-6						
Twitterbio-AdHominem	Student's t	0.0518		539	0.959	0.01	-0.28, 0.30	0.00	-0.16, 0.17
	BF_{10}	0.0959	5.21e0-6						
Twitterbio-C-AdHominem	Student's t	-4.0019		540	< .001	-0.62	-0.92, -0.31	-0.34	-0.51, -0.17
	BF_{10}	212.0356	1.69e0-9						

[a] Levene's test is significant ($p < .05$), suggesting a violation of the assumption of equal variances

Table S20.

Study 5 (ad hominem): confidence measure item-level results (independent samples and Bayesian *t*-tests). *Note:* Bayesian prior used is 0.707 (representing an 80% chance that the effect size is between -2 and 2). "-C-" denotes a manipulative post's matched neutral control.

Confidence		Statistic	±%	df	*p*	M_{diff}	95%CI	Cohen's *d*	95%CI
Manipulative-Confidence	Student's t	4.0087		1079	< .001	0.31	0.16, 0.46	0.24	0.12, 0.36
	BF_{10}	179.0774	1.63e0-7						
Neutral-Confidence	Student's t	5.9184		1079	< .001	0.44	0.30, 0.59	0.36	0.24, 0.48
	BF_{10}	1.69E+06	1.48E-11						
Discernment-Confidence	Student's t	-2.1449		1079	0.032	-0.14	-0.26, -0.01	-0.13	-0.25, -0.01
	BF_{10}	0.6566	4.92e0-5						
Academia-Confidence	Student's t	2.9467		536	0.003	0.40	0.13, 0.66	0.25	0.08, 0.42
	BF_{10}	6.3997	6.08e0-8						
Academia-C-Confidence	Student's t	4.4111		543	< .001	0.64	0.36, 0.93	0.38	0.21, 0.55
	BF_{10}	1082.9442	3.26E-10						
Crime-Confidence	Student's t	0.3874		542	0.699	0.05	-0.20, 0.29	0.03	-0.14, 0.20
	BF_{10}	0.1027	5.13e0-6						
Crime-C-Confidence	Student's t	4.4962		537	< .001	0.65	0.36, 0.93	0.39	0.22, 0.56
	BF_{10}	1550.3363	2.00E-10						
Dumped-Confidence	Student's t	2.328		542	0.02	0.31	0.05, 0.58	0.20	0.03, 0.37
	BF_{10}	1.3232	3.53e0-7						
Dumped-C-Confidence	Student's t	1.8418		537	0.066	0.26	-0.02, 0.53	0.16	-0.01, 0.33
	BF_{10}	0.4985	8.87e0-7						
Education-Confidence	Student's t	2.2162		537	0.027	0.29	0.03, 0.56	0.19	0.02, 0.36
	BF_{10}	1.0387	4.19e0-7						
Education-C-Confidence	Student's t	4.4996		542	< .001	0.67	0.38, 0.96	0.39	0.21, 0.56
	BF_{10}	1571.25	2.21E-10						
Fired-Confidence	Student's t	0.8065		540	0.42	0.11	-0.16, 0.38	0.07	-0.10, 0.24
	BF_{10}	0.1316	3.68e0-6						
Fired-C-Confidence	Student's t	3.0262		539	0.003	0.41	0.14, 0.67	0.26	0.09, 0.43
	BF_{10}	8.0322	4.93e0-8						
Freud-Confidence	Student's t	0.784		540	0.433	0.11	-0.17, 0.39	0.07	-0.10, 0.24
	BF_{10}	0.1291	3.88e0-6						
Freud-C-Confidence	Student's t	3.2223		539	0.001	0.43	0.17, 0.69	0.28	0.11, 0.45
	BF_{10}	14.4419	2.74e0-8						
Heartland-Confidence	Student's t	4.1071		540	< .001	0.55	0.29, 0.82	0.35	0.18, 0.52
	BF_{10}	317.8889	1.11e0-9						
Heartland-C-Confidence	Student's t	0.0574		539	0.954	0.01	-0.25, 0.27	0.00	-0.16, 0.17
	BF_{10}	0.0958	5.33e0-6						
Marriage-Confidence	Student's t	2.1216		543	0.034	0.29	0.02, 0.55	0.18	0.01, 0.35
	BF_{10}	0.8479	5.76e0-7						
Marriage-C-Confidence	Student's t	2.568		536	0.01	0.36	0.09, 0.64	0.22	0.05, 0.39
	BF_{10}	2.3423	1.75e0-7						
Presidential-Confidence	Student's t	3.9868		535	< .001	0.56	0.28, 0.84	0.34	0.17, 0.52
	BF_{10}	200.6357	1.62e0-9						
Presidential-C-Confidence	Student's t	0.7912		544	0.429	0.10	-0.15, 0.36	0.07	-0.10, 0.24
	BF_{10}	0.1293	4.16e0-6						
Twitterbio-Confidence	Student's t	1.5773		539	0.115	0.21	-0.05, 0.47	0.14	-0.03, 0.30
	BF_{10}	0.321	1.46e0-6						
Twitterbio-C-Confidence	Student's t	4.3092		540	< .001	0.62	0.34, 0.90	0.37	0.20, 0.54
	BF_{10}	711.8482	4.78E-10						

[a] Levene's test is significant ($p < .05$), suggesting a violation of the assumption of equal variances

Table S21.

Study 5 (ad hominem): trustworthiness measure item-level results (independent samples and Bayesian *t*-tests). *Note:* Bayesian prior used is 0.707 (representing an 80% chance that the effect size is between -2 and 2). "-C-" denotes a manipulative post's matched neutral control.

Trustworthiness		Statistic	±%	df	*p*	M_{diff}	95%CI	Cohen's *d*	95%CI
Manipulative-Trustworthiness	Student's t	-1.752		1079	0.08	-0.13	-0.28, 0.02	-0.11	-0.23, 0.01
	BF_{10}	0.3094	1.06E-04						
Neutral-Trustworthiness	Student's t	1.411		1079	0.158	0.10	-0.04, 0.23	0.09	-0.03, 0.21
	BF_{10}	0.1819	1.82E-04						
Discernment-Trustworthiness	Student's t	3.145		1079	0.002	0.22	0.08, 0.36	0.19	0.07, 0.31
	BF_{10}	8.7818	3.50E-06						
Academia-Trustworthiness	Student's t	-1.885		536	0.06	-0.25	-0.50, 0.01	-0.16	-0.33, 0.01
	BF_{10}	0.5389	8.18E-07						
Academia-C-Trustworthiness	Student's t	0.882		543	0.378	0.12	-0.15, 0.40	0.08	-0.09, 0.24
	BF_{10}	0.1394	3.78E-06						
Crime-Trustworthiness	Student's t	-1.087		542	0.277	-0.14	-0.40, 0.11	-0.09	-0.26, 0.08
	BF_{10}	0.1697	3.02E-06						
Crime-C-Trustworthiness	Student's t	1.697		537	0.09	0.22	-0.04, 0.48	0.15	-0.02, 0.32
	BF_{10}	0.3887	1.18E-06						
Dumped-Trustworthiness	Student's t	-0.63		542	0.529	-0.08	-0.34, 0.18	-0.05	-0.22, 0.11
	BF_{10}	0.1158	4.55E-06						
Dumped-C-Trustworthiness	Student's t	1.929		537	0.054	0.24	0.00, 0.49	0.17	0.00, 0.34
	BF_{10}	0.5844	7.50E-07						
Education-Trustworthiness	Student's t	-1.37		537	0.171	-0.18	-0.44, 0.08	-0.12	-0.29, 0.05
	BF_{10}	0.2388	1.97E-06						
Education-C-Trustworthiness	Student's t	1.438		542	0.151	0.17	-0.06, 0.40	0.12	-0.05, 0.29
	BF_{10}	0.261	1.95E-06						
Fired-Trustworthiness	Student's t	-1.414		540	0.158	-0.17	-0.41, 0.07	-0.12	-0.29, 0.05
	BF_{10}	0.2536	1.84E-06						
Fired-C-Trustworthiness	Student's t	-0.676		539	0.499	-0.10	-0.38, 0.18	-0.06	-0.23, 0.11
	BF_{10}	0.1197	4.10E-06						
Freud-Trustworthiness	Student's t	-0.452		540	0.652	-0.06	-0.32, 0.20	-0.04	-0.21, 0.13
	BF_{10}	0.1057	4.79E-06						
Freud-C-Trustworthiness	Student's t	0.919		539	0.358	0.12	-0.14, 0.38	0.08	-0.09, 0.25
	BF_{10}	0.1444	3.44E-06						
Heartland-Trustworthiness	Student's t	-1.54		540	0.124	-0.20	-0.46, 0.06	-0.13	-0.30, 0.04
	BF_{10}	0.303	1.61E-06						
Heartland-C-Trustworthiness	Student's t	-1.743		539	0.082	-0.23	-0.49, 0.03	-0.15	-0.32, 0.02
	BF_{10}	0.419	1.13E-06						
Marriage-Trustworthiness	Student's t	-1.172		543	0.242	-0.17	-0.45, 0.11	-0.10	-0.27, 0.07
	BF_{10}	0.1859	2.83E-06						
Marriage-C-Trustworthiness	Student's t	-0.14		536	0.888	-0.02	-0.29, 0.25	-0.01	-0.18, 0.16
	BF_{10}	0.0968	5.02E-06						
Presidential-Trustworthiness	Student's t	-2.146		535	0.032	-0.29	-0.55, -0.02	-0.19	-0.36, -0.02
	BF_{10}	0.8981	4.70E-07						
Presidential-C-Trustworthiness	Student's t	0.85		544	0.396	0.11	-0.15, 0.37	0.07	-0.10, 0.24
	BF_{10}	0.1355	3.96E-06						
Twitterbio-Trustworthiness	Student's t	0.307		539	0.759	0.04	-0.22, 0.30	0.03	-0.14, 0.20
	BF_{10}	0.1003	4.97E-06						
Twitterbio-C-Trustworthiness	Student's t	1.869		540	0.062	0.26	-0.01, 0.53	0.16	-0.01, 0.33
	BF_{10}	0.5219	9.05E-07						

[a] Levene's test is significant ($p < .05$), suggesting a violation of the assumption of equal variances

Table S22.

Study 5 (ad hominem): sharing measure item-level results (independent samples and Bayesian *t*-tests). *Note:* Bayesian prior used is 0.707 (representing an 80% chance that the effect size is between -2 and 2). “-C-“ denotes a manipulative post’s matched neutral control.

Sharing		Statistic	±%	df	*p*	M_{diff}	95%CI	Cohen's *d*	95%CI
Manipulative-Sharing	Student's t	-1.8785		1079	0.061	-0.15	-0.30, 0.01	-0.11	-0.23, 0.01
	BF_{10}	0.3876	8.41E-05						
Neutral-Sharing	Student's t	0.6023		1079	0.547	0.05	-0.12, 0.22	0.04	-0.08, 0.16
	BF_{10}	0.0815	4.13E-04						
Discernment-Sharing	Student's t	3.1907		1079	0.001	0.20	0.08, 0.32	0.19	0.07, 0.31
	BF_{10}	10.1227	3.03E-06						
Academia-Sharing	Student's t	-2.0655		536	0.039	-0.25	-0.49, -0.01	-0.18	-0.35, -0.01
	BF_{10}	0.761	5.69E-07						
Academia-C-Sharing	Student's t	-0.1042		543	0.917	-0.02	-0.36, 0.32	-0.01	-0.18, 0.16
	BF_{10}	0.0959	5.59E-06						
Crime-Sharing	Student's t	-1.7067		542	0.088	-0.24	-0.51, 0.04	-0.15	-0.31, 0.02
	BF_{10}	0.3934	1.25E-06						
Crime-C-Sharing	Student's t	1.9234		537	0.055	0.28	-0.01, 0.56	0.17	0.00, 0.34
	BF_{10}	0.578	7.75E-07						
Dumped-Sharing	Student's t	-0.817		542	0.414	-0.10	-0.34, 0.14	-0.07	-0.24, 0.10
	BF_{10}	0.1321	3.96E-06						
Dumped-C-Sharing	Student's t	0.1199		537	0.905	0.02	-0.30, 0.34	0.01	-0.16, 0.18
	BF_{10}	0.0966	5.00E-06						
Education-Sharing	Student's t	-1.8818		537	0.06	-0.24	-0.49, 0.01	-0.16	-0.33, 0.01
	BF_{10}	0.5353	8.41E-07						
Education-C-Sharing	Student's t	1.4028		542	0.161	0.22	-0.09, 0.53	0.12	-0.05, 0.29
	BF_{10}	0.2485	2.06E-06						
Fired-Sharing	Student's t	-0.5651		540	0.572	-0.07	-0.32, 0.18	-0.05	-0.22, 0.12
	BF_{10}	0.112	4.36E-06						
Fired-C-Sharing	Student's t	-0.8106		539	0.418	-0.13	-0.45, 0.19	-0.07	-0.24, 0.10
	BF_{10}	0.1319	3.70E-06						
Freud-Sharing	Student's t	-0.5985		540	0.55	-0.08	-0.33, 0.18	-0.05	-0.22, 0.12
	BF_{10}	0.1139	4.43E-06						
Freud-C-Sharing	Student's t	0.6405		539	0.522	0.09	-0.19, 0.38	0.06	-0.11, 0.22
	BF_{10}	0.1169	4.30E-06						
Heartland-Sharing	Student's t	-1.1391		540	0.255	-0.15	-0.40, 0.11	-0.10	-0.27, 0.07
	BF_{10}	0.1798	2.78E-06						
Heartland-C-Sharing	Student's t	-1.4622		539	0.144	-0.19	-0.45, 0.07	-0.13	-0.29, 0.04
	BF_{10}	0.2706	1.79E-06						
Marriage-Sharing	Student's t	-1.3292		543	0.184	-0.18	-0.45, 0.09	-0.11	-0.28, 0.05
	BF_{10}	0.2252	2.31E-06						
Marriage-C-Sharing	Student's t	-0.2148		536	0.83	-0.03	-0.35, 0.28	-0.02	-0.19, 0.15
	BF_{10}	0.0981	4.95E-06						
Presidential-Sharing	Student's t	-1.7379		535	0.083	-0.23	-0.49, 0.03	-0.15	-0.32, 0.02
	BF_{10}	0.4166	1.05E-06						
Presidential-C-Sharing	Student's t	0.0989		544	0.921	0.01	-0.26, 0.29	0.01	-0.16, 0.18
	BF_{10}	0.0958	5.70E-06						
Twitterbio-Sharing	Student's t	0.6514		539	0.515	0.08	-0.17, 0.34	0.06	-0.11, 0.22
	BF_{10}	0.1178	4.20E-06						
Twitterbio-C-Sharing	Student's t	0.3627		540	0.717	0.05	-0.24, 0.34	0.03	-0.14, 0.20
	BF_{10}	0.1019	5.04E-06						

[a] Levene's test is significant ($p < .05$), suggesting a violation of the assumption of equal variances

Table S23.

Study 1 (emotional language): linear regression per outcome variable, with covariates. Significant predictors are marked in bold.

	Technique recognition				*Confidence*				*Trustworthiness*				*Sharing*			
**Predictor**	**b**	**SE**	**t**	***p***	**b**	**SE**	**t**	***p***	**b**	**SE**	**t**	***p***	**b**	**SE**	**t**	***p***
Intercept [a]	1.6762	0.441	3.805	< .001	0.45661	0.29	1.577	0.115	0.47679	0.369	1.2911	0.197	0.16702	0.353	0.473	0.636
Condition:																
Inoculation – Control	0.710	0.083	8.594	**< .001**	0.314	0.054	5.791	**< .001**	0.289	0.069	4.1789	**< .001**	0.243	0.066	3.667	**< .001**
Gender	0.313	0.086	3.639	**< .001**	0.016	0.057	0.279	0.78	0.156	0.072	2.1661	**0.031**	0.053	0.069	0.775	0.439
Age	-0.022	0.032	-0.7	0.485	-0.005	0.021	-0.27	0.791	0.008	0.026	0.2994	0.765	0.017	0.025	0.686	0.493
Education	-0.087	0.047	-1.85	0.064	0.030	0.031	0.972	0.332	-0.043	0.04	-1.085	0.278	0.004	0.038	0.109	0.913
Political-Ideology	-0.034	0.025	-1.37	0.171	-0.006	0.016	-0.4	0.692	-0.056	0.021	-2.703	**0.007**	-0.054	0.02	-2.72	**0.007**
News-Check	0.123	0.05	2.458	**0.014**	0.013	0.033	0.399	0.69	0.115	0.042	2.7359	**0.006**	0.088	0.04	2.198	**0.028**
Social-Media-Use	-0.031	0.045	-0.69	0.493	0.004	0.029	0.121	0.904	-0.027	0.037	-0.725	0.469	0.018	0.036	0.511	0.61
Populism	-0.025	0.076	-0.34	0.738	-0.052	0.05	-1.04	0.298	-0.006	0.064	-0.087	0.931	-0.116	0.061	-1.92	0.056
Bullshit-Receptivity	-0.261	0.05	-5.29	**< .001**	-0.110	0.033	-3.38	**< .001**	-0.076	0.042	-1.842	0.066	0.036	0.04	0.898	0.369
Conspiracy-Belief	-0.255	0.232	-1.1	0.273	0.086	0.153	0.565	0.572	-0.185	0.195	-0.951	0.342	-0.125	0.186	-0.67	0.502
Analytical-Thinking	0.111	0.039	2.864	**0.004**	0.046	0.025	1.809	0.071	0.101	0.032	3.1221	**0.002**	-0.012	0.031	-0.39	0.70
Numerical-Thinking	0.173	0.045	3.87	**< .001**	-0.004	0.029	-0.15	0.884	0.164	0.038	4.3753	**< .001**	0.101	0.036	2.803	**0.005**

[a] Represents reference level

Table S24.

Study 2 (incoherence): linear regression per outcome variable, with covariates. Significant predictors are marked in bold.

	Technique recognition				*Confidence*				*Trustworthiness*				*Sharing*			
**Predictor**	**b**	**SE**	**t**	***p***	**b**	**SE**	**t**	***p***	**b**	**SE**	**t**	***p***	**b**	**SE**	**t**	***p***
Intercept [a]	2.007	0.485	4.143	< .001	0.35028	0.278	1.2586	0.208	2.11531	0.391	5.4068	< .001	1.02273	0.331	3.086	0.002
Condition:																
Control – Inoculation	-0.881	0.088	-10.04	**< .001**	-0.190	0.051	-3.757	**< .001**	-0.207	0.071	-2.922	**0.004**	-0.086	0.06	-1.44	0.151
Gender	-0.074	0.091	-0.818	0.413	-0.023	0.052	-0.445	0.656	-0.116	0.073	-1.583	0.114	-0.130	0.062	-2.1	**0.036**
Age	-0.073	0.035	-2.063	0.039	0.002	0.02	0.0966	0.923	-0.003	0.029	-0.101	0.92	0.004	0.024	0.181	0.856
Education	-0.063	0.051	-1.23	0.219	-0.010	0.029	-0.335	0.738	-0.084	0.041	-2.036	**0.042**	-0.114	0.035	-3.27	**0.001**
Political-Ideology	-0.112	0.027	-4.121	**< .001**	-0.006	0.016	-0.379	0.704	-0.192	0.022	-8.755	**< .001**	-0.098	0.019	-5.25	**< .001**
News-Check	0.055	0.054	1.003	0.316	0.027	0.031	0.8666	0.386	0.062	0.044	1.4197	0.156	0.062	0.037	1.668	0.096
Social-Media-Use	-0.016	0.047	-0.342	0.733	-0.044	0.027	-1.655	0.098	-0.025	0.038	-0.663	0.508	-0.035	0.032	-1.11	0.267
Populism	0.056	0.081	0.687	0.492	0.010	0.047	0.2172	0.828	0.001	0.066	0.0184	0.985	-0.042	0.056	-0.76	0.45
Bullshit-Receptivity	-0.198	0.053	-3.726	**< .001**	-0.020	0.031	-0.666	0.506	-0.106	0.043	-2.457	**0.014**	0.102	0.036	2.788	**0.005**
Conspiracy-Belief	-0.085	0.237	-0.356	0.722	-0.044	0.136	-0.321	0.749	-0.384	0.192	-2.003	**0.045**	-0.141	0.162	-0.87	0.385
Analytical-Thinking	0.067	0.05	1.33	0.184	-0.041	0.029	-1.407	0.16	0.035	0.041	0.8691	0.385	-0.007	0.034	-0.21	0.833
Numerical-Thinking	0.224	0.05	4.495	**< .001**	0.016	0.029	0.5459	0.585	0.187	0.04	4.6594	**< .001**	0.117	0.034	3.436	**< .001**

[a] Represents reference level

Table S25.

Study 3 (false dichotomies): linear regression per outcome variable, with covariates. Significant predictors are marked in bold.

	Technique recognition				*Confidence*				*Trustworthiness*				*Sharing*			
**Predictor**	**b**	**SE**	**t**	***p***	**b**	**SE**	**t**	***p***	**b**	**SE**	**t**	***p***	**b**	**SE**	**t**	***p***
Intercept [a]	3.15614	0.487	6.4809	< .001	0.27272	0.315	0.86459	0.387	1.75007	0.374	4.677	< .001	0.6304	0.342	1.841	0.066
Condition:																
Control – Inoculation	-1.164	0.089	-13.137	**< .001**	-0.236	0.057	-4.1193	**< .001**	-0.397	0.068	-5.83	**< .001**	-0.242	0.062	-3.89	**< .001**
Gender	0.158	0.093	1.7009	0.089	0.062	0.06	1.03602	0.3	0.056	0.071	0.788	0.431	0.020	0.065	0.31	0.757
Age	-0.065	0.035	-1.8411	0.066	-0.053	0.023	-2.3236	**0.02**	0.004	0.027	0.14	0.889	0.032	0.025	1.279	0.201
Education	-0.067	0.052	-1.3037	0.193	0.044	0.034	1.30249	0.193	-0.055	0.04	-1.39	0.166	-0.082	0.036	-2.26	**0.024**
Political-Ideology	-0.098	0.027	-3.6329	**< .001**	-9.47e–4	0.017	-0.0544	0.957	-0.081	0.021	-3.92	**< .001**	-0.054	0.019	-2.86	**0.004**
News-Check	0.039	0.053	0.7281	0.467	0.005	0.035	0.14324	0.886	0.031	0.041	0.763	0.445	0.016	0.038	0.428	0.668
Social-Media-Use	0.052	0.047	1.1044	0.27	-0.020	0.03	-0.6498	0.516	0.062	0.036	1.712	0.087	0.045	0.033	1.378	0.168
Populism	-0.007	0.083	-0.0852	0.932	0.044	0.053	0.82315	0.411	-0.094	0.063	-1.48	0.14	0.050	0.058	0.867	0.386
Bullshit-Receptivity	-0.385	0.053	-7.3352	**< .001**	0.009	0.034	0.27225	0.785	-0.123	0.04	-3.05	**0.002**	0.025	0.037	0.665	0.506
Conspiracy-Belief	-0.775	0.234	-3.3063	**< .001**	-0.242	0.152	-1.5954	0.111	-0.502	0.18	-2.79	**0.005**	-0.423	0.165	-2.57	**0.01**
Analytical-Thinking	0.133	0.051	2.6186	0.009	0.000	0.033	0.0055	0.996	0.105	0.039	2.691	**0.007**	0.029	0.036	0.823	0.411
Numerical-Thinking	0.287	0.051	5.6051	**< .001**	0.018	0.033	0.53452	0.593	0.096	0.039	2.446	**0.015**	0.027	0.036	0.761	0.447

[a] Represents reference level

Table S26.

Study 4 (scapegoating): linear regression per outcome variable, with covariates. Significant predictors are marked in bold.

	Technique recognition				*Confidence*				*Trustworthiness*				*Sharing*			
**Predictor**	**b**	**SE**	**t**	***p***	**b**	**SE**	**t**	***p***	**b**	**SE**	**t**	***p***	**b**	**SE**	**t**	***p***
Intercept [a]	3.2073	0.515	6.231	< .001	0.66618	0.347	1.921	0.055	1.65941	0.399	4.1637	< .001	0.87836	0.344	2.552	0.01
Condition:																
Control – Inoculation	-0.420	0.1	-4.22	**< .001**	-0.231	0.067	-3.44	**< .001**	-0.091	0.077	-1.183	0.237	-0.119	0.067	-1.79	0.08
Gender	0.369	0.105	3.516	**< .001**	-0.043	0.071	-0.6	0.546	0.240	0.081	2.9528	**0.003**	0.056	0.07	0.796	0.43
Age	0.079	0.041	1.949	0.052	-0.010	0.027	-0.35	0.726	0.054	0.032	1.7279	0.084	0.030	0.027	1.102	0.27
Education	-0.299	0.057	-5.27	**< .001**	0.045	0.038	1.181	0.238	-0.163	0.044	-3.714	< .001	-0.061	0.038	-1.61	0.11
Political-Ideology	-0.045	0.03	-1.53	0.126	0.009	0.02	0.445	0.656	-0.022	0.023	-0.96	0.337	-0.018	0.02	-0.92	0.36
News-Check	-0.020	0.066	-0.3	0.766	-0.064	0.045	-1.43	0.153	0.072	0.051	1.408	0.159	-0.009	0.044	-0.2	0.84
Social-Media-Use	0.088	0.05	1.761	0.079	-0.010	0.034	-0.3	0.763	0.002	0.039	0.0619	0.951	0.027	0.033	0.823	0.41
Populism	0.238	0.092	2.575	**0.01**	-0.032	0.062	-0.52	0.607	0.009	0.072	0.1232	0.902	-0.094	0.062	-1.53	0.13
Bullshit-Receptivity	-0.312	0.058	-5.34	**< .001**	0.022	0.039	0.552	0.581	-0.111	0.045	-2.453	**0.014**	0.033	0.039	0.836	0.40
Conspiracy-Belief	-1.224	0.274	-4.47	**< .001**	-0.104	0.184	-0.56	0.573	-0.821	0.212	-3.878	**< .001**	-0.130	0.183	-0.71	0.48
Analytical-Thinking	0.047	0.056	0.824	0.41	-0.033	0.038	-0.88	0.379	0.035	0.044	0.8015	0.423	0.040	0.038	1.065	0.29
Numerical-Thinking	0.270	0.057	4.749	**< .001**	0.016	0.038	0.406	0.685	0.156	0.044	3.542	**< .001**	0.058	0.038	1.516	0.13

[a] Represents reference level

Table S27.

Study 5 (ad hominem): linear regression per outcome variable, with covariates. Significant predictors are marked in bold.

	Technique recognition				*Confidence*				*Trustworthiness*				*Sharing*			
**Predictor**	**b**	**SE**	**t**	***p***	**b**	**SE**	**t**	***p***	**b**	**SE**	**t**	***p***	**b**	**SE**	**t**	***p***
Intercept [a]	3.3013	0.517	6.384	< .001	0.7233	0.346	2.09	0.04	0.7872	0.373	2.109	0.035	0.00621	0.335	0.0185	0.99
Condition:																
Control – Inoculation	-0.724	0.096	-7.51	**< .001**	0.163	0.065	2.521	**0.01**	-0.184	0.07	-2.65	**0.008**	-0.170	0.062	-2.724	**0.01**
Gender	0.257	0.1	2.576	**0.01**	0.062	0.067	0.925	0.36	0.194	0.072	2.702	**0.007**	0.062	0.065	0.9672	0.33
Age	-0.094	0.042	-2.25	**0.024**	-0.048	0.028	-1.73	0.09	0.043	0.03	1.429	0.153	0.050	0.027	1.8496	0.07
Education	-0.094	0.055	-1.69	0.091	-0.038	0.037	-1.02	0.31	0.010	0.04	0.245	0.807	-0.026	0.036	-0.726	0.47
Political-Ideology	-0.101	0.03	-3.42	**< .001**	-0.031	0.02	-1.55	0.12	-0.071	0.021	-3.34	**< .001**	-0.034	0.019	-1.782	0.08
News-Check	0.120	0.056	2.134	**0.033**	0.031	0.038	0.83	0.41	0.061	0.041	1.495	0.135	0.083	0.036	2.287	**0.02**
Social-Media-Use	0.091	0.051	1.782	0.075	-0.022	0.034	-0.64	0.52	0.079	0.037	2.141	**0.033**	0.103	0.033	3.1179	**0.002**
Populism	0.020	0.092	0.219	0.827	-0.098	0.061	-1.59	0.11	0.050	0.066	0.759	0.448	0.020	0.059	0.3322	0.74
Bullshit-Receptivity	-0.464	0.057	-8.13	**< .001**	-0.019	0.038	-0.49	0.63	-0.155	0.041	-3.77	**< .001**	0.042	0.037	1.142	0.25
Conspiracy-Belief	-0.630	0.272	-2.32	**0.021**	0.116	0.182	0.64	0.52	-0.433	0.196	-2.21	**0.027**	-0.396	0.176	-2.251	**0.03**
Analytical-Thinking	0.084	0.055	1.527	0.127	-0.015	0.037	-0.41	0.68	0.015	0.04	0.375	0.708	-0.063	0.036	-1.77	0.08
Numerical-Thinking	0.243	0.054	4.53	**< .001**	0.051	0.036	1.412	0.16	0.114	0.039	2.929	**0.003**	0.066	0.035	1.9001	0.06

[a] Represents reference level

Table S28.

Study 1 (emotional language): linear regression at the rating level for technique recognition, trustworthiness discernment and sharing discernment, with robust standard errors clustered on study participants and stimuli (manipulative vs neutral). Outcome variable of interest is "discernmentxinoculation".

**Variables**	**Technique recognition**	**Trustworthiness discernment**	**Sharing discernment**
discernment	-1.336***	0.786***	0.322**
	(0.206)	(0.178)	(0.130)
inoculation	0.649***	-0.00954	0.0432
	(0.150)	(0.0942)	(0.102)
discernmentxinoculation	-0.635***	0.280***	0.162*
	(0.170)	(0.0973)	(0.0946)
Constant	4.868***	3.331***	2.546***
	(0.140)	(0.127)	(0.0960)
Observations	11,018	11,018	11,018
R-squared	0.187	0.078	0.012

Robust standard errors in parentheses
*** p<0.01, ** p<0.05, * p<0.1

Table S29.

Study 2 (incoherence): linear regression at the rating level for technique recognition, trustworthiness discernment and sharing discernment, with robust standard errors clustered on study participants and stimuli (manipulative vs neutral). Outcome variable of interest is "discernmentxinoculation".

**Variables**	**Technique recognition**	**Trustworthiness discernment**	**Sharing discernment**
discernment	-0.796***	1.114***	0.553***
	(0.208)	(0.263)	(0.149)
inoculation	1.426***	-0.329**	-0.0930
	(0.125)	(0.132)	(0.0774)
discernmentxinoculation	-0.834***	0.215	0.135*
	(0.172)	(0.156)	(0.0817)
Constant	3.664***	2.867***	2.025***
	(0.152)	(0.171)	(0.0794)
Observations	11,141	11,141	11,141
R-squared	0.140	0.113	0.031

Robust standard errors in parentheses
*** p<0.01, ** p<0.05, * p<0.1

Table S30.

Study 3 (false dichotomies): linear regression at the rating level for technique recognition, trustworthiness discernment and sharing discernment, with robust standard errors clustered on study participants and stimuli (manipulative vs neutral). Outcome variable of interest is "discernmentxinoculation".

Variables	Technique recognition	Trustworthiness discernment	Sharing discernment
discernment	-1.170***	0.716***	0.375**
	(0.145)	(0.169)	(0.179)
inoculation	0.980***	-0.246***	-0.0980
	(0.0961)	(0.0847)	(0.0852)
discernmentxinoculation	-0.984***	0.361***	0.177**
	(0.137)	(0.0967)	(0.0722)
Constant	4.293***	3.560***	2.414***
	(0.100)	(0.119)	(0.0948)
Observations	11,239	11,239	11,239
R-squared	0.190	0.072	0.016

Robust standard errors in parentheses
*** p<0.01, ** p<0.05, * p<0.1

Table S31.

Study 4 (scapegoating): linear regression for technique recognition, trustworthiness discernment and sharing discernment, with robust standard errors clustered on study participants and stimuli (manipulative vs neutral). Outcome variable of interest is "discernmentxinoculation".

Variables	Technique recognition	Trustworthiness discernment	Sharing discernment
discernment	-2.241***	1.150***	0.540***
	(0.192)	(0.118)	(0.0823)
inoculation	0.476***	-0.133	-0.0210
	(0.0932)	(0.0879)	(0.0871)
discernmentxinoculation	-0.479***	0.132	0.131*
	(0.130)	(0.0889)	(0.0762)
Constant	5.195***	2.989***	2.168***
	(0.109)	(0.0926)	(0.0759)
Observations	11,030	11,030	11,030
R-squared	0.348	0.123	0.028

Robust standard errors in parentheses
*** p<0.01, ** p<0.05, * p<0.1

Table S32.

Study 5 (ad hominem): linear regression for technique recognition, trustworthiness discernment and sharing discernment, with robust standard errors clustered on study participants and stimuli (manipulative vs neutral). Outcome variable of interest is "discernmentxinoculation".

Variables	Technique recognition	Trustworthiness discernment	Sharing discernment
discernment	-1.695***	1.016***	0.580***
	(0.213)	(0.151)	(0.117)
inoculation	0.251**	-0.146**	-0.131*
	(0.103)	(0.0718)	(0.0763)
discernmentxinoculation	-0.765***	0.240***	0.175***
	(0.140)	(0.0751)	(0.0673)
Constant	5.237***	2.836***	2.070***
	(0.0745)	(0.0841)	(0.0701)
Observations	11,133	11,133	11,133
R-squared	0.257	0.114	0.038

Robust standard errors in parentheses
*** p<0.01, ** p<0.05, * p<0.1

Table S33.

Studies 1-5: ANOVA table for technique recognition, with "study" as the between-groups variable, control participants only.

ANOVA	Sum of Sq.	df	Mean Sq.	F	*p*	η²
Overall model	817	4	204.17	84.8	< .001	
Study	817	4	204.17	84.8	< .001	0.109
Residuals	6710	2787	2.41			

Study		Study	M_{diff}	SE	df	t	p_{tukey}	Cohen's *d*	95%CI
Ad hominem	-	Emotional language	0.38	0.09	2787	4.1	< .001	0.25	0.13, 0.36
	-	False dichotomies	0.59	0.09	2787	6.41	< .001	0.38	0.26, 0.50
	-	Incoherence	1.04	0.09	2787	11.24	< .001	0.67	0.55, 0.79
	-	Scapegoating	-0.55	0.09	2787	-5.96	< .001	-0.36	-0.47, -0.24
Emotional language	-	False dichotomies	0.21	0.09	2787	2.27	0.154	0.14	0.02, 0.25
	-	Incoherence	0.66	0.09	2787	7.09	< .001	0.43	0.31, 0.54
	-	Scapegoating	-0.94	0.09	2787	-10.02	< .001	-0.60	-0.72, -0.48
False dichotomies	-	Incoherence	0.45	0.09	2787	4.85	< .001	0.29	0.17, 0.41
	-	Scapegoating	-1.15	0.09	2787	-12.37	< .001	-0.74	-0.86, -0.62
Incoherence	-	Scapegoating	-1.60	0.09	2787	-17.19	< .001	-1.03	-1.15, -0.91

Note. Comparisons are based on estimated marginal means

Table S34.

Studies 1-5: ANOVA table for trustworthiness discernment, with “study” as the between-groups variable, control participants only.

ANOVA	Sum of Sq.	df	Mean Sq	F	*p*	η^2
Overall model	73.3	4	18.33	13.8	< .001	
Study	73.3	4	18.33	13.8	< .001	0.019
Residuals	3711.6	2787	1.33			

Study		Study	M_{diff}	SE	df	t	p_{tukey}	Cohen's *d*	95%CI
Ad hominem	-	Emotional language	0.171	0.069	2787	2.46	0.099	0.15	0.03, 0.27
	-	False dichotomies	0.294	0.069	2787	4.28	< .001	0.26	0.14, 0.37
	-	Incoherence	-0.074	0.069	2787	-1.08	0.818	-0.06	-0.18, 0.05
	-	Scapegoating	-0.147	0.069	2787	-2.12	0.210	-0.13	-0.24, -0.01
Emotional language	-	False dichotomies	0.124	0.069	2787	1.79	0.381	0.11	-0.01, 0.22
	-	Incoherence	-0.245	0.069	2787	-3.54	0.004	-0.21	-0.33, -0.09
	-	Scapegoating	-0.318	0.070	2787	-4.57	< .001	-0.28	-0.39, -0.16
False dichotomies	-	Incoherence	-0.369	0.069	2787	-5.36	< .001	-0.32	-0.44, -0.20
	-	Scapegoating	-0.441	0.069	2787	-6.4	< .001	-0.38	-0.50, -0.26
Incoherence	-	Scapegoating	-0.073	0.069	2787	-1.05	0.831	-0.06	-0.18, 0.05

Note. Comparisons are based on estimated marginal means

Table S35.

Studies 1-5: Student's and Bayesian *t*-tests for technique recognition, confidence (manipulative and neutral posts), trustworthiness discernment, and sharing discernment, by study, with "never sharers" excluded.

Study	Variable	Type	Statistic	±%	df	*p*	M_{diff}	95%CI	Cohen's *d*	95%CI
1 Emotional language	Technique rec.	Student's t	7.04		931	< .001	0.66	0.48, 0.84	0.46	0.33, 0.59
		BF_{10}	1.60e0+9	5.82E-16						
	Confidence (manipulative)	Student's t	7.74		932	< .001	0.55	0.41, 0.69	0.51	0.37, 0.64
		BF_{10}	1.98E+11	3.55E-18						
	Confidence (neutral)	Student's t	3.65		932	< .001	0.26	0.12, 0.39	0.24	0.11, 0.37
		BF_{10}	49.8	5.58e0-8						
	Trustworthiness disc.	Student's t	3.67		931	< .001	0.29	0.13, 0.44	0.24	0.11, 0.37
		BF_{10}	52.7	5.11e0-8						
	Sharing disc.	Student's t	3.53		931	< .001	0.26	0.12, 0.41	0.23	0.10, 0.36
		BF_{10}	32.2	8.62e0-8						
2 Incoherence	Technique rec.	Student's t	8.56		872	< .001	0.87	0.67, 1.07	0.58	0.44, 0.72
		BF_{10}	9.17E+13	1.13E-20						
	Confidence (manipulative)	Student's t	1.27		874	0.204	0.09	-0.05, 0.24	0.09	-0.05, 0.22
		BF_{10}	0.167	2.05e0-6						
	Confidence (neutral)	Student's t	-1.42		872	0.157	-0.10	-0.24, 0.04	-0.10	-0.23, 0.04
		BF_{10}	0.203	1.39e0-6						
	Trustworthiness disc.	Student's t	2.9		872	0.004	0.25	0.08, 0.41	0.20	0.06, 0.33
		BF_{10}	4.653	3.09e0-8						
	Sharing disc.	Student's t	2.332		872	0.02	0.17	0.03, 0.31	0.16	0.02, 0.29
		BF_{10}	1.089	1.78e0-7						
3 False dichotomies	Technique disc.	Student's t	9.86		906	< .001	1.05	0.84, 1.25	0.66	0.52, 0.79
		BF_{10}	4.75E+18	1.07E-25						
	Confidence (manipulative)	Student's t	7.09		907	< .001	0.51	0.37, 0.65	0.47	0.34, 0.60
		BF_{10}	2.21e0+9	7.27E-17						
	Confidence (neutral)	Student's t	3.81		906	< .001	0.29	0.14, 0.44	0.25	0.12, 0.38
		BF_{10}	90.7	1.11e0-8						
	Trustworthiness disc.	Student's t	5.31		906	< .001	0.41	0.26, 0.57	0.35	0.22, 0.48
		BF_{10}	63990.9	8.11E-12						
	Sharing disc.	Student's t	3.95		906	< .001	0.29	0.15, 0.43	0.26	0.13, 0.39
		BF_{10}	152.8	6.27e0-9						
4 Scapegoating	Technique rec.	Student's t	3.98		850	< .001	0.47	0.24, 0.70	0.27	0.14, 0.41
		BF_{10}	172.734	3.29e0-9						
	Confidence (manipulative)	Student's t	5.09		849	< .001	0.38	0.23, 0.53	0.35	0.21, 0.49
		BF_{10}	21804.322	3.73E-11						
	Confidence (neutral)	Student's t	1.56		850	0.118	0.12	-0.03, 0.28	0.11	-0.03, 0.24
		BF_{10}	0.254	7.98e0-5						
	Trustworthiness disc.	Student's t	1.92		850	0.055	0.17	0.00, 0.35	0.13	0.00, 0.27
		BF_{10}	0.471	3.57e0-5						
	Sharing disc.	Student's t	1.84		850	0.067	0.15	-0.01, 0.30	0.13	-0.01, 0.26
		BF_{10}	0.4	1.22e0-5						
5 Ad hominem	Technique rec.	Student's t	6.05		882	< .001	0.70	0.47, 0.93	0.41	0.27, 0.54
		BF_{10}	3.64E+06	1.21E-11						
	Confidence (manipulative)	Student's t	4.36		882	< .001	0.34	0.19, 0.50	0.29	0.16, 0.43
		BF_{10}	783.52	1.86E-10						
	Confidence (neutral)	Student's t	5.74		882	< .001	0.45	0.30, 0.60	0.39	0.25, 0.52
		BF_{10}	632560.93	3.45E-13						
	Trustworthiness disc.	Student's t	3.51		882	< .001	0.28	0.12, 0.44	0.24	0.10, 0.37
		BF_{10}	31.15	8.26e0-9						
	Sharing disc.	Student's t	3.08		882	0.002	0.22	0.08, 0.36	0.21	0.07, 0.34
		BF_{10}	7.91	4.16e0-8						

[a] Levene's test is significant (p < .05), suggesting a violation of the assumption of equal variances

Table S36.

Studies 1-5: Linear regressions with "Fake-Confidence" (confidence in technique recognition for the manipulative/misinformation stimuli) as dependent variable and perceived use of a manipulation technique in manipulative stimuli (Fake-Manipulativeness, Fake-Incoherence, Fake-Dichotomy, Fake-Scapegoating and Fake-AdHominem) and condition (inoculation – control) as independent variables. Note that in all 5 studies, the perceived use of a technique in manipulative social media content is significantly and positively correlated with participants' confidence in recognizing these techniques, when controlling for the condition that participants were assigned to.

Model fit measures - Fake-Confidence

			Overall Model Test			
**Study**	**R**	**R^2**	**F**	**df1**	**df2**	***p***
1 Emotional language	0.543	0.295	224	2	1068	< .001
2 Incoherence	0.241	0.0579	33.3	2	1083	< .001
3 False dichotomies	0.464	0.215	150	2	1091	< .001
4 Scapegoating	0.695	0.483	502	2	1075	< .001
5 Ad hominem	0.65	0.423	395	2	1078	< .001

Model coefficients - Fake-Confidence

**Study**	**Predictor**	**b**	**SE**	**t**	***p***
1 Emotional language	Intercept [a]	3.097	0.1224	25.29	< .001
	Condition:				
	Inoculation – Control	0.228	0.0588	3.87	< .001
	Fake-Manipulativeness	0.45	0.0237	18.94	< .001
2 Incoherence	Intercept [a]	4.888	0.0995	49.11	< .001
	Condition:				
	Inoculation – Control	-0.238	0.0738	-3.23	0.001
	Fake-Incoherence	0.199	0.0245	8.12	< .001
3 False dichotomies	Intercept [a]	3.526	0.1119	31.5	< .001
	Condition:				
	Inoculation – Control	0.159	0.065	2.44	0.015
	Fake-Dichotomy	0.36	0.0241	14.96	< .001
4 Scapegoating	Intercept [a]	1.9492	0.1163	16.76	< .001
	Condition:				
	Inoculation – Control	0.0847	0.0498	1.7	0.089
	Fake-Scapegoating	0.6525	0.0213	30.67	< .001
5 Ad hominem	Intercept [a]	1.615	0.1371	11.78	< .001
	Condition:				
	Inoculation – Control	0.151	0.0586	2.57	0.01
	Fake-AdHominem	0.685	0.0248	27.62	< .001

[a] Represents reference level

Table S37.

Studies 1-5: Linear regressions with "Control-Confidence" (confidence in technique recognition for the non-manipulative/neutral stimuli) as dependent variable and perceived use of a manipulation technique in non-manipulative stimuli (Control-Manipulativeness, Control-Incoherence, Control-Dichotomy, Control-Scapegoating and Control-AdHominem) and condition (inoculation – control) as independent variables. Note that in all 5 studies, the perceived use of a technique in non-manipulative social media content is significantly and negatively correlated with participants' confidence in recognizing these techniques, when controlling for the condition that participants were assigned to.

Study	R	R²	F	df1	df2	*p*
1 Emotional language	0.131	0.0171	9.26	2	1068	< .001
2 Incoherence	0.14	0.0196	10.8	2	1081	< .001
3 False dichotomies	0.224	0.0503	28.9	2	1090	< .001
4 Scapegoating	0.256	0.0656	37.8	2	1076	< .001
5 Ad hominem	0.242	0.0584	33.4	2	1078	< .001

Model coefficients - Control-Confidence

Study	Predictor	b	SE	t	*p*
1 Emotional language	Intercept [a]	5.3354	0.1076	49.6	< .001
	Condition:				
	Inoculation – Control	0.2199	0.0651	3.37	< .001
	Control-Manipulativeness	-0.0735	0.0277	-2.65	0.008
2 Incoherence	Intercept [a]	5.9273	0.089	66.56	< .001
	Condition:				
	Inoculation – Control	-0.091	0.0661	-1.38	0.169
	Control-Incoherence	-0.1077	0.0265	-4.06	< .001
3 False dichotomies	Intercept [a]	5.599	0.1087	51.49	< .001
	Condition:				
	Inoculation – Control	0.264	0.0694	3.8	< .001
	Control-Dichotomy	-0.198	0.0309	-6.4	< .001
4 Scapegoating	Intercept [a]	5.868	0.0957	61.33	< .001
	Condition:				
	Inoculation – Control	0.125	0.068	1.84	0.066
	Control-Scapegoating	-0.242	0.0286	-8.47	< .001
5 Ad hominem	Intercept [a]	5.477	0.1202	45.56	< .001
	Condition:				
	Inoculation – Control	0.352	0.0759	4.64	< .001
	Control-AdHominem	-0.172	0.031	-5.56	< .001

[a] Represents reference level

Table S38.

Studies 1-5: ANOVAs for technique recognition (Diff-Technique) with the converted political ideology variable (left – moderate – right) and condition (inoculation – control) as independent variables. Significant interactions between political ideology and condition are marked in bold. See also Figure S2 and Table S39 for the Tukey post-hoc tests.

Study	ANOVA - Diff-Technique	Sum of Squares	df	Mean Square	F	*p*	η²p
1 Emotional language	Condition	86.3	1	86.32	44.16	< .001	0.04
	Political-Ideology-converted	64.6	2	32.29	16.52	< .001	0.03
	Condition ✻ Political-Ideology-converted	12	2	6	3.07	**0.047**	0.006
	Residuals	2079.8	1064	1.95			
2 Incoherence	Condition	187.02	1	187.02	86.82	< .001	0.075
	Political-Ideology-converted	80.06	2	40.03	18.58	< .001	0.033
	Condition ✻ Political-Ideology-converted	6.37	2	3.18	1.48	0.229	0.003
	Residuals	2322.14	1078	2.15			
3 False dichotomies	Condition	312.37	1	312.373	123.836	< .001	0.102
	Political-Ideology-converted	100.28	2	50.139	19.877	< .001	0.035
	Condition ✻ Political-Ideology-converted	1.92	2	0.96	0.381	0.683	0.001
	Residuals	2741.92	1087	2.522			
4 Scapegoating	Condition	50.06	1	50.06	16.963	< .001	0.016
	Political-Ideology-converted	28.39	2	14.19	4.81	0.008	0.009
	Condition ✻ Political-Ideology-converted	2.87	2	1.44	0.487	0.615	0.001
	Residuals	3166.34	1073	2.95			
5 Ad hominem	Condition	154	1	154.03	55.59	< .001	0.049
	Political-Ideology-converted	126.5	2	63.25	22.83	< .001	0.041
	Condition ✻ Political-Ideology-converted	10.7	2	5.34	1.93	0.146	0.004
	Residuals	2978.7	1075	2.77			

Table S39.

Tukey post-hoc tests for technique recognition (Diff-Technique) with the converted political ideology variable (left – moderate – right) and condition (inoculation – control) as independent variables. Relevant *p*-values for differences between inoculation and control conditions for the same political ideology are marked in bold. Note that the only non-significant difference between inoculation and control condition is for moderates for the “emotional language” study, and for moderates and right-wingers for the “scapegoating” study. See also Figure S2 and Table S38.

Post Hoc Comparisons - Condition ✻ Political-Ideology-converted

**Study**	**Condition**	**Pol.Ideol.-converted**		**Condition**	**Pol.Ideol.-converted**	**M_{diff}**	**SE**	**df**	**t**	**p_{tukey}**	**Cohen’s *d***
1 Emotional language	Control	Left	-	Control	Moderate	0.2989	0.154	1064	1.935	0.381	0.2138
			-	Control	Right	0.3548	0.145	1064	2.449	0.141	0.2538
			-	Inoculation	Left	-0.838	0.118	1064	-7.114	**< .001**	-0.5994
			-	Inoculation	Moderate	0.0169	0.163	1064	0.104	1	0.0121
			-	Inoculation	Right	-0.3759	0.139	1064	-2.71	0.074	-0.2689
		Moderate	-	Control	Right	0.0559	0.177	1064	0.315	1	0.0399
			-	Inoculation	Left	-1.1369	0.156	1064	-7.293	< .001	0.8132
			-	Inoculation	Moderate	-0.282	0.192	1064	-1.468	**0.685**	-0.2017
			-	Inoculation	Right	-0.6748	0.172	1064	-3.917	0.001	-0.4827
		Right	-	Inoculation	Left	-1.1928	0.146	1064	-8.149	< .001	0.8532
			-	Inoculation	Moderate	-0.3379	0.184	1064	-1.832	0.445	0.2417
			-	Inoculation	Right	-0.7307	0.164	1064	-4.464	**< .001**	-0.5226
	Inoculation	Left	-	Inoculation	Moderate	0.8549	0.164	1064	5.212	< .001	0.6115
			-	Inoculation	Right	0.4621	0.14	1064	3.293	0.013	0.3305
		Moderate	-	Inoculation	Right	-0.3928	0.18	1064	-2.187	0.245	-0.281
2 Incoherence	Control	Left	-	Control	Moderate	0.611	0.16	1078	3.83	0.002	0.416
			-	Control	Right	0.381	0.151	1078	2.52	0.12	0.26
			-	Inoculation	Left	-0.962	0.122	1078	-7.87	**< .001**	-0.656
			-	Inoculation	Moderate	-0.461	0.161	1078	-2.86	0.05	-0.314
			-	Inoculation	Right	-0.274	0.155	1078	-1.77	0.487	-0.186
		Moderate	-	Control	Right	-0.23	0.183	1078	-1.26	0.807	-0.157
			-	Inoculation	Left	-1.573	0.159	1078	-9.87	< .001	1.072
			-	Inoculation	Moderate	-1.072	0.191	1078	-5.61	**< .001**	-0.731
			-	Inoculation	Right	-0.884	0.185	1078	-4.77	< .001	-0.603
		Right	-	Inoculation	Left	-1.343	0.151	1078	-8.89	< .001	0.915
			-	Inoculation	Moderate	-0.842	0.184	1078	-4.57	< .001	0.574
			-	Inoculation	Right	-0.654	0.178	1078	-3.67	**0.003**	-0.446
	Inoculation	Left	-	Inoculation	Moderate	0.501	0.161	1078	3.11	0.024	0.341
			-	Inoculation	Right	0.689	0.155	1078	4.46	< .001	0.469
		Moderate	-	Inoculation	Right	0.188	0.187	1078	1	0.917	0.128
3 False dichotomies	Control	Left	-	Control	Moderate	0.377	0.182	1087	2.07	0.306	0.237
			-	Control	Right	0.818	0.161	1087	5.07	< .001	0.515
			-	Inoculation	Left	-1.068	0.133	1087	-8.06	**< .001**	-0.673
			-	Inoculation	Moderate	-0.767	0.167	1087	-4.59	< .001	-0.483
			-	Inoculation	Right	-0.451	0.159	1087	-2.84	0.052	-0.284
		Moderate	-	Control	Right	0.441	0.208	1087	2.12	0.277	0.278
			-	Inoculation	Left	-1.445	0.186	1087	-7.75	< .001	0.91
			-	Inoculation	Moderate	-1.143	0.212	1087	-5.38	**< .001**	-0.72
			-	Inoculation	Right	-0.828	0.206	1087	-4.02	< .001	-0.521
		Right	-	Inoculation	Left	-1.886	0.166	1087	-11.36	< .001	1.188
			-	Inoculation	Moderate	-1.584	0.195	1087	-8.14	< .001	0.998
			-	Inoculation	Right	-1.269	0.188	1087	-6.76	**< .001**	-0.799
	Inoculation	Left	-	Inoculation	Moderate	0.302	0.172	1087	1.76	0.493	0.19

			-	Inoculation	Right	0.617	0.163	1087	3.78	0.002	0.389
		Moderate	-	Inoculation	Right	0.316	0.192	1087	1.64	0.572	0.199
4 Scapegoating	Control	Left	-	Control	Moderate	0.26012	0.193	1073	1.3472	0.758	0.15143
			-	Control	Right	0.25669	0.175	1073	1.4683	0.685	0.14943
			-	Inoculation	Left	-0.52865	0.144	1073	-3.6766	**0.003**	-0.30774
			-	Inoculation	Moderate	-0.30332	0.192	1073	-1.5761	0.615	-0.17657
			-	Inoculation	Right	-0.05046	0.175	1073	-0.288	1	-0.02937
		Moderate	-	Control	Right	-0.00343	0.218	1073	-0.0158	1	-0.002
			-	Inoculation	Left	-0.78877	0.194	1073	-4.0712	< .001	0.45917
			-	Inoculation	Moderate	-0.56344	0.232	1073	-2.4269	**0.148**	-0.328
			-	Inoculation	Right	-0.31058	0.218	1073	-1.4241	0.712	-0.1808
		Right	-	Inoculation	Left	-0.78533	0.176	1073	-4.4737	< .001	0.45717
			-	Inoculation	Moderate	-0.56001	0.217	1073	-2.5783	0.103	0.326
			-	Inoculation	Right	-0.30715	0.202	1073	-1.5198	**0.652**	-0.1788
	Inoculation	Left	-	Inoculation	Moderate	0.22533	0.193	1073	1.1668	0.853	0.13117
			-	Inoculation	Right	0.47819	0.176	1073	2.7178	0.073	0.27837
		Moderate	-	Inoculation	Right	0.25286	0.218	1073	1.1624	0.855	0.1472
5 Ad hominem	Control	Left	-	Control	Moderate	0.9971	0.185	1075	5.397	< .001	0.599
			-	Control	Right	0.7418	0.172	1075	4.31	< .001	0.4456
			-	Inoculation	Left	-0.5593	0.137	1075	-4.089	**< .001**	-0.336
			-	Inoculation	Moderate	0.0392	0.179	1075	0.219	1	0.0236
			-	Inoculation	Right	-0.2208	0.188	1075	-1.172	0.85	-0.1327
		Moderate	-	Control	Right	-0.2553	0.21	1075	-1.216	0.829	-0.1534
			-	Inoculation	Left	-1.5564	0.182	1075	-8.544	< .001	0.935
			-	Inoculation	Moderate	-0.9579	0.216	1075	-4.443	**< .001**	-0.5754
			-	Inoculation	Right	-1.2179	0.224	1075	-5.449	< .001	-0.7317
		Right	-	Inoculation	Left	-1.3011	0.169	1075	-7.684	< .001	0.7816
			-	Inoculation	Moderate	-0.7026	0.205	1075	-3.43	0.008	0.4221
			-	Inoculation	Right	-0.9626	0.213	1075	-4.516	**< .001**	-0.5783
	Inoculation	Left	-	Inoculation	Moderate	0.5985	0.176	1075	3.397	0.009	0.3596
			-	Inoculation	Right	0.3385	0.186	1075	1.821	0.452	0.2033
		Moderate	-	Inoculation	Right	-0.2601	0.219	1075	-1.189	0.842	-0.1562

Note: Comparisons are based on estimated marginal means

Table S40.

Studies 1-5: ANOVAs for technique recognition (Diff-Technique) with the converted "bullshit receptivity" variable (high - low) and condition (inoculation – control) as independent variables. Significant interactions between "bullshit receptivity" and condition are marked in bold. See also Figure S3 and Table S41 for the Tukey post-hoc tests.

**Study**	**ANOVA - Diff-Technique**	**Sum of Squares**	**df**	**Mean Square**	**F**	***p***	**η²p**
1 Emotional language	Condition	127.58	1	127.58	66.866	< .001	0.059
	Bullshit-receptivity-converted	117.827	1	117.827	61.754	< .001	0.055
	Condition ✻ Bullshit-receptivity-converted	0.214	1	0.214	0.112	0.737	0.000
	Residuals	2035.83	1067	1.908			
2 Incoherence	Condition	216.6	1	216.57	100.8	< .001	0.085
	Bullshit-receptivity-converted	59.3	1	59.26	27.6	< .001	0.025
	Condition ✻ Bullshit-receptivity-converted	28.4	1	28.36	13.2	**< .001**	0.012
	Residuals	2321.4	1080	2.15			
3 False dichotomies	Condition	368.61	1	368.61	153.371	< .001	0.123
	Bullshit-receptivity-converted	226.59	1	226.59	94.28	< .001	0.08
	Condition ✻ Bullshit-receptivity-converted	1.25	1	1.25	0.521	0.471	0.000
	Residuals	2617.28	1089	2.4			
4 Scapegoating	Condition	52.1359	1	52.1359	18.5227	< .001	0.017
	Bullshit-receptivity-converted	171.6785	1	171.6785	60.9934	< .001	0.054
	Condition ✻ Bullshit-receptivity-converted	0.0358	1	0.0358	0.0127	0.91	0.000
	Residuals	3025.809	1075	2.8147			
5 Ad hominem	Condition	154	1	154.03	55.59	< .001	0.049
	Political-Ideology-converted	126.5	2	63.25	22.83	< .001	0.041
	Condition ✻ Political-Ideology-converted	10.7	2	5.34	1.93	0.146	0.004
	Residuals	2978.7	1075	2.77			
	Condition	155.89339	1	155.89339	59.11762	< .001	0.052
	Bullshit-receptivity-converted	275.11676	1	275.11676	104.3293	< .001	0.088
	Condition ✻ Bullshit-receptivity-converted	0.00514	1	0.00514	0.00195	0.965	0.000
	Residuals	2840.05313	1077	2.637			

Table S41.

Studies 1-5: Tukey post-hoc tests for technique recognition (Diff-Technique) with the converted "bullshit receptivity" variable (high - low) and condition (inoculation – control) as independent variables. Relevant *p*-values for differences between inoculation and control conditions for the same levels of "bullshit receptivity" are marked in bold. Note that technique recognition is significantly higher for the inoculation condition in all studies for both high- and low levels of "bullshit receptivity". See also Figure S3 and Table S40.

Post Hoc Comparisons - Condition ✻ Bullshit-receptivity-converted

Study	Condition	BS-Recep-converted		Condition	BS-Recep-converted	M_{diff}	SE	df	t	p_{tukey}	Cohen's *d*
1 Emotional language	Control	High	-	Control	Low	-0.6968	0.119	1067	-5.834	< .001	-0.5045
			-	Inoculation	High	-0.7239	0.113	1067	-6.419	**< .001**	-0.5241
			-	Inoculation	Low	-1.3637	0.121	1067	-11.295	< .001	-0.9873
		Low	-	Inoculation	High	-0.0271	0.12	1067	-0.226	0.996	0.0196
			-	Inoculation	Low	-0.6669	0.127	1067	-5.238	**< .001**	-0.4828
	Inoculation	High	-	Inoculation	Low	-0.6398	0.121	1067	-5.284	< .001	-0.4632
2 Incoherence	Control	High	-	Control	Low	-0.144	0.126	1080	-1.15	0.658	-0.0985
			-	Inoculation	High	-0.572	0.13	1080	-4.4	**< .001**	-0.3898
			-	Inoculation	Low	-1.364	0.126	1080	-10.85	< .001	-0.9304
		Low	-	Inoculation	High	-0.427	0.127	1080	-3.37	0.004	0.2913
			-	Inoculation	Low	-1.22	0.122	1080	-9.97	**< .001**	-0.8319
	Inoculation	High	-	Inoculation	Low	-0.793	0.127	1080	-6.25	< .001	-0.5406
3 False dichotomies	Control	High	-	Control	Low	-0.845	0.132	1089	-6.41	< .001	-0.545
			-	Inoculation	High	-1.096	0.132	1089	-8.28	**< .001**	-0.707
			-	Inoculation	Low	-2.077	0.137	1089	-15.12	< .001	-1.34
		Low	-	Inoculation	High	-0.251	0.128	1089	-1.96	0.205	0.162
			-	Inoculation	Low	-1.232	0.134	1089	-9.23	**< .001**	-0.795
	Inoculation	High	-	Inoculation	Low	-0.981	0.134	1089	-7.32	< .001	-0.633
4 Scapegoating	Control	High	-	Control	Low	-0.787	0.144	1075	-5.46	< .001	-0.469
			-	Inoculation	High	-0.429	0.144	1075	-2.98	**0.016**	-0.255
			-	Inoculation	Low	-1.239	0.141	1075	-8.76	< .001	-0.738
		Low	-	Inoculation	High	0.359	0.148	1075	2.43	0.073	-0.214
			-	Inoculation	Low	-0.452	0.145	1075	-3.11	**0.01**	-0.269
	Inoculation	High	-	Inoculation	Low	-0.81	0.145	1075	-5.59	< .001	-0.483
5 Ad hominem	Control	High	-	Control	Low	-1.012	0.141	1077	-7.16	< .001	-0.623
			-	Inoculation	High	-0.761	0.132	1077	-5.76	**< .001**	-0.468
			-	Inoculation	Low	-1.781	0.141	1077	-12.64	< .001	-1.097
		Low	-	Inoculation	High	0.251	0.141	1077	1.79	0.28	-0.155
			-	Inoculation	Low	-0.769	0.149	1077	-5.17	**< .001**	-0.474
	Inoculation	High	-	Inoculation	Low	-1.021	0.14	1077	-7.28	< .001	-0.629

Note. Comparisons are based on estimated marginal means

Table S42.

Studies 1-5: ANOVAs for technique recognition (Diff-Technique) with the converted analytical thinking variable (high - low) and condition (inoculation – control) as independent variables. See also Figure S4 and Table S42 for the Tukey post-hoc tests.

Study	ANOVA - Diff-Technique	Sum of Squares	df	Mean Square	F	*p*	η²p
1 Emotional language	Condition	128.99	1	128.99	66.16	< .001	0.058
	Analytic-Thinking-converted	70.56	1	70.56	36.19	< .001	0.033
	Condition ✻ Analytic-Thinking-converted	3.45	1	3.45	1.77	0.183	0.002
	Residuals	2080.25	1067	1.95			
2 Incoherence	Condition	230.3	1	230.3	105	< .001	0.089
	Analytic-Thinking-converted	35.53	1	35.53	16.2	< .001	0.015
	Condition ✻ Analytic-Thinking-converted	4.04	1	4.04	1.84	0.175	0.002
	Residuals	2368.77	1080	2.19			
3 False dichotomies	Condition	302.58	1	302.58	119.975	< .001	0.099
	Analytic-Thinking-converted	95.68	1	95.68	37.938	< .001	0.034
	Condition ✻ Analytic-Thinking-converted	2.28	1	2.28	0.903	0.342	0.001
	Residuals	2746.47	1089	2.52			
4 Scapegoating	Condition	61.193	1	61.193	20.865	< .001	0.019
	Analytic-Thinking-converted	44.137	1	44.137	15.049	< .001	0.014
	Condition ✻ Analytic-Thinking-converted	0.604	1	0.604	0.206	0.65	0.000
	Residuals	3152.824	1075	2.933			
5 Ad hominem	Condition	149.11	1	149.11	52.55	< .001	0.047
	Analytic-Thinking-converted	55.31	1	55.31	19.49	< .001	0.018
	Condition ✻ Analytic-Thinking-converted	4.09	1	4.09	1.44	0.23	0.001
	Residuals	3055.85	1077	2.84			

Table S43.

Studies 1-5: Tukey post-hoc tests for technique recognition (Diff-Technique) with the converted analytical thinking variable (high - low) and condition (inoculation – control) as independent variables. Relevant *p*-values for differences between inoculation and control conditions for the same levels of analytical thinking are marked in bold. Note that technique recognition is significantly higher for the inoculation condition in all studies for both high and low levels of analytical thinking. See also Figure S4 and Table S42.

Post Hoc Comparisons - Condition ✻ Analytic-Thinking-converted

Study	Condition	Analyt.Think.-converted		Condition	Analyt.Think.-converted	M_{diff}	SE	df	t	p_{tukey}	Cohen's *d*
1 Emotional language	Control	High		Control	Low	0.403	0.121	1067	3.33	0.005	0.289
			-	Inoculation	High	-0.814	0.129	1067	-6.31	**< .001**	-0.583
			-	Inoculation	Low	-0.182	0.123	1067	-1.49	0.447	-0.131
		Low	-	Inoculation	High	-1.217	0.121	1067	-10.09	< .001	0.872
			-	Inoculation	Low	-0.585	0.114	1067	-5.14	**< .001**	-0.419
	Inoculation	High	-	Inoculation	Low	0.632	0.122	1067	5.18	< .001	0.453
2 Incoherence	Control	High	-	Control	Low	0.242	0.128	1080	1.89	0.232	0.163
			-	Inoculation	High	-1.051	0.135	1080	-7.77	**< .001**	-0.71
			-	Inoculation	Low	-0.564	0.129	1080	-4.38	< .001	-0.381
		Low	-	Inoculation	High	-1.293	0.128	1080	-10.14	< .001	0.873
			-	Inoculation	Low	-0.805	0.12	1080	-6.69	**< .001**	-0.544
	Inoculation	High	-	Inoculation	Low	0.488	0.129	1080	3.79	< .001	0.329
3 False dichotomies	Control	High	-	Control	Low	0.686	0.136	1089	5.04	< .001	0.432
			-	Inoculation	High	-0.965	0.141	1089	-6.84	**< .001**	-0.608
			-	Inoculation	Low	-0.463	0.141	1089	-3.28	0.006	-0.291
		Low	-	Inoculation	High	-1.652	0.132	1089	-12.52	< .001	1.04
			-	Inoculation	Low	-1.149	0.132	1089	-8.72	**< .001**	-0.723
	Inoculation	High	-	Inoculation	Low	0.503	0.137	1089	3.67	0.001	0.317
4 Scapegoating	Control	High	-	Control	Low	0.3577	0.147	1075	2.43	0.072	0.2089
			-	Inoculation	High	-0.5244	0.152	1075	-3.461	**0.003**	-0.3062
			-	Inoculation	Low	-0.0719	0.148	1075	-0.487	0.962	-0.042
		Low	-	Inoculation	High	-0.882	0.148	1075	-5.974	< .001	0.515
			-	Inoculation	Low	-0.4296	0.144	1075	-2.989	**0.015**	-0.2508
	Inoculation	High	-	Inoculation	Low	0.4525	0.148	1075	3.054	0.012	0.2642
5 Ad hominem	Control	High	-	Control	Low	0.582	0.147	1077	3.97	< .001	0.346
			-	Inoculation	High	-0.627	0.157	1077	-3.98	**< .001**	-0.372
			-	Inoculation	Low	-0.294	0.145	1077	-2.03	0.179	-0.175
		Low	-	Inoculation	High	-1.21	0.148	1077	-8.16	< .001	0.718
			-	Inoculation	Low	-0.876	0.135	1077	-6.49	**< .001**	-0.52
	Inoculation	High	-	Inoculation	Low	0.333	0.147	1077	2.27	0.105	0.198

Note. Comparisons are based on estimated marginal means

Table S44.

Study 1 (emotional language): two-way interactions with technique discernment between condition and age, gender, education level, political ideology, populism, analytical thinking, bullshit receptivity, conspiracy belief, numerical thinking, social media use, and how often people check the news.

	**Age**			**Gender**			**Education**			**Political Ideology**			**Populism**			**Analytical Thinking**		
Predictors	*b*	*95% CI*	*p*	*b*	*CI*	*p*	*b*	*95% CI*	*p*	*b*	*95% CI*	*p*	*b*	*CI*	*p*	*b*	*95% CI*	*p*
(Intercept)	1.36	1.07 – 1.65	**<0.001**	1.25	1.07 – 1.43	**<0.001**	1.86	1.34 – 2.38	**<0.001**	1.71	1.46 – 1.96	**<0.001**	1.65	0.91 – 2.40	**<0.001**	1.11	0.94 – 1.28	**<0.001**
Condition	0.55	0.14 – 0.97	**0.009**	0.63	0.38 – 0.87	**<0.001**	0.45	-0.29 – 1.19	0.228	0.76	0.40 – 1.12	**<0.001**	1.59	0.52 – 2.65	**0.003**	0.55	0.30 – 0.79	**<0.001**
Age	0	-0.08 – 0.08	0.984															
Age*Condition	0.03	-0.09 – 0.15	0.611															
Gender				0.17	-0.08 – 0.41	0.183												
Gender*Condition				0.12	-0.23 – 0.46	0.505												
Education							-0.13	-0.26 – 0.00	0.057									
Education*Condition							0.05	-0.14 – 0.24	0.596									
Political-Ideology										-0.1	-0.17 – -0.04	**0.003**						
Political-Ideology*Condition										-.03	-0.12 – 0.07	0.593						
Populism													-0.08	-0.27 – 0.12	0.439			
Populism*Condition													-0.24	-0.52 – 0.03	0.087			
Analytical-Thinking																0.2	0.10 – 0.30	**<0.001**
Analytical-Thinking*Condition																0.08	-0.06 – 0.21	0.269
Observations	1096			1081			1096			1094			1094			1094		
R2*R2 adj.	0.050 / 0.047			0.059 / 0.057			0.053 / 0.051			0.070 / 0.067			0.060 / 0.057			0.092 / 0.089		

	**Bullshit Receptivity**			**Conspiracy Belief**			**Numeracy**			**Soc. Media. Use**			**News Checking**		
	b	*95% CI*	*p*	*b*	*95% CI*	*p*	*b*	*95% CI*	*p*	*b*	*95% CI*	*p*	*b*	*95% CI*	*p*
(Intercept)	2.5	2.15 – 2.86	**<0.001**	2	1.63 – 2.38	**<0.001**	0.5	0.17 – 0.84	**0.003**	1.35	0.87 – 1.82	**<0.001**	1.27	0.75 – 1.79	**<0.001**
Condition	0.67	0.17 – 1.17	**0.009**	0.78	0.25 – 1.30	**0.004**	0.57	0.08 – 1.06	**0.022**	1.29	0.60 – 1.97	**<0.001**	0.28	-0.47 – 1.02	0.466
Bullshit-Receptivity	-0.42	-0.54 – -0.30	**<0.001**												
Bullshit-Receptivity*Condition	0.01	-0.16 – 0.18	0.929												
Conspiracy-Belief				-1.03	-1.60 – -0.46	**<0.001**									
Conspiracy-Belief *Condition				-0.17	-0.97 – 0.62	0.671									

Numerical-Thinking							0.29	0.19 – 0.40	**<0.001**						
Numerical-Thinking*Condition							0.03	-0.13 – 0.19	0.713						
Social-Media-Use										0	-0.11 – 0.12	0.944			
Social-Media-Use*Condition										-0.16	-0.33 – 0.01	0.061			
News-Check													0.03	-0.11 – 0.16	0.704
News-Check*Condition													0.1	-0.09 – 0.29	0.32
Observations		1094			1094			1094			1095			1096	
R2*R2 adj.		0.124 / 0.121			0.076 / 0.074			0.100 / 0.098			0.055 / 0.053			0.052 / 0.049	

Table S45.

Study 2 (incoherence): two-way interactions with technique discernment between condition and age, gender, education level, political ideology, populism, analytical thinking, bullshit receptivity, conspiracy belief, numerical thinking, social media use, and how often people check the news.

	Age			Gender			Education			Political Ideology			Populism			Analytical Thinking		
Predictors	*b*	*95% CI*	*p*	*b*	*95% CI*	*p*	*b*	*95% CI*	*p*	*b*	*95% CI*	*p*	*b*	*95% CI*	*p*	*b*	*95% CI*	*p*
(Intercept)	1.18	0.85 – 1.50	**<0.001**	0.86	0.68 – 1.03	**<0.001**	0.52	-0.04 – 1.08	0.068	1.08	0.82 – 1.35	**<0.001**	0.87	0.10 – 1.63	**0.026**	0.6	0.41 – 0.79	**<0.001**
Condition	0.46	-0.00 – 0.92	0.052	0.77	0.52 – 1.02	**<0.001**	1.46	0.67 – 2.25	**<0.001**	1.11	0.74 – 1.49	**<0.001**	1.29	0.22 – 2.36	**0.018**	0.69	0.42 – 0.96	**<0.001**
Age	-0.13	-0.22 – -0.05	**0.003**															
Age * Condition	0.13	0.00 – 0.25	**0.043**															
Gender				-0.26	-0.51 – -0.01	**0.038**												
Gender * Condition				0.22	-0.13 – 0.57	0.223												
Education							0.05	-0.09 – 0.19	0.476									
Education * Condition							-0.15	-0.34 – 0.05	0.15									
Political-Ideology										-0.11	-0.18 – -0.04	**0.002**						
Political-Ideology * Condition										-0.07	-0.17 – 0.03	0.181						
Populism													-0.04	-0.24 – 0.16	0.701			
Populism * Condition													-0.11	-0.40 – 0.18	0.455			
Analytical-Thinking																0.1	-0.02 – 0.23	0.113
Analytical-Thinking * Condition																0.18	0.00 – 0.36	**0.047**
Observations	1108			1095			1108			1107			1106			1106		
R^2 / R^2 adjusted	0.091 / 0.088			0.084 / 0.082			0.085 / 0.083			0.109 / 0.107			0.085 / 0.082			0.101 / 0.098		

	Bullshit Receptivity			Conspiracy Belief			Numeracy			Soc. Media. Use			News Checking		
	b	*95% CI*	*p*	*b*	*95% CI*	*p*	*b*	*95% CI*	*p*	*b*	*95% CI*	*p*	*b*	*95% CI*	*p*
(Intercept)	0.86	0.50 – 1.23	**<0.001**	0.81	0.46 – 1.16	**<0.001**	0.25	-0.13 – 0.62	0.196	0.49	0.03 – 0.95	**0.036**	0.38	-0.17 – 0.94	0.176
Condition	2.05	1.54 – 2.56	**<0.001**	1.5	1.01 – 1.99	**<0.001**	-0.1	-0.63 – 0.43	0.719	1.6	0.93 – 2.27	**<0.001**	1.12	0.33 – 1.90	**0.005**
Bullshit-Receptivity	-0.06	-0.19 – 0.08	0.412												
Bullshit-Receptivity * Condition	-0.45	-0.63 – -0.26	**<0.001**												
Conspiracy-Belief				-0.16	-0.71 – 0.40	0.579									
Conspiracy-Belief * Condition				-1.02	-1.79 – -0.25	**0.01**									
Numerical-Thinking							0.16	0.04 – 0.28	**0.009**						
Numerical-Thinking * Condition							0.34	0.17 – 0.50	**<0.001**						
Social-Media-Use										0.06	-0.06 – 0.18	0.314			

Social-Media-Use * Condition										-0.18	-0.35 – -0.01	**0.034**			
News-Check													0.09	-0.05 – 0.23	0.228
News-Check * Condition													-0.06	-0.26 – 0.14	0.574
Observations		1106			1106			1106			1107			1107	
R^2 / R^2 adjusted		0.130 / 0.127			0.098 / 0.096			0.140 / 0.138			0.087 / 0.084			0.084 / 0.082	

Table S46.

Study 3 (false dichotomies): two-way interactions with technique discernment between condition and age, gender, education level, political ideology, populism, analytical thinking, bullshit receptivity, conspiracy belief, numerical thinking, social media use, and how often people check the news.

	Age			Gender			Education			Political Ideology			Populism			Analytical Thinking		
Predictors	*b*	*95% CI*	*p*	*b*	*95% CI*	*p*	*b*	*95% CI*	*p*	*b*	*95% CI*	*p*	*b*	*95% CI*	*p*	*b*	*95% CI*	*p*
(Intercept)	1.45	1.10 – 1.81	**<0.001**	1.17	0.97 – 1.36	**<0.001**	1.09	0.50 – 1.69	**<0.001**	1.91	1.63 – 2.20	**<0.001**	2.38	1.53 – 3.23	**<0.001**	0.73	0.53 – 0.94	**<0.001**
Condition	0.78	0.27 – 1.29	**0.003**	0.83	0.56 – 1.10	**<0.001**	1.28	0.41 – 2.15	**0.004**	0.86	0.45 – 1.26	**<0.001**	1.5	0.33 – 2.68	**0.012**	1.07	0.78 – 1.37	**<0.001**
Age	-0.09	-0.18 – 0.01	0.077															
Age * Condition	0.08	-0.06 – 0.21	0.273															
Gender				-0.07	-0.34 – 0.20	0.616												
Gender * Condition				0.47	0.09 – 0.85	**0.015**												
Education							0.02	-0.13 – 0.16	0.838									
Education * Condition							-0.06	-0.28 – 0.16	0.585									
Political-Ideology										-0.23	-0.31 – -0.15	**<0.001**						
Political-Ideology * Condition										0.07	-0.04 – 0.17	0.209						
Populism													-0.33	-0.56 – -0.10	**0.004**			
Populism * Condition													-0.12	-0.43 – 0.19	0.448			
Analytical-Thinking																0.37	0.23 – 0.50	**<0.001**
Analytical-Thinking * Condition																-0.05	-0.24 – 0.14	0.616
Observations		1117			1109			1117			1113			1112			1112	
R^2 / R^2 adjusted		0.096 / 0.093			0.104 / 0.102			0.093 / 0.091			0.137 / 0.134			0.114 / 0.112			0.132 / 0.130	

	Bullshit Receptivity			Conspiracy Belief			Numeracy			Soc. Media. Use			News Checking		
	b	*95% CI*	*p*	*b*	*95% CI*	*p*	*b*	*95% CI*	*p*	*b*	*95% CI*	*p*	*b*	*95% CI*	*p*
(Intercept)	2.5	2.13 – 2.88	**<0.001**	2.26	1.90 – 2.61	**<0.001**	-0.17	-0.61 – 0.26	0.433	1.28	0.76 – 1.80	**<0.001**	0.97	0.39 – 1.56	**0.001**
Condition	1.45	0.91 – 1.98	**<0.001**	1.05	0.54 – 1.55	**<0.001**	0.7	0.12 – 1.28	**0.018**	1.06	0.33 – 1.79	**0.004**	0.73	-0.09 – 1.56	0.082
Bullshit-Receptivity	-0.51	-0.65 – -0.38	**<0.001**												
Bullshit-Receptivity * Condition	-0.12	-0.31 – 0.06	0.197												
Conspiracy-Belief				-1.9	-2.47 – -1.33	**<0.001**									
Conspiracy-Belief * Condition				0.08	-0.72 – 0.88	0.853									
Numerical-Thinking							0.44	0.30 – 0.58	**<0.001**						
Numerical-Thinking * Condition							0.12	-0.06 – 0.30	0.19						
Social-Media-Use										-0.03	-0.17 – 0.10	0.632			

Social-Media-Use * Condition										0	-0.19 – 0.18	0.975			
News-Check													0.05	-0.10 – 0.20	0.533
News-Check * Condition													0.09	-0.13 – 0.30	0.431
Observations		1112			1112			1112			1113			1113	
R^2 / R^2 adjusted		0.199 / 0.197			0.157 / 0.155			0.185 / 0.183			0.095 / 0.093			0.097 / 0.095	

Table S47.

Study 4 (scapegoating): two-way interactions with technique discernment between condition and age, gender, education level, political ideology, populism, analytical thinking, bullshit receptivity, conspiracy belief, numerical thinking, social media use, and how often people check the news.

	**Age**			**Gender**			**Education**			**Political Ideology**			**Populism**			**Analytical Thinking**		
Predictors	*b*	*95% CI*	*p*	*b*	*95% CI*	*p*	*b*	*95% CI*	*p*	*b*	*95% CI*	*p*	*b*	*95% CI*	*p*	*b*	*95% CI*	*p*
(Intercept)	1.95	1.59 – 2.31	**<0.001**	2.15	1.95 – 2.36	**<0.001**	3.33	2.68 – 3.98	**<0.001**	2.57	2.27 – 2.87	**<0.001**	2.43	1.56 – 3.30	**<0.001**	2.11	1.88 – 2.33	**<0.001**
Condition	0.59	0.06 – 1.11	**0.028**	0.38	0.08 – 0.67	**0.013**	0.33	-0.57 – 1.23	0.473	0.61	0.18 – 1.05	**0.006**	0.54	-0.76 – 1.83	0.417	0.42	0.10 – 0.75	**0.01**
Age	0.11	0.01 – 0.22	**0.034**															
Age * Condition	-0.05	-0.20 – 0.10	0.494															
Gender				0.29	-0.00 – 0.58	0.052												
Gender * Condition				0.09	-0.33 – 0.50	0.68												
Education							-0.26	-0.42 – -0.10	**0.002**									
Education * Condition							0.03	-0.20 – 0.25	0.812									
Political-Ideology										-0.08	-0.16 – 0.00	0.059						
Political-Ideology * Condition										-0.06	-0.17 – 0.06	0.335						
Populism													-0.03	-0.26 – 0.20	0.786			
Populism * Condition													-0.03	-0.37 – 0.32	0.867			
Analytical-Thinking																0.17	0.03 – 0.32	**0.021**
Analytical-Thinking * Condition																0.01	-0.20 – 0.22	0.938
Observations	1097			1082			1097			1095			1093			1093		
R^2 / R^2 adjusted	0.020 / 0.017			0.024 / 0.021			0.032 / 0.029			0.027 / 0.024			0.015 / 0.012			0.025 / 0.022		

	**Bullshit Receptivity**			**Conspiracy Belief**			**Numeracy**			**Soc. Media. Use**			**News Checking**		
	b	*95% CI*	*p*	*b*	*95% CI*	*p*	*b*	*95% CI*	*p*	*b*	*95% CI*	*p*	*b*	*95% CI*	*p*
(Intercept)	3.56	3.13 – 4.00	**<0.001**	3.37	2.94 – 3.81	**<0.001**	1.46	1.01 – 1.90	**<0.001**	2.24	1.71 – 2.76	**<0.001**	2.02	1.36 – 2.67	**<0.001**
Condition	0.48	-0.13 – 1.09	0.12	0.19	-0.41 – 0.79	0.535	0.32	-0.32 – 0.96	0.331	0.68	-0.10 – 1.45	0.086	0.84	-0.14 – 1.82	0.094
Bullshit-Receptivity	-0.46	-0.61 – -0.31	**<0.001**												
Bullshit-Receptivity * Condition	-0.03	-0.24 – 0.18	0.773												
Conspiracy-Belief				-1.75	-2.42 – -1.08	**<0.001**									
Conspiracy-Belief * Condition				0.37	-0.57 – 1.31	0.437									
Numerical-Thinking							0.29	0.15 – 0.44	**<0.001**						
Numerical-Thinking * Condition							0.03	-0.18 – 0.23	0.805						
Social-Media-Use										0.02	-0.11 – 0.15	0.772			

Social-Media-Use * Condition				-0.07	-0.26 – 0.13	0.507			
News-Check							0.08	-0.09 – 0.24	0.36
News-Check * Condition							-0.11	-0.36 – 0.14	0.395
Observations	1093	1093	1093	1095			1095		
R^2 / R^2 adjusted	0.079 / 0.077	0.052 / 0.050	0.045 / 0.042	0.015 / 0.013			0.016 / 0.013		

Table S48.

Study 5 (ad hominem): two-way interactions with technique discernment between condition and age, gender, education level, political ideology, populism, analytical thinking, bullshit receptivity, conspiracy belief, numerical thinking, social media use, and how often people check the news.

	**Age**			**Gender**			**Education**			**Political Ideology**			**Populism**			**Analytical Thinking**		
Predictors	*b*	*95% CI*	*p*	*b*	*95% CI*	*p*	*b*	*95% CI*	*p*	*b*	*95% CI*	*p*	*b*	*95% CI*	*p*	*b*	*95% CI*	*p*
(Intercept)	2.07	1.72 – 2.42	**<0.001**	1.74	1.54 – 1.94	**<0.001**	2.16	1.52 – 2.80	**<0.001**	2.5	2.19 – 2.81	**<0.001**	2.7	1.75 – 3.66	**<0.001**	1.35	1.13 – 1.57	**<0.001**
Condition	-0.11	-0.23 – 0.00	0.055															
Age	0.71	0.21 – 1.20	**0.005**	0.63	0.34 – 0.92	**<0.001**	0.71	-0.20 – 1.61	0.127	0.52	0.09 – 0.95	**0.018**	0.45	-0.87 – 1.76	0.506	0.9	0.59 – 1.21	**<0.001**
Age * Condition	0.02	-0.14 – 0.18	0.769															
Gender				0.01	-0.28 – 0.30	0.941												
Gender * Condition				0.25	-0.16 – 0.66	0.237												
Education							-0.1	-0.26 – 0.05	0.201									
Education * Condition							0.01	-0.21 – 0.24	0.907									
Political-Ideology										-0.22	-0.30 – -0.14	**<0.001**						
Political-Ideology * Condition										0.06	-0.05 – 0.18	0.28						
Populism													-0.25	-0.49 – -0.00	**0.05**			
Populism * Condition													0.08	-0.26 – 0.42	0.654			
Analytical-Thinking																0.36	0.21 – 0.50	**<0.001**
Analytical-Thinking * Condition																-0.12	-0.33 – 0.09	0.267
Observations		1103			1086			1103			1102			1101			1102	
R^2 / R^2 adjusted		0.053 / 0.050			0.050 / 0.047			0.050 / 0.047			0.083 / 0.080			0.052 / 0.050			0.073 / 0.071	

	**Bullshit Receptivity**			**Conspiracy Belief**			**Numeracy**			**Soc. Media. Use**			**News Checking**		
	b	*95% CI*	*p*	*b*	*95% CI*	*p*	*b*	*95% CI*	*p*	*b*	*95% CI*	*p*	*b*	*95% CI*	*p*
(Intercept)	3.36	2.93 – 3.79	**<0.001**	3	2.55 – 3.46	**<0.001**	0.04	-0.40 – 0.48	0.864	1.51	0.92 – 2.11	**<0.001**	1.12	0.52 – 1.71	**<0.001**
Condition	0.76	0.15 – 1.38	**0.015**	0.28	-0.35 – 0.91	0.389	1.43	0.82 – 2.05	**<0.001**	0.79	-0.03 – 1.62	0.06	1.19	0.34 – 2.04	**0.006**
Bullshit-Receptivity	-0.58	-0.73 – -0.43	**<0.001**												
Bullshit-Receptivity * Condition	0	-0.20 – 0.21	0.966												
Conspiracy-Belief				-1.95	-2.64 – -1.27	**<0.001**									
Conspiracy-Belief * Condition				0.7	-0.25 – 1.66	0.148									
Numerical-Thinking							0.56	0.43 – 0.70	**<0.001**						
Numerical-Thinking * Condition							-0.22	-0.41 – -0.03	**0.024**						
Social-Media-Use										0.06	-0.09 – 0.21	0.416			

Social-Media-Use * Condition										-0.01	-0.21 – 0.20	0.941			
News-Check													0.17	0.02 – 0.33	**0.031**
News-Check * Condition													-0.11	-0.33 – 0.11	0.307
Observations		1101			1101			1102			1103			1103	
R^2 / R^2 adjusted		0.139 / 0.137			0.085 / 0.082			0.118 / 0.116			0.049 / 0.046			0.052 / 0.049	

Table S49.

Study 6 (emotional language replication study): Student's and Bayesian *t*-tests for technique recognition, trustworthiness, and sharing. *Note:* "Fake" denotes the averaged scores (per participant) for the manipulative stimuli; "Real" denotes the averaged scores per participant for the non-manipulative stimuli.

Measure		Statistic	±%	df	*p*	M_{diff}	Cohen's *d*	95% CI
Discernment-Manipulativeness	Student's t	-10.869		1066	< .001	-0.94	-0.67	[-0.79, -0.54]
	BF_{10}	8.26E+22	1.39E-28					
Discernment-Trustworthiness	Student's t	-7.213		1063	< .001	-0.49	-0.44	[-0.56, -0.32]
	BF_{10}	5.45e0+9	3.34E-15					
Discernment-Sharing	Student's t	-5.547		1063	< .001	-0.37	-0.34	[-0.46, -0.22]
	BF_{10}	219328.3771	9.90E-11					
Fake-Manipulativeness	Student's t	-12.359		1066	< .001	-0.93	-0.76	[-0.88, -0.63]
	BF_{10}	3.45E+29	2.56E-35					
Real-Manipulativeness	Student's t	0.274		1063	0.785	0.02	0.02	[-0.10, 0.14]
	BF_{10}	0.071	4.08e0-4					
Fake-Trustworthiness	Student's t	3.97		1066	< .001	0.20	0.24	[0.12, 0.36]
	BF_{10}	154.894	1.66e0-7					
Real-Trustworthiness	Student's t	-4.867		1063	< .001	-0.29	-0.30	[-0.42, -0.18]
	BF_{10}	7246.488	3.19e0-9					
Fake-Sharing	Student's t	1.248		1066	0.212	0.10	0.08	[-0.04, 0.20]
	BF_{10}	0.148	2.00e0-4					
Real-Sharing	Student's t	-3.275		1063	0.001	-0.28	-0.20	[-0.32 -0.08]
	BF_{10}	13.276	1.97e0-6					

[a] Levene's test is significant ($p < .05$), suggesting a violation of the assumption of equal variances

Table S50.

Study 6 (emotional language replication study): ANOVAs for manipulativeness (technique recognition), trustworthiness, and sharing discernment as predicted by condition (control – inoculation) and outcome measure response order (manipulativeness – trustworthiness – sharing; trustworthiness – manipulativeness – sharing; or sharing – trustworthiness – manipulativeness).

ANOVA - Discernment-Manipulativeness

	**Sum of Squares**	**df**	**Mean Square**	**F**	***p***
Condition	237.713	1	237.7127	117.7046	< .001
Order	17.89	2	8.945	4.4292	0.012
Condition ✻ Order	0.191	2	0.0956	0.0473	0.954
Residuals	2156.901	1068	2.0196		

ANOVA - Discernment-Trustworthiness

	**Sum of Squares**	**df**	**Mean Square**	**F**	***p***
Condition	61.35	1	61.35	50.46	< .001
Order	5.94	2	2.97	2.44	0.088
Condition ✻ Order	2.55	2	1.27	1.05	0.351
Residuals	1294.68	1065	1.22		

ANOVA - Discernment-Sharing

	**Sum of Squares**	**df**	**Mean Square**	**F**	***p***
Condition	34.883	1	34.883	29.27	< .001
Order	5.742	2	2.871	2.409	0.09
Condition ✻ Order	0.968	2	0.484	0.406	0.666
Residuals	1269.227	1065	1.192		

Table S51.

Study 6 (emotional language replication study): Student's and Bayesian *t*-tests for technique recognition, trustworthiness, and sharing, separated by outcome measure response order. *Note:* "Fake" denotes the averaged scores (per participant) for the manipulative stimuli; "Real" for the non-manipulative stimuli. MTS = manipulativeness–trustworthiness–sharing; TSM = trustworthiness–sharing–manipulativeness; SMT = sharing–manipulativeness–trustworthiness.

**MTS**		**Statistic**	**±%**	**df**	***p***	**M_{diff}**	**Cohen's *d***	**95% CI**
Discernment-Manipulativeness	Student's t	-6.312		365	< .001	-0.96	-0.66	[-0.88, -0.44]
	BF_{10}	1.09E+07	5.90E-15					
Discernment-Trustworthiness	Student's t	-3.137		365	0.002	-0.37	-0.33	[-0.54, -0.12]
	BF_{10}	12.571	1.12e0-8					
Discernment-Sharing	Student's t	-3.023		365	0.003	-0.34	-0.32	[-0.52, -0.11]
	BF_{10}	9.032	1.57e0-8					
Fake-Manipulativeness	Student's t	-6.597		365	< .001	-0.81	-0.69	[-0.91, -0.47]
	BF_{10}	5.49E+07	1.04E-15					
Real-Manipulativeness	Student's t	1.282		365	0.201	0.15	0.13	[-0.07, 0.34]
	BF_{10}	0.255	5.72e0-7					
Fake-Trustworthiness	Student's t	0.77		365	0.442	0.07	0.08	[-0.12, 0.29]
	BF_{10}	0.154	9.39e0-7					
Real-Trustworthiness	Student's t	-3.003		365	0.003	-0.30	-0.31	[-0.52, -0.11]
	BF_{10}	8.522	1.67e0-8					
Fake-Sharing	Student's t	0.733		365	0.464	0.09	0.08	[-0.13, 0.28]
	BF_{10}	0.15	9.64e0-7					
Real-Sharing	Student's t	-1.721		365	0.086	-0.25	-0.18	[-0.39, 0.03]
	BF_{10}	0.479	3.06e0-7					
**TSM**		**Statistic**	**±%**	**df**	***p***	**M_{diff}**	**Cohen's *d***	**95% CI**
Discernment-Manipulativeness	Student's t	-6.151		362	< .001	-0.91	-0.64	[-0.86, -0.43]
	BF_{10}	4.46E+06	1.44E-14					
Discernment-Trustworthiness	Student's t	-5.369		361	< .001	-0.60	-0.56	[-0.78, -0.35]
	BF_{10}	79771.445	1.04E-12					
Discernment-Sharing	Student's t	-3.762		361	< .001	-0.44	-0.40	[-0.60, -0.18]
	BF_{10}	95.103	1.31e0-9					
Fake-Manipulativeness	Student's t	-6.638		362	< .001	-0.87	-0.70	[-0.91, -0.48]
	BF_{10}	6.86E+07	7.51E-16					
Real-Manipulativeness	Student's t	0.421		361	0.674	0.05	0.04	[-0.16, 0.25]
	BF_{10}	0.126	1.12e0-6					
Fake-Trustworthiness	Student's t	2.895		362	0.004	0.27	0.30	[0.10, 0.51]
	BF_{10}	6.318	2.23e0-8					
Real-Trustworthiness	Student's t	-3.403		361	< .001	-0.34	-0.36	[-0.57, -0.15]
	BF_{10}	28.485	4.61e0-9					
Fake-Sharing	Student's t	1.398		362	0.163	0.18	0.15	[-0.06, 0.35]
	BF_{10}	0.296	4.88e0-7					
Real-Sharing	Student's t	-1.827		361	0.069	-0.27	-0.19	[-0.40, 0.02]
	BF_{10}	0.575	2.50e0-7					
**SMT**		**Statistic**	**±%**	**df**	***p***	**M_{diff}**	**Cohen's *d***	**95% CI**
Discernment-Manipulativeness	Student's t	-6.337		341	< .001	-0.96	-0.69	[0.91, -0.46]
	BF_{10}	1.18e0+7	8.06E-16					
Discernment-Trustworthiness	Student's t	-3.858		339	< .001	-0.47	-0.42	[-0.64, -0.20]
	BF_{10}	134.884	3.45E-10					
Discernment-Sharing	Student's t	-2.581		339	0.01	-0.30	-0.28	[-0.49, -0.06]
	BF_{10}	2.864	2.66e0-8					
Fake-Manipulativeness	Student's t	-8.518		341	< .001	-1.15	-0.92	[-1.15, -0.69]
	BF_{10}	9.72E+12	3.83E-21					
Real-Manipulativeness	Student's t	-1.665		339	0.097	-0.19	-0.18	[-0.39, 0.03]
	BF_{10}	0.451	1.98e0-7					
Fake-Trustworthiness	Student's t	3.139		341	0.002	0.27	0.34	[0.12, 0.55]
	BF_{10}	12.862	5.53e0-9					
Real-Trustworthiness	Student's t	-1.923		339	0.055	-0.20	-0.21	[-0.42, 0.01]
	BF_{10}	0.701	1.23e0-7					
Fake-Sharing	Student's t	0.13		341	0.897	0.02	0.01	[-0.20, 0.23]
	BF_{10}	0.12	8.42e0-7					
Real-Sharing	Student's t	-1.941		339	0.053	-0.29	-0.21	[-0.42, 0.00]
	BF_{10}	0.726	1.19e0-7					

[a] Levene's test is significant ($p < .05$), suggesting a violation of the assumption of equal variances

Table S52.

Study 6 (emotional language replication study): linear regression for manipulativeness, trustworthiness and sharing discernment, as predicted by condition (control – inoculation) and the five personality dimensions from the 10-item Personality Inventory.

	Manipulativeness				Trustworthiness				Sharing			
**Predictor**	*b*	*SE*	*t*	*p*	*b*	*SE*	*t*	*p*	*b*	*SE*	*t*	*p*
Intercept [a]	1.1361	0.2784	4.0810	< .001	1.3744	0.2187	6.2850	< .001	0.0156	0.2170	0.0717	0.9430
Condition:												
Inoculation – Control	0.9351	0.0860	10.8730	< .001	0.4828	0.0675	7.1480	< .001	0.3639	0.0670	5.4279	< .001
Openness	0.0907	0.0376	2.4090	0.0160	0.0286	0.0295	0.9690	0.3330	0.0301	0.0293	1.0269	0.3050
Conscientiousness	0.0308	0.0399	0.7720	0.4400	0.0079	0.0314	0.2520	0.8010	-0.0012	0.0311	-0.0372	0.9700
Extraversion	-0.0960	0.0273	-3.5200	< .001	-0.0462	0.0214	-2.1610	0.0310	-0.0310	0.0212	-1.4608	0.1440
Agreeableness	0.0440	0.0385	1.1430	0.2530	0.0623	0.0302	2.0630	0.0390	0.0327	0.0299	1.0920	0.2750
Neuroticism	-0.0646	0.0313	-2.0610	0.0400	-0.0400	0.0246	-1.6250	0.1040	0.0158	0.0244	0.6463	0.5180
[a] Represents reference level												
		**R**	**R^2**			**R**	**R^2**			**R**	**R^2**	
		0.345	0.119			0.239	0.0573			0.181	0.0328	

Table S53.

Study 6 (emotional language replication study): linear regression for manipulativeness, trustworthiness and sharing discernment, as predicted by condition (control – inoculation) and actively open-minded thinking (AOT). See also Figure S5.

	Manipulativeness				Trustworthiness				Sharing			
**Predictor**	*b*	*SE*	*t*	*p*	*b*	*SE*	*t*	*p*	*b*	*SE*	*t*	*p*
Intercept [a]	-1.625	0.59	-2.76	0.006	0.194	0.488	0.398	0.691	-0.962	0.475	-2.03	0.043
Condition:												
Inoculation – Control	0.971	0.146	6.67	< .001	0.467	0.12	3.875	< .001	0.305	0.117	2.6	0.01
AOT	0.686	0.138	4.96	< .001	0.296	0.114	2.591	0.01	0.287	0.111	2.58	0.01
[a] Represents reference level												
		**R**	**R^2**			**R**	**R^2**			**R**	**R^2**	
		0.415	0.173			0.248	0.0617			0.198	0.0391	

Table S54.

Study 6 (emotional language replication study): linear regression for manipulativeness, trustworthiness and sharing discernment, as predicted by condition (control – inoculation) and Veracity Discernment Ability (MIST20_VDA, i.e., ability to distinguish true from false headlines), as measured by the 20-item Misinformation Susceptibility Test (MIST). See also Figure S6.

	Manipulativeness				Trustworthiness				Sharing			
**Predictor**	*b*	*SE*	*t*	*p*	*b*	*SE*	*t*	*p*	*b*	*SE*	*t*	*p*
Intercept [a]	-1.993	0.412	-4.83	< .001	-0.303	0.35	-0.865	0.388	-1.257	0.343	-3.66	< .001
Condition:												
Inoculation – Control	0.969	0.139	6.99	< .001	0.46	0.118	3.903	< .001	0.306	0.116	2.65	0.009
MIST20_VDA	3.883	0.479	8.1	< .001	2.085	0.407	5.123	< .001	1.794	0.399	4.5	< .001
[a] Represents reference level												
		**R**	**R^2**			**R**	**R^2**			**R**	**R^2**	
		0.508	0.258			0.335	0.112			0.277	0.0766	

Table S55.

Study 7 (YouTube field study): results from the field study for each item, showing the total number of YouTube users who saw each item, total samples sizes (number of survey responses) in the experimental (inoculation) and control conditions, number of correct responses in the experimental and control conditions, the proportion of correct responses in the experimental and control conditions, the results of two-sample *z*-tests, and the Cohen's *h* effect sizes.

Study	Total item views	Total *N*	$n_{control}$	$n_{inoculation}$	$n_{correct,control}$	$n_{correct,inoculation}$	$SD_{control}$	$SD_{inoculation}$	% correct (inoculation)	% correct (control)	*z*	*p*	Cohen's *h*
**Emotional language**													
Item 1 (airline)	645,000	4143	2083	2060	1390	1384	21.51	21.31	0.672	0.667	0.31	0.757	0.01
Item 2 (senior)	699,000	2393	1204	1189	644	691	17.31	17.01	0.581	0.535	2.28	**0.023**	0.09
Item 3 (TV show)	389,000	3725	1862	1863	669	741	20.70	21.13	0.398	0.359	2.42	**0.016**	0.08
Average (all 3 items)	1,733,000	10261	5149	5112	2703	2816	35.83	35.56	0.551	0.525	2.63	**0.009**	0.05
**False Dichotomies**													
Item 1 (education)	231,000	4186	2014	2172	669	940	21.13	23.09	0.433	0.332	6.69	**< 0.001**	0.21
Item 2 (caffeine)	378,000	4064	2035	2029	852	906	22.26	22.40	0.447	0.419	1.79	0.073	0.06
Item 3 (solution)	330,000	4121	2002	2119	757	950	21.70	22.89	0.448	0.378	4.57	**< 0.001**	0.14
Average (all 3 items)	939,000	12371	6051	6320	2278	2796	37.69	39.48	0.442	0.376	7.45	**< 0.001**	0.13
**All items**													
Average (all 6 items)	2,672,000	22632	11200	11432	4981	5612	52.59	53.45	0.491	0.445	6.96	**< 0.001**	0.09

Table S56.

Study 7 (YouTube field study): YouTube ad campaign budget, costs, view rates, median time between viewing the ad and responding to the survey question (solicitation gap), and costs per view, by campaign (emotional language video & false dichotomies video).

Campaign	Item	Budget	Cost	Impressions	Views	View rate	Median solicitation gap (hours)	Avg cost per view
Emotional language	1 (airline)	$ 10,000.00	$ 6,003.30	995219	140651	14.13%	17.8	$ 0.04
	2 (senior)	$ 11,000.00	$ 8,732.82	1275319	163892	12.85%	20.7	$ 0.05
	3 (TV show)	$ 10,000.00	$ 6,865.83	1019829	117828	11.55%	18.7	$ 0.06
	**Total**	**$ 31,000.00**	**$ 21,601.95**	**3290367**	**422371**	**12.84%**	**19.1**	**$ 0.05**
False dichotomies	1 (education)	$ 10,000.00	$ 5,112.68	540337	142230	26.32%	15.4	$ 0.04
	2 (caffeine)	$ 11,000.00	$ 8,799.83	868632	219869	25.31%	20.1	$ 0.04
	3 (solution)	$ 10,000.00	$ 6,784.74	747698	182877	24.46%	17.6	$ 0.04
	**Total**	**$ 31,000.00**	**$ 20,697.25**	**2156667**	**544976**	**25.36%**	**17.7**	**$ 0.04**
**Total**		**$ 62,000.00**	**$ 42,299.20**	**5447034**	**967347**	**19.10%**	**18.4**	**$ 0.05**

REFERENCES AND NOTES

1. S. Lewandowsky, U. K. H. Ecker, J. Cook, Beyond misinformation: Understanding and coping with the "Post-Truth" era. *J. Appl. Res. Mem. Cogn.* **6**, 353–369 (2017).

2. J. Roozenbeek, C. R. Schneider, S. Dryhurst, J. Kerr, A. L. J. Freeman, G. Recchia, A. M. van der Bles, S. van der Linden, Susceptibility to misinformation about COVID-19 around the world. *R. Soc. Open Sci.* **7**, 201199 (2020).

3. S. Loomba, A. de Figueiredo, S. J. Piatek, K. de Graaf, H. J. Larson, Measuring the impact of COVID-19 vaccine misinformation on vaccination intent in the UK and USA. *Nat. Hum. Behav.* **5**, 337–348 (2021).

4. J. Roozenbeek, S. van der Linden, Fake news game confers psychological resistance against online misinformation. *Humanit. Soc. Sci. Commun.* **5**, 1–10 (2019).

5. S. Lewandowsky, M. Yesilada, Inoculating against the spread of Islamophobic and radical-Islamist disinformation. *Cogn. Res. Princ. Implic.* **6**, 57 (2021).

6. G. Pennycook, Z. Epstein, M. Mosleh, A. Arechar, D. Eckles, D. G. Rand, Shifting attention to accuracy can reduce misinformation online. *Nature* **592**, 590–595 (2021).

7. M. Mosleh, C. Martel, D. Eckles, D. G. Rand, in *CHI '21: Proceedings of the 2021 CHI Conference on Human Factors in Computing Systems* (Yokohama, Japan, 8 to 13 May 2021), pp. 1–13.

8. J. Roozenbeek, S. van der Linden, How to combat health misinformation: A psychological approach. *Am. J. Health Promot.* **36**, 569–575 (2022).

9. S. Coleman, The elusiveness of political truth: From the conceit of objectivity to intersubjective judgement. *Eur. J. Commun.* **33**, 157–171 (2018).

10. F. Zollo, A. Bessi, M. Del Vicario, A. Scala, G. Caldarelli, L. Shekhtman, S. Havlin, W. Quattrociocchi, Debunking in a world of tribes. *PLOS ONE* **12**, 1–27 (2017).

11. B. Lyons, V. Mérola, J. Reifler, F. Stoeckel, How politics shape views toward fact-checking: Evidence from six european countries. *Int. J. Press.* **25**, 469–492 (2020).

12. J. J. Van Bavel, K. Baicker, P. S. Boggio, V. Capraro, A. Cichocka, M. Cikara, M. J. Crockett, A. J. Crum, K. M. Douglas, J. N. Druckman, J. Drury, O. Dube, N. Ellemers, E. J. Finkel, J. H. Fowler, M. Gelfand, S. Han, S. A. Haslam, J. Jetten, S. Kitayama, D. Mobbs, L. E. Napper, D. J. Packer, G. Pennycook, E. Peters, R. E. Petty, D. G. Rand, S. D. Reicher, S. Schnall, A. Shariff, L. J. Skitka, S. S. Smith, C. R. Sunstein, N. Tabri, J. A. Tucker, S. van der Linden, P. van Lange, K. A. Weeden, M. J. A. Wohl, J. Zaki, S. R. Zion, R. Willer, Using social and behavioural science to support COVID-19 pandemic response. *Nat. Hum. Behav.* **4**, 460–471 (2020).

13. S. Lewandowsky, U. K. H. Ecker, C. M. Seifert, N. Schwarz, J. Cook, Misinformation and its correction. *Psychol. Sci. Public Interes.* **13**, 106–131 (2012).

14. W. J. McGuire, D. Papageorgis, The relative efficacy of various types of prior belief-defense in producing immunity against persuasion. *J. Abnorm. Soc. Psychol.* **62**, 327–337 (1961).

15. J. Compton, in *The SAGE Handbook of Persuasion: Developments in Theory and Practice*, J. P. Dillard, L. Shen, Eds. (SAGE Publications Inc., ed. 2, 2013), pp. 220–236.

16. C. S. Traberg, J. Roozenbeek, S. van der Linden, Psychological inoculation against misinformation: Current evidence and future directions. *Ann. Am. Acad. Pol. Soc. Sci.* **700**, 136–151 (2022).

17. J. Cook, S. Lewandowsky, U. K. H. Ecker, Neutralizing misinformation through inoculation: Exposing misleading argumentation techniques reduces their influence. *PLOS ONE* **12**, 1–21 (2017).

18. C. Carrasco-Farré, The fingerprints of misinformation: How deceptive content differs from reliable sources in terms of cognitive effort and appeal to emotions. *Humanit. Soc. Sci. Commun.* **9**, 162 (2022).

19. A. Simchon, W. J. Brady, J. J. Van Bavel, Troll and divide: The language of online polarization. *PNAS Nexus* **1**, pgac019 (2021).

20. E. K. Vraga, S. C. Kim, J. Cook, Testing logic-based and humor-based corrections for science, health, and political misinformation on social media. *J. Broadcast. Electron. Media* **63**, 393–414 (2019).

21. H. O.-Y. Li, A. Bailey, D. Huynh, J. Chan, YouTube as a source of information on COVID-19: A pandemic of misinformation? *BMJ Glob. Heal.* **5**, e002604 (2020).

22. W. J. Brady, J. A. Wills, J. T. Jost, J. A. Tucker, J. J. Van Bavel, Emotion shapes the diffusion of moralized content in social networks. *Proc. Natl. Acad. Sci. U.S.A.* **114**, 7313–7318 (2017).

23. S. Lewandowsky, J. Cook, E. A. Lloyd, The "Alice in Wonderland" mechanics of the rejection of (climate) science: Simulating coherence by conspiracism. *Synthese* **195**, 175–196 (2016).

24. K. Escandón, A. L. Rasmussen, I. Bogoch, E. J. Murray, K. Escandón, J. Kindrachuk, COVID-19 and false dichotomies—A nuanced review of the evidence regarding public health, COVID-19 symptomatology, SARS-CoV-2 transmission, masks, and reinfection. *BMC Infect. Dis.* **21**, 710 (2021).

25. L. Atlani-Duault, J. K. Ward, M. Roy, C. Morin, A. Wilson, Tracking online heroisation and blame in epidemics. *Lancet Public Heal.* **5**, E137–E138 (2020).

26. D. Walton, *Ad Hominem Arguments* (The University of Alabama Press, 1998).

27. M. Basol, J. Roozenbeek, S. van der Linden, Good news about Bad News: Gamified inoculation boosts confidence and cognitive immunity against fake news. *J. Cogn.* **3**, 1–9 (2020).

28. S. McGrew, Learning to evaluate: An intervention in civic online reasoning. *Comput. Educ.* **145**, 103711 (2020).

29. G. Pennycook, J. Binnendyk, C. Newton, D. G. Rand, A practical guide to doing behavioral research on fake news and misinformation. *Collabra Psychol.* **7**, 25293 (2021).

30. Z. Epstein, N. Sirlin, A. Arechar, G. Pennycook, D. G. Rand, Social media sharing reduces truth discernment. *PsyArxiv Prepr.* 10.31234/osf.io/q4bd2 (2021).

31. J. J. Van Bavel, A. Pereira, The partisan brain: An identity-based model of political belief. *Trends Cogn. Sci.* **22**, 213–224 (2018).

32. G. Pennycook, D. G. Rand, Lazy, not biased: Susceptibility to partisan fake news is better explained by lack of reasoning than by motivated reasoning. *Cognition* **188**, 39–50 (2019).

33. J. Roozenbeek, R. Maertens, S. Herzog, M. Geers, R. Kurvers, M. Sultan, S. van der Linden, Susceptibility to misinformation is consistent across question framings and response modes and better explained by myside bias and partisanship than analytical thinking. *Judgm. Decis. Mak.* **17**, 547–573 (2022).

34. S. Rathje, J. Roozenbeek, C. S. Traberg, J. J. Van Bavel, S. van der Linden, Letter to the editors of psychological science: Meta-analysis reveals that accuracy nudges have little to no effect for US conservatives: Regarding pennycook et. al (2020). *Psychol. Sci.* 10.25384/SAGE.12594110.v2 (2022).

35. A. Akkerman, C. Mudde, A. Zaslove, How populist are the people? measuring populist attitudes in voters. *Comp. Polit. Stud.* **47**, 1324–1353 (2013).

36. G. Pennycook, J. A. Cheyne, N. Barr, D. J. Koehler, J. A. Fugelsang, On the reception and detection of pseudo-profound bullshit. *Judgm. Decis. Mak.* **10**, 549–563 (2015).

37. M. Bruder, P. Haffke, N. Neave, N. Nouripanah, R. Imhoff, Measuring individual differences in generic beliefs in conspiracy theories across cultures: Conspiracy mentality questionnaire. *Front. Psychol.* **4**, 225 (2013).

38. S. Frederick, Cognitive reflection and decision making. *J. Econ. Perspect.* **19**, 25–42 (2005).

39. S. D. Gosling, P. J. Rentfrow, W. B. Swann Jr., A very brief measure of the big-five personality domains. *J. Res. Pers.* **37**, 504–528 (2003).

40. J. Baron, Actively open-minded thinking in politics. *Cognition* **188**, 8–18 (2019).

41. R. Maertens, F. M. Götz, C. Schneider, J. Roozenbeek, J. Kerr, S. Stieger, W. P. McClanahan III, K. Drabot, S. van der Linden, The misinformation susceptibility test (MIST): A psychometrically validated measure of news veracity discernment. *PsyArxiv Prepr.* 10.31234/osf.io/gk68h (2022).

42. Wikipedia, List of most visited websites. *en.wikipedia.org* (2022); https://en.wikipedia.org/wiki/List_of_most_visited_websites.

43. M. Alfano, A. E. Fard, J. A. Carter, P. Clutton, C. Klein, Technologically scaffolded atypical cognition: The case of YouTube's recommender system. *Synthese* **199**, 835–858 (2021).

44. M. Faddoul, G. Chaslot, H. Farid, A longitudinal analysis of YouTube's promotion of conspiracy videos (6 March 2020); arXiv:2003.03318 [cs.CY].

45. A. M. Guess, M. Lerner, B. Lyons, J. M. Montgomery, B. Nyhan, J. Reifler, N. Sircar, A digital media literacy intervention increases discernment between mainstream and false news in the United States and India. *Proc. Natl. Acad. Sci. U.S.A.* **117**, 15536–15545 (2020).

46. R. Maertens, J. Roozenbeek, M. Basol, S. van der Linden, Long-term effectiveness of inoculation against misinformation: Three longitudinal experiments. *J. Exp. Psychol. Appl.* **27**, 1–16 (2021).

47. M. Basol, J. Roozenbeek, M. Berriche, F. Uenal, W. McClanahan, S. van der Linden, Towards psychological herd immunity: Cross-cultural evidence for two prebunking interventions against COVID-19 misinformation. *Big Data Soc.* **8**, (2021).

48. K. A. Parker, S. A. Rains, B. Ivanov, Examining the "blanket of protection" conferred by inoculation: The effects of inoculation messages on the cross-protection of related attitudes. *Commun. Monogr.* **83**, 49–68 (2016).

49. J. A. Banas, S. A. Rains, A meta-analysis of research on inoculation theory. *Commun. Monogr.* **77**, 281–311 (2010).

50. M. E. Toplak, R. F. West, K. E. Stanovich, The cognitive reflection test as a predictor of performance on heuristics-and-biases tasks. *Mem. Cogn.* **39**, 1275–1289 (2011).

51. L. M. L. Schwartz, S. S. Woloshin, W. C. W. Black, H. G. H. Welch, The role of numeracy in understanding the benefit of screening mammography. *Ann. Intern. Med.* **127**, 966–972 (1997).

52. A. J. Wright, S. C. L. Whitwell, C. Takeichi, M. Hankins, T. M. Marteau, The impact of numeracy on reactions to different graphic risk presentation formats: An experimental analogue study. *Br. J. Health Psychol.* **14**, 107–125 (2009).

53. G. Pennycook, C. Martel, D. G. Rand, Knowing how fake news preys on your emotions can help you spot it. *CBC.ca* (2019); www.cbc.ca/news/canada/saskatchewan/analysis-fake-news-appeals-to-emotion-1.5274207.

54. G. Pennycook, J. McPhetres, Y. Zhang, J. G. Lu, D. G. Rand, Fighting COVID-19 misinformation on social media: experimental evidence for a scalable accuracy-nudge intervention. *Psychol. Sci.* **31**, 770–780 (2020).

55. J. van Doorn, D. van den Bergh, U. Böhm, F. Dablander, K. Derks, T. Draws, A. Etz, N. J. Evans, Q. F. Gronau, J. M. Haaf, M. Hinne, Š. Kucharský, A. Ly, M. Marsman, D. Matzke, A. R. K. N. Gupta, A. Sarafoglou, A. Stefan, J. G. Voelkel, E.-J. Wagenmakers, The JASP guidelines for conducting and reporting a Bayesian analysis. *Psychon. Bull. Rev.* 813–826 (2021).

56. M. Berriche, S. Altay, Internet users engage more with phatic posts than with health misinformation on Facebook. *Humanit. Soc. Sci. Commun.* **6**, 1–9 (2020).

57. E. Fast, B. Chen, M. S. Bernstein, Empath: Understanding topic signals in large-scale text, in *Proceedings of the 2016 CHI Conference on Human Factors in Computing Systems* (San Jose, California, 7 to 12 May 2016), pp. 4647–4657.
